# Supplementary material for: Barriers and facilitators of physical activity in knee and hip osteoarthritis: a systematic review of qualitative evidence
Source: BMJ Open. 2017 Dec 26;7(12):e017042. doi: 10.1136/bmjopen-2017-017042 (PMC5770915; doi:10.1136/bmjopen-2017-017042)
Supplement: Supplementary material 1 [file bmjopen-2017-017042supp001.pdf]

Supplement 1. PRISMA checklist of items to include when reporting a systematic review or meta-analysis

Section/topic	Checklist item	Reported on page #
**TITLE**		
Title	Identify the report as a systematic review, meta-analysis, or both.	1
**ABSTRACT**		
Structured summary	Provide a structured summary including, as applicable: background; objectives; data sources; study eligibility criteria, participants, and interventions; study appraisal and synthesis methods; results; limitations; conclusions and implications of key findings; systematic review registration number.	1
**INTRODUCTION**		
Rationale	Describe the rationale for the review in the context of what is already known.	2
Objectives	Provide an explicit statement of questions being addressed with reference to participants, interventions, comparisons, outcomes, and study design (PICOS).	3
**METHODS**		
Protocol and registration	Indicate if a review protocol exists, if and where it can be accessed (e.g., Web address), and, if available, provide registration information including registration number.	3
Eligibility criteria	Specify study characteristics (e.g., PICOS, length of follow-up) and report characteristics (e.g., years considered, language, publication status) used as criteria for eligibility, giving rationale.	3
Information sources	Describe all information sources (e.g., databases with dates of coverage, contact with study authors to identify additional studies) in the search and date last searched.	3
Search	Present full electronic search strategy for at least one database, including any limits used, such that it could be repeated.	3

Section/topic	Checklist item	Reported on page #
Study selection	State the process for selecting studies (i.e., screening, eligibility, included in systematic review, and, if applicable, included in the meta-analysis).	4
Data collection process	Describe method of data extraction from reports (e.g., piloted forms, independently, in duplicate) and any processes for obtaining and confirming data from investigators.	4
Data items	List and define all variables for which data were sought (e.g., PICOS, funding sources) and any assumptions and simplifications made.	4
Risk of bias in individual studies	Describe methods used for assessing risk of bias of individual studies (including specification of whether this was done at the study or outcome level), and how this information is to be used in any data synthesis.	n/a, 2.8 Quality appraisal is reported as relevant to a qualitative SR #4
Summary measures	State the principal summary measures (e.g., risk ratio, difference in means).	n/a, 2.9 Phenomenon of interest is stated as relevant to a qualitative SR #4
Synthesis of results	Describe the methods of handling data and combining results of studies, if done, including measures of consistency (e.g., I^2) for each meta-analysis.	4-5
Risk of bias across studies	Specify any assessment of risk of bias that may affect the cumulative evidence (e.g., publication bias, selective reporting within studies).	n/a, further details are reported under section Amendments to the SR protocol #14 and supplement 4
Additional analyses	Describe methods of additional analyses (e.g., sensitivity or subgroup analyses, meta-regression), if done, indicating which were pre-specified.	5
**RESULTS**		

Section/topic	Checklist item	Reported on page #
Study selection	Give numbers of studies screened, assessed for eligibility, and included in the review, with reasons for exclusions at each stage, ideally with a flow diagram.	5
Study characteristics	For each study, present characteristics for which data were extracted (e.g., study size, PICOS, follow-up period) and provide the citations.	5
Risk of bias within studies	Present data on risk of bias of each study and, if available, any outcome-level assessment (see Item 12).	n/a, 3.3 Study appraisal is reported, #7
Results of individual studies	For all outcomes considered (benefits or harms), present, for each study: (a) simple summary data for each intervention group and (b) effect estimates and confidence intervals, ideally with a forest plot.	6, Table 1
Synthesis of results	Present results of each meta-analysis done, including confidence intervals and measures of consistency.	7-11
Risk of bias across studies	Present results of any assessment of risk of bias across studies (see Item 15).	n/a
Additional analysis	Give results of additional analyses, if done (e.g., sensitivity or subgroup analyses, meta-regression [see Item 16]).	n/a
**DISCUSSION**		
Summary of evidence	Summarize the main findings including the strength of evidence for each main outcome; consider their relevance to key groups (e.g., health care providers, users, and policy makers).	12-14
Limitations	Discuss limitations at study and outcome level (e.g., risk of bias), and at review level (e.g., incomplete retrieval of identified research, reporting bias).	14
Conclusions	Provide a general interpretation of the results in the context of other evidence, and implications for future research.	14
**FUNDING**		
Funding	Describe sources of funding for the systematic review and other support (e.g., supply of data); role of funders for the systematic review.	14
