# Supplementary material for: Barriers and facilitators of physical activity in knee and hip osteoarthritis: a systematic review of qualitative evidence
Source: BMJ Open. 2017 Dec 26;7(12):e017042. doi: 10.1136/bmjopen-2017-017042 (PMC5770915; doi:10.1136/bmjopen-2017-017042)
Supplement: Supplementary material 3 [file bmjopen-2017-017042supp003.pdf]

Supplement 3. Findings: themes and supporting references.

1. Physical health

Barriers (#studies, #references)	References	Facilitators (#studies, #references)	References
Physical barriers and limitations<br>(9, 94)	*While pain was not attributed to their participation in the intervention the pain was described as having a major impact on their perceived opportunity to be physically active at present.* (Hammer, 2015)<br>*All participants discussed experiencing intense physical pain on a daily basis, and how it negatively affected their desire to be active... In addition to these limitations, participants spoke of fatiguing rapidly, which made considering physical activity as more of a challenge* (Stone & Baker, 2015)<br>*Stiffness and fatigue were barriers to exercising. "It was like my body was made of lead"* (Petursdottir, 2010)<br>*Two participants, who were both hikers, reported limiting effects of OA knee pain... A grandmother shared fears and concerns regarding dropping or falling on her grandchildren due to both hand and knee pain.* (Kabel, 2014)<br>*'But as soon as someone says 'let's go for a walk....' It's the last thing I want to do because it hurts too much ...'* (Kaptein, 2013)<br>*'...the day after I just couldn't cope, I was in so much pain'* (Fisken, 2015)<br>*'Exercise hurts. The pain was almost unbearable but I still carried on. Yes, it was very strenuous, but that's how it is, the pain becomes increasingly worse, I think...it just becomes more and more painful.'* (Thorstenson, 2006)<br>*Vi, Hilary, Ethel and Eileen all mentioned their being overweight as contributing to their knee symptom.* (Campbell, 2001)<br>*Ability was also limited by a perceived general lack of physical fitness, sometimes attributed to old age.* (Hendry, 2006)	PA for mobility, symptom relief and health<br>(9, 34)	*Some informants even expressed how their PA maintenance was partly motivated by the belief that PA could help them to postpone or maybe avoid surgery* (Hammer et al., 2015).<br>*"The main motivation to do all this is to prevent an operation to get a new hip" (participant with long-term goal)* (Veenhof et al., 2006)<br>*"I realised my mobility would get worse if I didn't do something about it so I started exercising". (2, 3, 20, 25)* (Hendry et al., 2006)<br>*"I feel like the Tin Man- that if I stop moving, I'll rust up and that will be it"* (Kaptein et al., 2013)<br>*As with the pain, however, the experience of less stiffness and more stamina turned out to be facilitating.* (Petursdottir et al., 2010)<br>*"The physiotherapist professionally guided me to feel less pain. It made me want to do exercises on my own."* (Stone & Baker et al., 2015)<br>*The perceived severity of knee symptoms was an important factor in motivation, with those experiencing severe pain and/or loss of mobility being most likely to continue to exercise.* (Campbell et al., 2001)<br>*...hip pain was highlighted as a common symptom, and several informants linked a perceived reduction in pain to their increased PA level, which represented an important incentive to maintain PA post-intervention.* (Hammer et al., 2015)<br>"Well, it is different now because, as I've already said, previously you exercised to maintain your level of fitness whereas now you exercise in order to regain your physical condition..." (Thorstenson et al., 2006)<br>*"Strenghtening your muscles..keeping your weight down...keeps you in shape"* (Fisken et al., 2015)<br>Disconfirming case: *Some participants who scored high on the Patient Global Assessment (eg, because they perceived less pain) did not continue with their activities, while some participants who scored low on the Patient Global Assessment (eg, because their pain remained the same) reported that their level of activities had increased considerably.* (Veenhof et al., 2006)

2. Intrapersonal factors: themes and references.

Major theme	Barriers (#studies, #references)	References	Facilitators (#studies, #references)	References
Experience and beliefs about PA and OA	PA as non-effective, harmful or of doubtful effectiveness (6, 36)	*Experiencing pain while exercising made it difficult to decide whether it was beneficial or counterproductive.* (Thorstenson, 2006)<br>*[Counter-advice or no recommendations] created further confusion about physical activity and the potential benefits for osteoarthritis.* (Stone & Baker, 2015)<br>*Many participants were worried that exercise was wearing out their joints...* (Hendry, 2006)<br>*..many participants* [were] *uncertain whether PA was good or bad for them when they have arthritis.* (Kaptein, 2013).<br>*If, however, the benefits of the physiotherapy were not perceived as sufficient…non-compliance was a rational outcome…* (Campbell, 2001)<br>*The two informants who had not managed to maintain an increased level of PA expressed how they had hoped for an improvement in their hip specific symptoms, which none of them had achieved.* (Hammer, 2015)	PA as beneficial (7, 60)	*They continued to undertake exercises… from which they perceived they would derive the most benefit.* (Campbell, 2001)<br>*"Keeps the body moving, takes your mind off it, it's good to be outside. Yea, keeping active, or else if you've got osteo, it can get you right down… "* (Fisken, 2015)<br>[Among maintainers] *it was generally described how PA, in addition to the physical effect, also significantly contributed to their psychological well-being.* (Hammer, 2015)<br>*They* [maintainers] *were more likely to have noticed beneficial effects on their OA knee, or general health and well-being as a result of exercise.* (Hendry, 2006)<br>*Other participants were motivated by the results of the exercise, not because they liked it or enjoyed it.* (Petursdottir, 2010)<br>*The informants expressed satisfaction and were convinced of the effectiveness of exercise.* (Thorstenson, 2006)<br>*"I really know these exercises have beneficial effects and that motivates me to continue with my exercises"* (Veenhof, 2006)
	OA beliefs (5, 17)	*"There's no cure, only pain relief"* (Hendry, 2006)<br>*"There is nothing that can be done about OA; therefore, I do nothing"* (Petursdottir, 2010)<br>*"...exercise can help, I am convinced about that, although it did not work for me...If one had started to exercise five or six years earlier, it might have helped."* (Thorstenson, 2006)<br>Another said, *"… if you're a very active person, especially professional athletes … they basically tell you you'll have arthritis when you get older."* (Kaptein, 2013)<br>*"I was having trouble with my knees every so often it did hurt you know with one thing and another. Working in the construction industry there is a lot of lifting and a lot kneeling you see and I felt well I wonder if that's got anything to do with it."* (Campbell, 2001)	Knowledge about PA (4, 15)	*It was described how increased knowledge and information about PA had led to an increased awareness of exercising and of doing this at a certain intensity and frequency…* (Hammer, 2015)<br>*Most of the participants had experienced being educated by their physical therapists.* (Petursdottir 2010)<br>*..many* [participants] *were unaware of specific osteoarthritis-related benefits and unsure of what activities would provide optimal self-management.* (Stone & Baker, 2015)<br>*Overall, most informants understood and acknowledged, but many undertook only a limited programme of exercise.* (Campbell, 2002)<br>[To experience coherence] *This conception contained statements about connecting knowledge about osteoarthritis with knowledge and experiences of exercise.* (Thorstenson, 2006)<br>Disconfirming case: *"You are in a vicious circle where you become less and less active, and the bones are grinding more and more due to muscle weakness. I could see that and I could understand it, but the knowledge has not helped me"* (Hammer, 2015)

Behavioural regulation and attitude	Resigned to OA (5, 10)	*Those who thought that arthritis was caused by immutable factors such as age, obesity and "wear and tear", tended to have a resigned attitude towards their arthritis.* (Campbell, 2001)<br>*...others had become resigned to their physical limitations... "I've accepted my limitations and said goodbye to going out."* (Hendry, 2006)<br>*"There is nothing that can be done about the OA; therefore, I do nothing"* (Petursdottir, 2010)<br>*...osteoarthritis-related pain can lead to disabling thoughts, which are precursors for adopting passive coping and learned helplessness.* (Stone & Baker, 2015)<br>*"... If one had started to exercise five or six years earlier, it might have helped."* (Thorstenson, 2006)	Keep going despite OA (7, 18)	*...those most likely to be continued compliers tended to believe that although there was no cure for arthritis, there were things they could do to minimise its impact, including the physiotherapy* (Campbell, 2002)<br>*Some participants were determined to take control of their disability and used exercise as a means of actively maintaining or improving their mobility. "I'm determined not to let my knee problem stop me from doing the things I want to do."* (Hendry, 2006)<br>*[To be prepared to persevere...] "I played 18 holes of golf and that is also quality of life. I refuse to sit a t home and navel gaze, I just won't"* (Thorstenson, 2006)<br>*"I worked out new ways to cope, to keep my arthritis from getting in the way too much"... They described the importance of not letting the OA control their lives, although its existence should be recognized and respected.* (Petursdottir, 2010).<br>*It appeared that all adherent participants were initially motivated to reach long-term goals.* (Veenhof, 2006)<br>*One participant shared that she continued to be physically active in her community, although she was concerned that others perceived her as being far older than her chronological age.* (Kabel, 2014).<br>*Occasionally participants mentioned adding new activities to their lives: "I learned how to ski about eight years ago. I always wanted to do it and I thought I'm not going to let this get me down"* (Kaptein, 2013)
	Lack of motivation (6, 14)	*"I suppose if there was a really good reason I would* [be strongly disciplined]*."* (Campbell, 2001)<br>*[The two non-maintainers also described obstacles for post-intervention PA...] the other described feeling a lack of motivation towards PA.* (Hammer, 2015)<br>[Reasons for not finding time to exercise...] *others freely admitted to being lazy or lacking motivation.* (Hendry, 2006)<br>*One of the participants seemed to lack the motivation to exercise, based on an overwhelming experience of boredom while exercising. She declared that she would never, ever exercise, no matter what. "It is dead boring, so I just don't do it and never will"* (Petursdottir, 2010)<br>*"You need to have the will to do it... when you are well you don't do it, and when you need to do it, then it hurts and therefore you don't do it (laughter)."* (Thorstenson, 2006)<br>*...all non-adherent participants reported a short-term initial goal or had no specific goal.* (Veenhof, 2006)	Adjustments, prioritization and personal effort (9, 41)	*The majority of informants described how they regularly adjusted their exercises and intensity in an attempt to strike a balance between continuously increasing intensity while at the same time considering the experienced pain.* (Hammer, 2015)<br>*They were eager to find activities and exercise that fitted them and, in many cases, adapted their exercises to their life with OA.* (Petursdottir, 2010).<br>*'My knees were getting really bad and I, so thought, well the only thing I can do really is to do aqua, which I did and I love it'* (Fisken, 2015).<br>*Prioritising exercise and making it part of a weekly routine helped some people to maintain their exercise habit. "... I try and say, OK well I'll go there [gym], have a shower and go shopping... I try to fit it in."* (Hendry, 2006)<br>*More important* [in increasing motivation] *was the willingness and ability to accommodate the exercises into everyday life.* (Campbell, 2001)<br>*"I continue with my exercises, they are integrated in my daily living."* (Veenhof, 2006)<br>*In order to deal with limited time and energy, many participants made tradeoffs. I've had to choose ... where I put my energy, and I know that some days I feel that all I've done is work, so that's kind of a bummer"* (Kaptein, 2013)<br>*He engaged in modified activity, not playing as aggressively as he wanted to, to avoid pain but did not opt out of the activity completely.* (Kabel, 2014)<br>*"Well I suppose to some extent it is up to yourself how much effort you wish to put into it, ... if I don't want to do anything then I don't think I ll benefit from any treatment. I suppose that at the end of the day the outcome of the treatment depends on no one but myself"* (Thorstenson, 2006)<br>Disconfirming case: *Later in their interviews both went on to admit some personal responsibility for their lack of compliance... "It's just excuses when it comes down to basics. I mean you know you could get up in the morning and do it between 6 or 7 or something like that."* (Cambpell, 2001)
	Lacking behavioural regulation (4, 23)	*Those who ceased exercising often cited conflict with regular routines to explain why continuing with exercises was not possible.* (Campbell, 2001)<br>*For others finding time to exercise was a low priority... "when I'm busy I forget."* (Hendry 2006)<br>*Despite recognising the importance of PA, it was considered optional or discretionary compared to essential roles such as work and family.*(Kaptein, 2013)<br>*"One is so occupied that it is very easy not to find time for exercise. Everything else takes precedence."* (Thorstenson, 2006)		

Emotions	OA-related distress (6, 23)	*6 of the 10 participants self-identified as having some type of embarrassment-related experience, usually general embarrassment and frustration over their physical limitations due to the OA pain.* (Kabel, 2014)<br>*Participants expressed depressing thoughts, referring to osteoarthritis as "mentally agonizing".* (Stone & Baker, 2015)<br>*"I don't know if you can imagine how it is to be confronted with things that you want to do but you are unable to. That is mentally stressful."* (Hammer, 2015)<br>*A few of the women mentioned 'paralyzing fatigue' as a major barrier for getting anything done and felt it might be related more to mental fatigue...* (Petursdottir, 2010)<br>*A few individuals noted a loss of their identity as an athletic or physically active person... they often tried to hide difficulties with activities from others.* (Kaptein, 2013)<br>*"It got worse and worse and I started falling down ... it's so embarrassing."* (Campbell, 2001)	Enjoyment (4, 22)	*Not surprisingly, people who enjoyed exercising were likely to continue; those that disliked it stopped. "I really do enjoy the gym; I look forward to going."* (Hendry, 2006)<br>*Some participants based their motivation on the fact that they liked PA and therefore had been physically active. "I have always enjoyed physical activity"* (Petursdottir, 2010)<br>*"The buoyancy... I like deep water... It takes the impact off your joints... it gives you freedom... if you've been sedentary and not able to move around... the water makes you feel wonderful"'* (Fisken, 2015).<br>*"I feel such a fool standing on one leg and going up and down on my own and I tends to drop it I do."* [non-maintainer] (Campbell, 2001)

3a. Social Environment: themes and references.

Major theme	Barriers (#studies, #references)	References	Facilitators (#studies, #references)	References
Health professionals	Lack of support from health professionals (5, 22)	*Sometimes the advice was vague or absent... Occasionally exercise was discouraged.* (Hendry et al., 2006)<br>*"So I go to the doctor and all he just simply done was put his hand on my knee, he said 'move your leg,... you are getting old, you've got rheumatism.'"* (Campbell et al., 2001)<br>*...physicians often provided them with counter advice or did not offer any recommendations...* (Stone & Baker, 2015)<br>*"They have not done it [encouraged exercising]"* (Petursdottir et al., 2010)<br>*"The instructor was not geared up for my particular disability [OA]... and I found it very stressful"* (Fisken et al., 2015)	Support from health professionals (8, 50)	*Advice from health professionals was mainly in favour of exercise and consisted of encouragement to exercise, advice about specific exercises, and referral to a gym. (Hendry et al., 2006)*<br>*The supervision by physical therapists highly influenced the informants' ability to progress in training intensity as the physical therapists verbally expressed their confidence in the participants and exhibited realistic expectations about their exercise abilities* (Hammer et al., 2015)<br>*All participants spoke about the instrumental role of health care providers in influencing and encouraging physical activity.* (Stone & Baker, 2015)<br>*"Well, I always say that my physical therapist is as good as any psychologist."* (Petursdottir et al., 2010)<br>*Overall, most informants understood and acknowledged, as they were instructed by the physiotherapist, that they should do the exercises often and regularly, but many undertook only a limited programme of exercise.* (Campbell et al., 2001)<br>*It appeared that all adherent participants reported that... the physiotherapists had a coaching role during intervention.* (Veenhof et al., 2006)<br>*"I think that [an instructor] is good because then you learn what to do so that you do not do it in the wrong way."* (Thorstenson et al., 2006)<br>*"...knowing that aqua is for people possibly who have arthritis... they ought to have.. an extra training course or something to fit, to accommodate that"* (Fisken et al., 2015)
Social support	Social comparison as demotivating (5, 15)	*Comparison with others with more limiting disease or a stoic attitude to knee symptoms all seemed to be associated with an attenuation of the motivation to comply"* (Campbell et al., 2001)<br>*"I found it very stressful to be honest because I felt like I had to do the same as the others and keep up..."*(Fisken et al., 2015).<br>*"They don't want to be dragged down by somebody that's not up to their standard I would think."* (Hendry et al., 2006)<br>*"I couldn't keep up with everyone else and felt like I was dragging them*	Social support facilitating PA (7, 43)	*The majority of informants described how they continued to exercise with others because of the mutual support and encouragement they hereby achieved...* (Hammer, 2015)<br>*The support, caring, and encouragement of others were among important external factors influencing how much the participants exercised.* (Petursdottir, 2010)<br>*"I think it's important to be with other people, how other people cope and that you're not alone and there are other people you know, in similar situations."* (Fisken, 2015).<br>*"I like the gym referral scheme because you're in a group of people who all have*

		behind." (Kabel et al., 2014)<br>Disconfirming case: *Participants also gave examples of persisting with a painful activity and risking intensifying the pain because of social pressure or the desire to avoid embarrassment and disapproval...* (Kabel et al., 2014).		*problems."* (Hendry, 2006)<br>*Eileen explained how difficult it was to continue the exercises programme since she stopped seeing the physiotherapist.* (Campbell, 2001)<br>*An important facilitator of PA and a strategy that helped some participants 'stay in the game' was having social support...* (Kaptein, 2013)<br>*"One of my friends who knows about my arthritis asked me if I ever exercise...Then she said she would work out with me if I wanted to. That was the first time I ever seriously thought about exercising.* (Stone & Baker, 2015)
	Lack of social support (4, 8)	[Sedentary informants] *had been given scant encouragement to exercise.* (Hendry et al., 2006) [Regarding family's attitudes] *some of the women expressed having a hard time justifying to themselves and their families their need to spend time exercising.* (Petursdottir et al., 2010).<br>*Not only* [about half of the participants] *did not receive support from others to manage physically demanding activities at work, they often tried to hide difficulties with activities from others.* (Kaptein et al., 2013)<br>*"If perhaps my wife would work with me and you had a bit of competition..."* (Campbell et al., 2001)		
