# Supplementary material for: Barriers and facilitators of physical activity in knee and hip osteoarthritis: a systematic review of qualitative evidence
Source: BMJ Open. 2017 Dec 26;7(12):e017042. doi: 10.1136/bmjopen-2017-017042 (PMC5770915; doi:10.1136/bmjopen-2017-017042)
Supplement: Author's manuscript [file bmjopen-2017-017042.draft_revisions.pdf]

BMJ Open is committed to open peer review. As part of this commitment we make the peer review history of every article we publish publicly available.

When an article is published we post the peer reviewers' comments and the authors' responses online. We also post the versions of the paper that were used during peer review. These are the versions that the peer review comments apply to.

The versions of the paper that follow are the versions that were submitted during the peer review process. They are not the versions of record or the final published versions. They should not be cited or distributed as the published version of this manuscript.

BMJ Open is an open access journal and the full, final, typeset and author-corrected version of record of the manuscript is available on our site with no access controls, subscription charges or pay-per-view fees (http://bmjopen.bmj.com).

If you have any questions on BMJ Open's open peer review process please email editorial.bmjopen@bmj.com

BMJ Open

Barriers and facilitators of physical activity in knee and hip osteoarthritis. A systematic review of qualitative evidence.

Journal:	*BMJ Open*
Manuscript ID	bmjopen-2017-017042
Article Type:	Research
Date Submitted by the Author:	28-Mar-2017
Complete List of Authors:	Kanavaki, Archontissa; University of Birmingham, School of Sport, Exercise and Rehabilitation Sciences<br>Rushton, Alison; University of Birmingham<br>Efstathiou, Nikolaos ; University of Birmingham, Nursing, School of Medicine<br>Alrushud, Asma; Unoversity of Birmingham<br>Klocke, Rainer; 2 Dudley Group NHS Foundation Trust, Department of Rheumatology<br>Abhishek, A; University of Nottingham<br>Duda, Joan; University of Birmingham, Sport, Exercise & Rehabilitation Sciences
**Primary Subject Heading**:	Sports and exercise medicine
Secondary Subject Heading:	Rheumatology
Keywords:	osteoarthritis, systematic review, barriers, facilitators, physical activity

SCHOLARONE™
Manuscripts

For peer review only

Barriers and facilitators of physical activity in knee and hip osteoarthritis. A systematic review of qualitative evidence.

Archontissa M. Kanavaki[1], Alison Rushton[1,2], Nikolaos Efstathiou[3], Asma Alrushud[1], Rainer Klocke[4], Abhishek Abhishek[5], Joan L. Duda[1]

[1]School of Sport, Exercise and Rehabilitation Sciences, College of Life and Environmental Sciences, University of Birmingham and MRC-Arthritis Research UK Centre for Musculoskeletal Ageing Research, University of Birmingham, Birmingham, UK, B152TT

[2]Centre of Precision Rehabilitation for Spinal Pain (CPR Spine), University of Birmingham, Birmingham, UK

[3]Nursing, Institute of Clinical Sciences, Medical School, University of Birmingham, Birmingham, UK, B152TT

[4]Department of Rheumatology, Dudley Group NHS Foundation Trust, Dudley, UK, DY1 2HQ

[5]Academic Rheumatology Unit, School of Medicine, Faculty of Medicine and Health Sciences, University of Nottingham, Nottingham, UK, NG7 2RD

Corresponding author: Archontissa M. Kanavaki, School of Sport, Exercise and Rehabilitation Sciences, University of Birmingham, Edgbaston, Birmingham, B15 2TT, UK. Email: amk377@bham.ac.uk

ABSTRACT

Background: Physical activity (PA) has a key role in the management of hip and knee osteoarthritis (OA), however maintaining a physically active lifestyle is a challenge for people with OA. The determinants of PA in this population need to be understood better so that they can be optimised by healthcare interventions and social policy changes.

Objectives: To conduct a systematic review of the existing qualitative evidence on barriers and facilitators of PA for patients with hip or knee OA. Secondary objectives, to explore differences in barriers and facilitators between (i) lifestyle PA and exercise; (ii) PA uptake and maintenance.

Methods: MEDLINE, EMBASE, Web of Science, CINAHL, SPORTDiscus, Scopus, Grey literature and qualitative journals were searched. CASP-Qualitative checklist and Lincoln & Guba's criteria were used for quality appraisal. Thematic synthesis was applied.

Findings: Ten studies were included. The findings showed a good fit with the biopsychosocial model of health. Aiming at symptom relief and mobility, positive PA experiences and beliefs, knowledge, a "keep going" attitude, adjusting and prioritising PA, having health-care professionals' and social support, emerged as PA facilitators. Pain and physical limitations; non-positive PA experiences, beliefs and information; OA-related distress; a resigned attitude; lack of motivation, behavioural regulation, professional support; and negative social comparison with co-exercisers were PA barriers. Paucity of data did not allow for the secondary objectives to be explored.

Conclusion: Our findings reveal a complex interplay among physical, intrapersonal (including psychological) and social-environmental factors corresponding to the facilitation and hindrance of PA engagement. Further research is required to find out the efficacy of individualized patient education, psychological interventions, or social policy change to promote PA in individuals with lower limb OA.

Trial registration number CRD42016030024

Keywords: osteoarthritis, physical activity, systematic review, barriers, facilitators

Word count: 3.877

For peer review only

Strengths and limitations

- This systematic review is the first to identify, appraise and synthesise the existing qualitative research on barriers and facilitators to PA in knee and hip osteoarthritis.
- Rigorous methods have been applied, informed by the Centre for Reviews and Dissemination and Cochrane Qualitative Research Methods Group guidelines and reported according to the Preferred Reporting Items for Systematic Reviews and Meta-Analyses (PRISMA) and Enhancing Transparency in Reporting the Synthesis of Qualitative Research (ENTREQ) statements.
- Papers written in English language only were included.

Osteoarthritis (OA) is the commonest joint disease and main cause of disability in older adults[1]. OA management focuses on analgesia and non-pharmacological modalities such as exercise and weight loss[2]. Exercise, i.e. structured and purposeful physical activity (PA)[3], reduces pain and improves function in people with knee or hip OA[4-9]. However, despite the positive effects on symptoms, exercise interventions do not promote sustained behavior change [10 11]. Just like exercise, PA associates with better physical function[12-14], and even modest increase in PA (from sedentary to light intensity PA) improves arthritis pain[15]. At the population level, it is simpler to promote PA in people with painful OA e.g. via radio and television, than promoting exercise as that will require a larger behavior change and may need consultation with trained physiotherapists. However, existing evidence suggests that people with lower limb OA have such low PA levels that they gain no health benefits from it[16-18]. Additionally, despite the positive effects on symptoms, exercise interventions do not promote sustained behavior change[10 11]. Thus, there is need to understand the determinants of reduced PA in people with painful OA so that these can be optimised to promote PA.

The disease specific determinants of PA in those with lower limb OA e.g. symptom severity, physical function [19-23] are relatively well understood, but the psychological, social and environmental determinants of PA in OA have not been adequately examined[21 22]. Understanding these factors is of great importance as pain makes PA an aversive experience leading to activity avoidance[24-27] and pain is influenced by psychological and environmental factors[25 28-30]. A recent scoping review identified several psychological and environmental barriers and facilitators of exercise in people with hip or knee OA[23]. However, scoping reviews lack the methodological rigor of SRs[31]. A SR of qualitative data holds promise for a thorough and in-depth understanding of the modifiable psychosocial factors predicting PA behaviour.

For peer review only - http://bmjopen.bmj.com/site/about/guidelines.xhtml

The objectives of this study were to identify, appraise and synthesise the existing qualitative evidence on barriers and facilitators to PA in hip or knee OA, and explore differences in barriers and facilitators between lifestyle PA accrued in daily activities, and exercise; and between PA uptake and maintenance.

METHODS

This SR was registered with the International Prospective Register of SRs (CRD42016030024) and its protocol reported previously[32]. The reporting follows the Preferred Reporting Items for SRs and Meta-Analyses (PRISMA) and the Enhancing Transparency in Reporting the Synthesis of Qualitative Research (ENTREQ) statements (Supplement 1).

PICOs were adapted to inform eligibility.

Population Study participants were adults with physician diagnosed or radiographic (Kellgren and Lawrence grade ≥2) hip or knee OA, or met classification criteria for OA at these joints [33]. If a study included people with other arthritis, e.g. rheumatoid arthritis, they were included if people with knee or hip OA were the largest proportion. Studies with participants awaiting total joint replacement were excluded.

Outcomes: perceptions of barriers and facilitators that influence uptake or maintenance of PA. Studies were included if they explored the factors/barriers/facilitators/motivation to engagement in PA, or addressed the experience of people with hip or knee OA regarding PA or exercise.

Study designs: Qualitative or mixed methods studies.

Language: Published in English.

Information sources

MEDLINE (Ovid MEDLINE(R) In-Process & Other Non-Indexed Citations and Ovid MEDLINE(R) 1946 to Present, OVID), EMBASE (1974 onwards, OVID interface), PhychINFO (1967 onwards, OVID), Web of Science, CINAHL, SPORTDiscus and Scopus were searched up to 31 of December 2015. Grey literature sources were considered, i.e. OpenGrey, NHS evidence. The search strategy was complemented by hand search of qualitative-research-centred journals screening of references of included articles and contacting researchers active in the field.

Search

The search strategy contained exhaustive keyword combinations for each of the four concepts of interest, i.e. knee or hip OA; PA/exercise; facilitators, barriers, motivation, uptake, maintenance; qualitative studies (Supplement 2).

For peer review only - http://bmjopen.bmj.com/site/about/guidelines.xhtml

Study selection

The search and study selection was conducted by two researchers independently (AMK & AsA). Endnote X7 was used for data management. Citations and abstracts were imported and duplicates removed. After title/abstract screening, full text of potentially relevant studies were assessed and additional information was sought from authors where necessary. If consensus was not reached between the two researchers, a third reviewer was consulted (AR).

Data items

All text under the sections of "results" and "findings" was considered as data. Where findings and discussion were presented together, the whole section was considered for analysis.

Data collection process

Data items were entered into and managed with NVivo 11 qualitative data analysis software (QSR International).

Quality appraisal

Quality appraisal assessed the reporting, methodological rigor and conceptual consistency of the included studies [34] to identify and discard low quality studies. Two approaches were used, which complement each other [32]:

(a) the Critical Appraisal Skills Programme (CASP) Qualitative Checklist [35] (Studies were rated as high, medium and low quality if they met ≥8, 5-7, and 4 or fewer criteria respectively);

(b) the evaluative criteria of credibility, transferability, dependability and confirmability that assess the trustworthiness of the study. Studies were rated high, medium, and low quality if they met ≥3, 2, or 1 and less criteria[36] .

Two reviewers independently appraised the selected studies (AK, NE).

Phenomenon of interest

The phenomenon of interest was the description and interpretation of OA patients' perceptions and experiences regarding what facilitates, motivates or hinders them from engaging in PA. In addition, differences in facilitators and barriers to uptake and maintenance of PA, exercise and lifestyle PA were also included

.

Synthesis of results

Data was analysed by thematic synthesis[37]. First, authors' interpretations and informants' quotes were coded separately, line by line. Codes of original themes, subthemes and codes clearly referring to other types of

For peer review only - http://bmjopen.bmj.com/site/about/guidelines.xhtml

arthritis where excluded from the synthesis. Next, descriptive themes were formed through code merging and grouping in a highly iterative process, creating a hierarchical tree. To form the analytical themes, a data driven analysis was initially conducted to allow an inductive interpretation. A group (AMK, NE, AR, JLD) review meeting was held and the fit of this synthesis within theoretical models of behaviour change, motivation, human development and health was examined. The findings showed good fit with the biopsychosocial model of health[38] which was chosen to facilitate a more comprehensive and meaningful interpretation of the data and reporting of the findings. The descriptive themes were then re-examined and refined. At this point the research question was introduced to help infer the barriers and facilitators under the three domains of the biopsychosocial model. To enhance the credibility of the findings the synthesis was conducted by AMK and checked independently by NE.

Additional analysis. The descriptive study characteristics were examined in relation to the secondary research objectives. Due to insufficient evidence no further analysis was conducted.

RESULTS

Study selection

5,003 studies were identified, and after removing duplicates, 2,657 titles or/and abstracts were screened and 51 full-text papers were assessed. Seven authors were contacted for further information. Information was not provided for two studies, which were excluded. Ten studies were included [39-48].

Study characteristics

There were 173 participants, mainly middle aged to older, and female. Nine of ten studies reported qualitative methodologies (Table 1).

Table 1. Study characteristics

Study	Objectives	OA Site	Country	Participants (number, characteristics, sampling)	Methods (Data collection & analysis)	Findings	Relevance to Secondary Objectives (Exercise vs lifestyle PA; uptake vs maintenance)
Campbell et al. (2001)[39]	To explore compliance with physiotherapy, i.e. a home based exercise intervention.	Knee	UK	20 participants; 14 female; >45 Maximum variation sample.	Interviews; constant comparative method	Initial compliance was due to a sense of moral oblication towards the physiotherapist. Continued compliance was linked to vieweing exercise as beneficial (precondition), ability and willingness to fit exercises in daily life, perceived symptom severity, arthritis and comodbidity attitudes, existing exercise and OA experiences.	Exercise regime.<br><br>Initial compliance to programme and one year follow up.
Fiskenn et al.	Reasons for ceasing	Various	New Zealand	11 participants, female; >60;	Focus groups; general	Main reported barriers were lack of appropriate	Exercise regime.

For peer review only - http://bmjopen.bmj.com/site/about/guidelines.xhtml

(2015)[40]	participation in aqua based exercise.			recruitment through advertisements in health clinics and medical centers	inductive thematic approach.	classes and knowledgeable instructors, which often led to an increase in pain, cold water and the facilities. Improvements in physical ability and the social component were the key reported benefits.	No distinction (non-maintenance)
Hammer et al. (2015)[41]	Exploring self-efficacy in relation to PA maintenance among maintainers and non-maintainers post-intervention	Hip	Denmark	15 participant; 8 female; mean age 66; community dweling; Criterion based purposeful sampling	Mixed methods. For the qualitative substudy: semi-structured interviews; directed content analysis.	Four pre-defined and one additional theme emerged: mastery experiences, vicarious experiences, verbal persuasion, physiological and emotional states, altruism.	Exercise regimes.<br><br>No distinction. Maintainers and non-maintainers.
Hendry et al. (2006)[42]	Explore primary care patients' views towards exercise and factors that determine acceptability and motivation to exercise; identify barriers that limit its use.	Knee	UK	22 participants; 16 women; age 52-86; Purposive sampling; recruitment from GPs(20) and gymnasium(2)	Interviews and focus group; Principles of Framework method of qualitative analysis.	Three main categories: perception of physical capacity, beliefs about exercise (experience, advice, arthritis aetiology), motivational factors (enjoyment and social support, taking control of disability, priority setting, context). Exercise behaviour typology: long-term sedentary, long-term active, retired form exercise, converted to exercise.	Exercise (broad definition).<br><br>No distinction.
Kabel et al. (2014)[43]	Role of pain, social pressure and embarrassment in activity related decision making.	Knee	USA	10, community dwelling, mean age 60, 7 women, 9 Caucasian	Interviews; Grounded theory or constant comparative method	Embarassment-related experiences regarding PA were linked both to engaging and avoiding activities. Each categorie included two subgoups: individuals avoiding pain and individuals avoiding embarrassment.	PA (living with OA).<br><br>No distinction.
Kaptein et al. (2014)[44]	PA perception in the context of managing arthritis and multiple roles.	(OA & IA) various	Canada	40 (20 OA, hip/knee prevalent group); recruitment from community and arthritis related clinics and groups	Focus groups Qualitative content analysis	PA perceptions were overall positive, yet PA, arthritis and life roles relationship is complex. PA as potential cause of arthritis, their reciprocal relationship, PA harms and benefits, perceived choices reg PA engagement and social support were the overarching themes that emerged in the discussions.	PA<br><br>No distinction.
Petursdottir et al. (2010)[45]	Exercise experience. What determines whether people exercise	various	Iceland	12; 9 women; Purposeful sampling; outpatient clinics and targeted newsletter	Interviews; Phenomenology (Vancouver School)	Internal (individual attributes and exercise experiences) and external (social and physical environment) factors act as both barriers and facilitators in a delineated model.	Exercise<br><br>No distinction.
Stone & Baker (2015)[46]	Facilitators and barriers to regular PA	Hip or knee	Canada	15, Snowball sampling; primary care	Semi-structured interview; Interpretational analysis	Facilitators: pain relief, clear communication from health-care professionals, social support. Barriers: pain, psychological distress, lack of support from health care professionals	PA<br><br>No distinction.
Thorstensson et al. (2006)[47]	Underlying processes leading to response or non-response to exercise as treatment	Knee	Sweden	16, middle aged; Subsample of intervention participants, purposefully chosen	Interviews; Phenomenography	Concerns about exercise as OA treatment. Four descriptive themes: to gain health, to become motivated, to experience the need for support, to experience resistance.	Exercise<br><br>No distinction.
Veenhof et	Factors that	Hip or	Netherlands	12;	Interviews;	Lack of consistency in	Exercise

For peer review only - http://bmjopen.bmj.com/site/about/guidelines.xhtml

al. (2006)[48]	explain differences between patients who integrated activities in their daily lives & those who did not.	knee		Subsample of intervention participants- deliberate sampling for homogeneity	Grounded theory	factors relating to adherence. Long-term goals at the beginning and active involvement in the intervention related to greater adherence.	No distinction.

Appraisal of studies

All included studies were of medium or high quality (Table 2). The research design and data analysis were not clear or well described in half of the studies and very few studies had clearly identified the relationship between the researcher and participants. Credibility, transferability and confirmability were met by almost all studies, although dependability only by two.

Table 2. Appraisal of studies

		Campbell et al. (2001)	Fisken et al. (2015)	Hammer et al. (2015)	Hendry et al. (2006)	Kabel et al. (2014)	Kaptein et al. (2013)	Petursdottir et al. (2010)	Stone & Baker (2015)	Thorstensson et al. (2006)	Veenhof et al. (2006)
**CASP Qualitative Checklist**		6/10	6/10	6/10	9/10	6/10	7/10	9/10	9/10	7/10	6/10
1. Was there a clear statement of the aims of the research?		✓	✓	✓	✓	✓	✓	✓	✓	✓	✓
2. Is a qualitative methodology appropriate?		✓	✓	✓	✓	✓	✓	✓	✓	✓	?
3. Was the research design appropriate to address the aims of the research?		?	✓	✓	✓	?	x	✓	✓	?	?
4. Was the recruitment strategy appropriate to the aims of the research?		✓	?	✓	✓	✓	✓	✓	✓	?	✓
5. Was the data collected in a way that addressed the research issue?		✓	?	x	✓	?	✓	✓	✓	?	✓
6. Has the relationship between researcher and participants been adequately considered?		?	?	x	✓	?	x	✓	?	✓	?
7. Have ethical issues been taken into consideration?		?	✓	✓	?	✓	✓	?	✓	✓	✓
8. Was the data analysis sufficiently rigorous?		?	?	?	✓	?	?	✓	✓	✓	✓
9. Is there a clear statement of findings?		✓	✓	?	✓	✓	✓	✓	✓	✓	✓
10. How valuable is the research?		✓	✓	✓	✓	✓	✓	✓	✓	✓	?
**Trustworthiness**	**Credibility**	✓		✓	✓	✓	✓	✓	✓	✓	✓
	**Transferability**	✓	✓	✓	✓	✓	✓	✓	✓	✓	
	**Dependability**								✓	✓	
	**Confirmability**	✓	✓	✓	✓		✓	✓	✓	✓	✓

✓ *= yes,* x*= no,* ?*= uncertain*

For peer review only - http://bmjopen.bmj.com/site/about/guidelines.xhtml

Synthesis of results

Barriers and facilitators are presented under the three conceptual domains, i.e. physical health, intrapersonal factors and social-environmental factors. Barriers and facilitators that appeared in at least three studies are reported, to keep a balance between richness and applicability of the findings (Table 3; Supplement 3 for supporting references).

Table 3. Barriers and facilitators: Themes, subthemes and number of supporting references

Domain	Major themes	**Barriers**	No of studies	No of ref/ces	**Facilitators**	No of studies	No of ref/ces
Physical health		Physical barriers and limitations (Pain and other symptoms; Perceived functional limitations)	9	94	PA for mobility, symptom relief and health (PA to maintain mobility; PA for symptom relief; PA for health)	9	34
Intrapersonal /psychological factors	Experience and beliefs about PA and OA	PA as non-effective, harmful or of doubtful effectiveness	6	36	PA as beneficial	7	60
		OA beliefs	5	17	Knowledge about PA	4	15
	Behavioural regulation & attitude	Resigned to OA	5	10	Keep going despite OA	7	18
		Lack of motivation	6	14	Adjustments, prioritisation and personal effort (Adjusting PAs; Prioritising PA; Personal responsibility and effort in being physically active)	9	41
		Lacking behavioural regulation	4	23			
	Emotions	OA-related distress	6	23	Enjoyment	4	22
Social Environment	Health professionals	Lack of advice and encouragement from health professionals	5	22	Support from health professionals	8	50
	Social support	Social comparison as demotivating	5	15	Social support facilitating PA	7	43
		Lack of social support	4	8			

1. Physical health

Barriers. Physical barriers and limitations. Pain is aversive, stressful and inherent to living with OA[39-47]. It was mentioned as part of daily experience[45 46] or in relation to particular types of activities[40-42 44 46 47]. Along with fatigue and stiffness[44-46] these symptoms hindered the ability to engage in PA. There was a vicious cycle between symptoms and lack of exercise[41 42]. At an advanced stage of OA, PA was inhibited[42]. OA symptoms were aggravated by obesity and made PA more difficult[39 42 45]. Participants also discussed their sense of limited physical capacities and that one's body cannot manage PA requirements, resulting in loss of previous activity patterns[42-46]. For example, some talked about the need to choose between activities because of limited energy[44]. Old age and lack of physical fitness are also reported as perceived PA barriers[42 45].

For peer review only - http://bmjopen.bmj.com/site/about/guidelines.xhtml

Facilitators. PA for mobility, symptom relief and health. Among those who held a physically active lifestyle maintaining or regaining their mobility was a strong motive for PA[39 41 45 46 48]. In most cases the aim was to keep functioning [39 42 44 47], in some it was so specific as to prevent a joint surgery[41 48]. Pain relief is another strong motive for being physically active and active individuals were more likely those who had experienced pain reduction [39 41 45 46 48]. A few informants presented a "no pain, no maintenance" pattern, where pain cessation was followed by dropping exercise[39 48]. Improvements in other symptoms, such as stiffness and joint stability, were sufficient reasons for being active, even when pain remained[39 45]. Maintaining good general health and physical condition were also reasons for being physically active[41 42 44 45 47]. This facilitator was closely linked to a positive, beneficial PA experience and subsequent positive attitude towards PA, which is a crucial facilitator discussed below.

2. Intrapersonal/ psychological factors

Experience and beliefs about PA. *Facilitators*. PA as beneficial. Experiencing benefits from PA participation, which in most of the studies was related to engagement in an exercise intervention, helped shaping positive PA beliefs and motivated individuals towards continuing PA[39-42 45 47 48]. A sense of psychosomatic well-being was an important component of this theme[40-42 45 47]. Improvement in coping with OA [47] and sleep [45] were mentioned.

Knowledge about PA in OA. Accurate knowledge of the importance of PA in OA, acquired through health care, physiotherapy and exercise interventions, was an important PA facilitator[41 45-47]. It led to awareness regarding PA benefits and helped in making correct interpretations of PA experiences.

Barriers. PA as non-effective, harmful or of doubtful effectiveness. The belief that PA does not help or might further deteriorate their condition, hindered people from being active[39 41 42 44 46 47]. Experiencing activity-related pain in the joint, for example, was often interpreted as PA exacerbating OA, which stemmed from the understanding of OA as a "wear and tear" condition[42 44 47]. Not experiencing the anticipated beneficial effects during exercise interventions was a reason for distrust in PA as an effective means of treatment [39 41 42 47]. Also, early negative experiences with sports resulted in an avoidance towards exercise [45].

OA beliefs. Beliefs that nothing can be done regarding the condition[42 45 47] and that overuse was the cause of OA[39 42 44] were linked to less inclination towards being physically active. In one study the relationship between PA and OA was discussed as bi-directional[44].

Daily activities as PA. This theme revolved around beliefs about non-leisure PA[42 44 45 47]. However, there were no consistent patterns across studies to be clearly classified as barriers or facilitators. For example, non-leisure activities were viewed as a sufficient amount of PA by some[42 45 47] and as insufficient by others[42].

For peer review only - http://bmjopen.bmj.com/site/about/guidelines.xhtml
For peer review only

Behavioural regulation and attitude. *Facilitators.* Keep going despite OA Authors' interpretations related to this concept varied, e.g. determination to take control of arthritis[42], perseverance[47], personality traits of adaptability and initiative[45], belief that there are "things patients can do" about their OA[39], motivation towards long-term goals[48]. The importance of keeping a positive attitude was also discussed[44 45]. In two studies the relevant participant quotes were presented under the themes risking embarrassment[43] and bi-directional impact between PA and arthritis[44].

Adjustments, prioritisation and personal effort. Physically active individuals described how they were making short or long term modifications to their PAs[40-45], such as finding a type of exercise that was suitable for their physical abilities[40-42 45], adjusting PA intensity to their current condition[41 43 45], even changing their job[44]. This task of continuously adjusting PAs was quite demanding[45]. Prioritising PA and fitting it into a routine was mentioned by a number of physically active participants and reflected the importance they assigned to PA[39 42 47 48]. Active participants also acknowledged they were the main agents in managing their condition and they were consciously making efforts to stay active[39 42 44 47].

Barriers. Lack of motivation Participants in different studies referred to a lack of motivation or goal, laziness and boredom towards PA[39 41 42 45 47 48]. However, these concepts were not further explored.

Lacking behavioural regulation. In the face of the demands of other life roles and a busy schedule, especially family related, inactive participants were not prioritising PAs[39 42 44 47]. In two studies informants referred to not finding a PA suitable for their current condition[40 42]. In one study low self-regulation was the reason given for not exercising regularly[42].

Resigned to OA. In half of the studies informants expressed a resigned attitude towards making an effort to be active[39 42 45-47]. Reflecting fatalistic beliefs about OA and feelings of helplessness, this attitude was linked to attenuated motivation for being physically active.

Emotions. *Facilitators.* Enjoyment. Enjoying PA in general or a particular PA mode facilitated its continuation[40 42 45].

Barriers. OA related distress. Living with OA means adjusting to a reality of decreased physical functioning and in several cases participants talked about this experience of giving up activities, being unable to meet life roles and daily demands as distressing or embarrassing[39 41 43-46]. Mental stress[41], extreme unhappiness and paralyzing fatigue[45], feeling broken and mentally depressed[46], weakness[44] were used.

3a. Social Environment

For peer review only - http://bmjopen.bmj.com/site/about/guidelines.xhtml

Health professionals. *Facilitators.* Support from health professionals. Physiotherapists exerted great influence on the patients' PA behaviour[39 41 42 45 46 48]. Providing instructions, education, encouragement and rapport with the patient were means of facilitating PA. PA advice and prescription by doctors was another PA facilitator[42 45]. Supervision during exercise was valued[39-42 47 48]. Good supervision gave participants the reassurance that what they were doing was appropriate and good for their body[42], which they needed[46], and motivated them to exercise[39 41 47 48].

Barriers. Lack of support from health professionals. Ambiguous, no or conflicting information from doctors regarding PA was a barrier[39 42 45 46]. In one study, the instructor not having specialized OA training was the reason that lead participants to discontinue their exercise[40].

Social support. *Facilitators.* Social support facilitating PA . Social support as a facilitator was mainly discussed in the context of exercising in a group, as well as support from family and friends. Feeling comfortable and motivated, even inspired when exercising with people of similar physical abilities and age emerged as an advantage of PA programs[39-42 44 46]. This was of particular importance when someone was first introduced to PA[41]. Opportunities to socialize were also an advantage of group PA[40 42]. In addition, psychological and instrumental support from family and friends emerged as an asset of physically active participants, taking the form of active encouragement, expression of interest and understanding, an exercise buddy or role model[41 44-46]. Community based support was mentioned as PA promoting[46].

Barriers. Social comparison as demotivating. Although this concept did not explicitly appear as an authors' interpretation it emerged from informants' quotes. Being unable to keep up with others when participating in PA was a PA barrier as it provoked feelings of embarrassment and distress[39 40 42 43].

Lack of social support The lack of social support from peers and family as a barrier was discussed in relation to lack of understanding and encouragement from the person's family and social[39 44 45] and work environment[44].

3b. Physical Environment

Barriers. The cost of exercise classes[40 44 45], limited accessibility[45 47] and lack of availability of appropriate modes[45 46], as well as cold weather and issues regarding safety[40] were the reported environmental barriers to PA.

DISCUSSION

This SR is the first to synthesize the existing qualitative research on barriers and facilitators to PA in knee and hip OA. Pain and physical limitations, absence of positive PA experiences and beliefs, resigned attitude and distress due to OA, lack of behavioral regulation, lack of support from health professionals and negative social

For peer review only - http://bmjopen.bmj.com/site/about/guidelines.xhtml

comparisons when exercising in a group were important PA barriers. Symptom relief and mobility, positive PA experiences and beliefs, knowledge, enjoying PA, a "keep going" attitude, adjusting and prioritising PA, having professional and social support were important PA facilitators. Overall the findings are consistent with known PA correlates in exercise psychology[49], theories of behavioral change[50] and findings of existing SRs in general (i.e. non-OA specific) populations that share common characteristics with OA patients[51-53], but also outline a unique profile of PA barriers and facilitators in lower limb OA.

Factors related to physical health, specifically pain and physical function, were the most consistently reported. This indicates that OA has a central role and impact in people's lives and experiences, which is in line with previous qualitative findings that pain discussions by people with OA differ in frequency and quality in comparison to healthy individuals[54]. Importantly, physical barriers are reported both by active and inactive people. Therefore, physical barriers alone cannot explain PA behavior- with the exception of patients at very advanced stages of OA[55]. Intrapersonal and social variables are crucial in PA behavioras reported earlier[53].

The identified barriers and facilitators are not stand-alone entities, but manifest a complex interplay. Personal experience, knowledge and beliefs about PA and OA were interwoven concepts and formed the basis of PA behavior. Experiencing benefits from participation in an exercise program- which was the case in most of the included studies- shapes a positive attitude towards PA[51 52 56-58]. Accurate knowledge regarding PA and OA bolstered a positive interpretation of and predisposition towards PA experience. Viewing pain as manageable versus inevitable elicited different behaviors[59 60] and, not surprisingly, patient education is a core component of health care and OA management[61 62]. Support from health professionals becomes crucial as they can provide rationale and motivation for PA[56] and shape the patients' health experience[54]. The above factors and available social support are not independent from, but influence motivation, attitude and behavioral regulation.

Most of the PA barriers and facilitators emerged under the psychological/intrapersonal domain and were mostly OA-related. The data analysis allowed for new insights into the original studies, such as the emerging theme of OA-related distress and two distinct patterns in attitude, beliefs, motivation and behavioral regulation- one facilitating and the other hindering PA. Pain and its multifaceted impact is a source of distress in OA[24]. In turn, anxiety and depressive symptoms, which are more prevalent in people with arthritis[63], are predictors of poorer function[64 65] and pain[29 66-68]. Still cognitive processes underlying the distinct patterns are missing, e.g. what distinguishes those who for a given level of structural disease-severity and OA-related pain exhibit a positive attitude and behavioral regulation from those who are resigned, cope ineffectively with OA-stress and lack self-regulation. Explanations might lie in theoretical frameworks of behavior change and health,

For peer review only - http://bmjopen.bmj.com/site/about/guidelines.xhtml

which are absent in the included studies, with one exception[41]. For example, self-efficacy, self-determination and need satisfaction are precursors of behavior in PA motivation theories[69 70], whereas sense of control is a common concept in stress and coping literature[71]. Future research should make use of theoretical knowledge and approaches to enable targeted and more effective research and interventions[72].

The SR findings have strong implications for clinical practice. All healthcare professionals who deal with patients affected by lower-limb OA have a key role in facilitating PA through their advice, behavior and decisions. This should be taken into account in interactions with patients. Even without directed advice to increase PA, health and condition-related advice and a supportive stance from health professionals can influence decisions related to PA engagement[73]. In the absence of education, patients draw from lay and often fatalistic beliefs of PA in OA. An individual assessment of the experienced impact of pain and disability, personal attitudes and circumstances, educating patients about the role of PA in OA management, offering feasible yet specific PA prescription and encouragement can have an impact on the patient's PA behavior. Pain and stress-related coping strategies, guidance through exercise prescription and effective communication are main components of established arthritis self-management programs[74]. Increasing the time designated to each patient within the health care system could allow for such practices to take place. Counselling referral and online educational tools could also affect PA behaviors.

Based on the available qualitative evidence it was not possible to explore the secondary SR questions, an issue which has been previously reported [53 75]. Seven of the included studies focused on exercise regimes, including four on particular exercise interventions. Only one study made the distinction between PA uptake and maintenance. This is surprising considering the paradigm shift in health literature from exercise promotion to a combination of PA promotion and sedentary time reduction[76], as well as the existing work on motivational processes and the transtheoretical model[77 78]. The above domains need to be further explored and understood.

This SR has applied rigorous methods and provides an in-depth understanding of barriers and facilitators of PA in hip and knee OA based on the accumulated existing qualitative evidence, thus moving one step forward from existing SRs[21 22]. Gaps in the existing literature were also identified. With regards to data synthesis, coding participants' quotes and authors' interpretations separately allowed aspects of the phenomenon not captured by the original studies to come to light. During data synthesis peer review by a multidisciplinary team took place to enhance credibility. The main reviewer's background is clinical psychology, which might be reflected in the emphasis on the "psychological" component of PA barriers and facilitators.

There are certain limitations to this study. Although the term PA is used in the findings, in the original

For peer review only - http://bmjopen.bmj.com/site/about/guidelines.xhtml

quotes exercise is the term used most often. Due to resource and time limitations studies not written in English were excluded. Two relevant studies were also excluded because they were in a conference abstract form and additional data were not available[79 80]. Still the SR findings are consistent with existing literature and offer a meaningful understanding of the phenomenon of interest. Lastly, due to the nature of the evidence, directions of the relationships and interactions among the identified factors cannot be drawn.

There is a complex interplay among the physical, intrapersonal, psychological and socio-environmental barriers and facilitators of PA that bears similarities with other chronic diseases, but also includes characteristics specific to OA. Personal experiences, beliefs, attitudes and emotions, as well as the social environment, i.e. health care and social support, are dynamic factors shaping PA behavior. Considering that OA becomes more prevalent with age, it is important and challenging to make sustained lifestyle changes that will have a positive impact on an individual, as well as at health-care system level. With the aim of identifying effective practices to help people with OA become more active, future research should implement quantitative designs that take into account the factors identified above, as well as qualitative designs to address existing gaps in the literature.

Amendments to the protocol Please see supplement 4.

Competing interests No competing interests to declare.

Funding This review comprises part of the research requirements of a PhD to be completed by AMK, funded by the MRC-Arthritis Research UK Centre for Musculoskeletal Ageing Research.

Authors' contributions Study concept and design: JLD, AR, AMK, RK, AbA, NE. Searches: AMK, AsA. Study appraisal: AMK, NE. Data analysis: AMK, checked by NE. Data interpretation: AMK, checked by JLD, AR, NE. Manuscript draft: AMK. Manuscript review and input: JLD, AR, AbA, NE, RK. All authors provided feedback and approved the final draft.

Data sharing: Qualitative synthesis level electronic data (NVivo 11) are available upon request from the corresponding author.

For peer review only

References

1. NICE. Osteoarthritis care and management in adults. NICE clinical guideline 177 2014.
2. McAlindon TE, Bannuru RR, Sullivan MC, et al. OARSI guidelines for the non-surgical management of knee osteoarthritis. Osteoarthritis and cartilage / OARS, Osteoarthritis Research Society 2014;**22**(3):363-88.
3. Caspersen CJ, Powell KE, Christenson GM. Physical activity, exercise, and physical fitness: definitions and distinctions for health-related research. Public Health Reports 1985;**100**(2):126-31.
4. Fransen M, McConnell S, Harmer AR, et al. Exercise for osteoarthritis of the knee: a Cochrane systematic review. British Journal of Sports Medicine 2015;**49**(24):1554-7.
5. Fransen M, McConnell S, Hernandez-Molina G, et al. Exercise for osteoarthritis of the hip. Cochrane Database of Systematic Reviews 2014 2014(4).
6. Anwer S, Alghadir A, Brismee JM. Effect of Home Exercise Program in Patients With Knee Osteoarthritis: A Systematic Review and Meta-analysis. Journal of Geriatric Physical Therapy 2016;**39**(1):38-48.
7. Juhl C, Christensen R, Roos EM, et al. Impact of exercise type and dose on pain and disability in knee osteoarthritis: a systematic review and meta-regression analysis of randomized controlled trials. Arthritis & Rheumatology 2014;**66**(3):622-36.
8. Boesen MI, Boesen A, Koenig MJ, et al. Ultrasonographic investigation of the Achilles tendon in elite badminton players using color Doppler. American Journal of Sports Medicine 2006;**34**(12):2013-21.
9. Uthman OA, van der Windt DA, Jordan JL, et al. Exercise for lower limb osteoarthritis: systematic review incorporating trial sequential analysis and network meta-analysis.[Reprint of BMJ. 2013;347:f5555; PMID: 24055922]. British Journal of Sports Medicine 2014;**48**(21):1579.
10. Pisters MF, Veenhof C, van Meeteren NL, et al. Long-term effectiveness of exercise therapy in patients with osteoarthritis of the hip or knee: a systematic review. Arthritis & Rheumatism 2007;**57**(7):1245-53.
11. Pisters MF, Veenhof C, de Bakker DH, et al. Behavioural graded activity results in better exercise adherence and more physical activity than usual care in people with osteoarthritis: a cluster-randomised trial. Journal of Physiotherapy 2010;**56**(1):41-7.
12. Batsis JA, Germain CM, Vasquez E, et al. Physical Activity Predicts Higher Physical Function in Older Adults: The Osteoarthritis Initiative. J Phys Act Health 2016;**13**(1):6-16.
13. Chmelo E, Nicklas B, Davis C, et al. Physical activity and physical function in older adults with knee osteoarthritis. J Phys Act Health 2013;**10**(6):777-83.
14. Lin W, Alizai H, Joseph GB, et al. Physical activity in relation to knee cartilage T2 progression measured with 3 T MRI over a period of 4 years: data from the Osteoarthritis Initiative. Osteoarthritis and cartilage / OARS, Osteoarthritis Research Society 2013;**21**(10):1558-66.
15. Loprinzi PD, Sheffield J, Tyo BM, et al. Accelerometer-determined physical activity, mobility disability, and health. Disability and health journal 2014;**7**(4):419-25.
16. Austin S, Qu H, Shewchuk RM. Association between adherence to physical activity guidelines and health-related quality of life among individuals with physician-diagnosed arthritis. Quality of life research : an international journal of quality of life aspects of treatment, care and rehabilitation 2012;**21**(8):1347-57.
17. Austin J, Williams WR, Hutchison S. Multidisciplinary management of elderly patients with chronic heart failure: five year outcome measures in death and survivor groups. European Journal of Cardiovascular Nursing 2009;**8**(1):34-39 6p.

For peer review only - http://bmjopen.bmj.com/site/about/guidelines.xhtml

18. Herbolsheimer F, Schaap LA, Edwards MH, et al. Physical Activity Patterns Among Older Adults With and Without Knee Osteoarthritis in Six European Countries. Arthritis Care & Research 2016;**68**(2):228-36.
19. Marks R. Knee osteoarthritis and exercise adherence: a review. Current Aging Science 2012;**5**(1):72-83.
20. Marks R, Allegrante JP. Chronic osteoarthritis and adherence to exercise: a review of the literature. Journal of Aging & Physical Activity 2005;**13**(4):434-60.
21. Stubbs B, Hurley M, Smith T. What are the factors that influence physical activity participation in adults with knee and hip osteoarthritis? A systematic review of physical activity correlates. Clinical Rehabilitation 2015;**29**(1):80-94.
22. Veenhof C, Huisman PA, Barten JA, et al. Factors associated with physical activity in patients with osteoarthritis of the hip or knee: a systematic review. Osteoarthritis & Cartilage 2012;**20**(1):6-12.
23. Dobson F, Bennell KL, French SD, et al. Barriers and Facilitators to Exercise Participation in People with Hip and/or Knee Osteoarthritis: Synthesis of the Literature Using Behavior Change Theory. American journal of physical medicine & rehabilitation / Association of Academic Physiatrists 2016.
24. Hawker GA, Stewart L, French MR, et al. Understanding the pain experience in hip and knee osteoarthritis--an OARSI/OMERACT initiative. Osteoarthritis and cartilage / OARS, Osteoarthritis Research Society 2008;**16**(4):415-22.
25. Sanchez-Heran A, Agudo-Carmona D, Ferrer-Pena R, et al. Postural Stability in Osteoarthritis of the Knee and Hip: Analysis of Association With Pain Catastrophizing and Fear-Avoidance Beliefs. PM R 2016;**8**(7):618-28.
26. Holla JF, van der Leeden M, Knol DL, et al. Predictors and outcome of pain-related avoidance of activities in persons with early symptomatic knee osteoarthritis: a five-year followup study. Arthritis Care Res (Hoboken) 2015;**67**(1):48-57.
27. Holla JF, Sanchez-Ramirez DC, van der Leeden M, et al. The avoidance model in knee and hip osteoarthritis: a systematic review of the evidence. J Behav Med 2014;**37**(6):1226-41.
28. Herbolsheimer F, Schaap LA, Edwards MH, et al. Physical Activity Patterns Among Older Adults With and Without Knee Osteoarthritis in Six European Countries. Arthritis Care Res (Hoboken) 2016;**68**(2):228-36.
29. Somers TJ, Keefe FJ, Godiwala N, et al. Psychosocial factors and the pain experience of osteoarthritis patients: new findings and new directions. Curr Opin Rheumatol 2009;**21**(5):501-6.
30. Wideman TH, Finan PH, Edwards RR, et al. Increased sensitivity to physical activity among individuals with knee osteoarthritis: relation to pain outcomes, psychological factors, and responses to quantitative sensory testing. Pain 2014;**155**(4):703-11.
31. Arksey HOM, L. Scoping studies: towards a methodologica framework. International Journal of Social Research Methodology 2005:19-32.
32. Kanavaki AM, Rushton A, Klocke R, et al. Barriers and facilitators to physical activity in people with hip or knee osteoarthritis: protocol for a systematic review of qualitative evidence. BMJ Open 2016;**6**(11):e012049.
33. Altman RD. Criteria for classification of clinical osteoarthritis. The Journal of rheumatology Supplement 1991;**27**:10-2.
34. K H. Critical appraisal of qualitative research. In: Noyes J BA, Hannes K, Harden A, Harris J, Lewin S, Lockwood C ed. Supplementary Guidance for Inclusion of Qualitative Research in Cochrane Systematic Reviews of Interventions Version 1 (updated August 2011): Cochrane Collaboration Qualitative Methods Group, 2011.

For peer review only

35. Critical Appraisal Skills Programme (CASP). CASP Checklists (http://wwwcasp-uknet/#!checklists/cb36) Oxford: CASP, 2014.
36. Creswell JW. *Qualitative Inquiry and Research Design*. Thousand Oaks, California: SAGE Publications Inc., 2007.
37. Barnett-Page E, Thomas J. Methods for the synthesis of qualitative research: a critical review. BMC Med Res Methodol 2009;**9**:59.
38. Engel GL. The clinical application of the biopsychosocial model. Am J Psychiatry 1980;**137**(5):535-44.
39. Campbell R, Evans M, Tucker M, et al. Why don't patients do their exercises? Understanding non-compliance with physiotherapy in patients with osteoarthritis of the knee. Journal of Epidemiology and Community Health 2001;**55**(2):132-38.
40. Fisken AL, Waters DL, Hing WA, et al. Perceptions towards aqua-based exercise among older adults with osteoarthritis who have discontinued participation in this exercise mode. Australasian Journal on Ageing 2015.
41. Hammer NM, Bieler T, Beyer N, et al. The impact of self-efficacy on physical activity maintenance in patients with hip osteoarthritis – a mixed methods study. Disability and Rehabilitation 2015:1-14.
42. Hendry M, Williams NH, Markland D, et al. Why should we exercise when our knees hurt? A qualitative study of primary care patients with osteoarthritis of the knee. Family Practice 2006;**23**(5):558-67.
43. Kabel A, Dannecker EA, Shaffer VA, et al. Osteoarthritis and social embarrassment: Risk, Pain, And avoidance. SAGE Open 2014;**4**(2).
44. Kaptein SA, Backman CL, Badley EM, et al. Choosing where to put your energy: a qualitative analysis of the role of physical activity in the lives of working adults with arthritis. Arthritis care & research 2013;**65**(7):1070-6.
45. Petursdottir U, Arnadottir SA, Halldorsdottir S. Facilitators and Barriers to Exercising Among People With Osteoarthritis: A Phenomenological Study. Physical Therapy 2010;**90**(7):1014-25.
46. Stone RC, Baker J. Painful Choices: A Qualitative Exploration of Facilitators and Barriers to Active Lifestyles Among Adults With Osteoarthritis. J Appl Gerontol 2015.
47. Thorstensson CA, Roos EM, Petersson IF, et al. How do middle-aged patients conceive exercise as a form of treatment for knee osteoarthritis? Disability and Rehabilitation 2006;**28**(1):51-59.
48. Veenhof C, van Hasselt TJ, Koke AJA, et al. Active involvement and long-term goals influence long-term adherence to behavioural graded activity in patients with osteoarthritis: a qualitative study. Australian Journal of Physiotherapy 2006;**52**(4):273-78.
49. Biddle SJH, Mutrie, N. *Psychology of physical activity. Determinants, well-being & interventions*. 2nd ed. New York: Routledge, 2008.
50. Kwasnicka D, Dombrowski SU, White M, et al. Theoretical explanations for maintenance of behaviour change: a systematic review of behaviour theories. Health Psychol Rev 2016;**10**(3):277-96.
51. Franco MR, Tong A, Howard K, et al. Older people's perspectives on participation in physical activity: a systematic review and thematic synthesis of qualitative literature. Br J Sports Med 2015;**49**(19):1268-76.
52. Morgan F, Battersby A, Weightman AL, et al. Adherence to exercise referral schemes by participants - what do providers and commissioners need to know? A systematic review of barriers and facilitators. BMC Public Health 2016;**16**:227.

For peer review only

For peer review only - http://bmjopen.bmj.com/site/about/guidelines.xhtml

53. Rhodes RE, Quinlan A. Predictors of physical activity change among adults using observational designs. Sports Med 2015;**45**(3):423-41.
54. Gignac MA, Davis AM, Hawker G, et al. "What do you expect? You're just getting older": A comparison of perceived osteoarthritis-related and aging-related health experiences in middle- and older-age adults. Arthritis Rheum 2006;**55**(6):905-12.
55. Liu SH, Driban JB, Eaton CB, et al. Objectively Measured Physical Activity and Symptoms Change in Knee Osteoarthritis. Am J Med 2016;**129**(5):497-505 e1.
56. Horne M, Tierney S. What are the barriers and facilitators to exercise and physical activity uptake and adherence among South Asian older adults: a systematic review of qualitative studies. Prev Med 2012;**55**(4):276-84.
57. Wilcox S, Ananian CD, Abbott J, et al. Perceived exercise barriers, enablers, and benefits among exercising and nonexercising adults with arthritis: Results from a qualitative study. Arthritis & Rheumatism-Arthritis Care & Research 2006;**55**(4):616-27.
58. Peeters GM, Brown WJ, Burton NW. Psychosocial factors associated with increased physical activity in insufficiently active adults with arthritis. J Sci Med Sport 2015;**18**(5):558-64.
59. Main CJ, Williams AC. Musculoskeletal pain. BMJ 2002;**325**(7363):534-7.
60. Ross MM, Carswell A, Hing M, et al. Seniors' decision making about pain management. J Adv Nurs 2001;**35**(3):442-51.
61. NICE. Osteoarthritis care and management in adults. NICE clinical guideline, 2014.
62. Larmer PJ, Reay ND, Aubert ER, et al. Systematic review of guidelines for the physical management of osteoarthritis. Arch Phys Med Rehabil 2014;**95**(2):375-89.
63. He Y, Zhang M, Lin EH, et al. Mental disorders among persons with arthritis: results from the World Mental Health Surveys. Psychol Med 2008;**38**(11):1639-50.
64. Dekker J, Boot B, van der Woude LH, et al. Pain and disability in osteoarthritis: a review of biobehavioral mechanisms. J Behav Med 1992;**15**(2):189-214.
65. Sharma L, Cahue S, Song J, et al. Physical functioning over three years in knee osteoarthritis: role of psychosocial, local mechanical, and neuromuscular factors. Arthritis Rheum 2003;**48**(12):3359-70.
66. Edwards RR, Dworkin RH, Sullivan MD, et al. The Role of Psychosocial Processes in the Development and Maintenance of Chronic Pain. The journal of pain : official journal of the American Pain Society 2016;**17**(9 Suppl):T70-92.
67. de Rooij M, van der Leeden M, Avezaat E, et al. Development of comorbidity-adapted exercise protocols for patients with knee osteoarthritis. Clin Interv Aging 2014;**9**:829-42.
68. Volders S, Boddez Y, De Peuter S, et al. Avoidance behavior in chronic pain research: a cold case revisited. Behav Res Ther 2015;**64**:31-7.
69. Deci EL, Ryan RM. Self-determination theory: a macrotheory of human motivation, development, and health. Canadian Psychology 2008;**49**(3):182-85.
70. Bandura A. Self-efficacy: toward a unifying theory of behavioral change. Psychol Rev 1977;**84**(2):191-215.
71. Jensen MP, Turner JA, Romano JM, et al. Coping with chronic pain: a critical review of the literature. Pain 1991;**47**(3):249-83.
72. Michie S. Designing and implementing behaviour change interventions to improve population health. Journal of health services research & policy 2008;**13 Suppl 3**:64-9.
73. Horne M, Skelton D, Speed S, et al. The influence of primary health care professionals in encouraging exercise and physical activity uptake among White and South Asian older adults: experiences of young older adults. Patient Educ Couns 2010;**78**(1):97-103.

For peer review only - http://bmjopen.bmj.com/site/about/guidelines.xhtml

74. Lorig KR, Mazonson PD, Holman HR. Evidence suggesting that health education for self-management in patients with chronic arthritis has sustained health benefits while reducing health care costs. Arthritis Rheum 1993;**36**(4):439-46.
75. Koeneman M, Verheijden M, Chinapaw M, et al. Determinants of physical activity and exercise in healthy older adults: a systematic review. International Journal of Behavioral Nutrition & Physical Activity 2011;**8**.
76. Katzmarzyk PT. Physical Activity, Sedentary Behavior, and Health: Paradigm Paralysis or Paradigm Shift? Diabetes 2010;**59**(11):2717-25.
77. Prochaska JO, DiClemente CC. Stages and processes of self-change of smoking: toward an integrative model of change. J Consult Clin Psychol 1983;**51**(3):390-5.
78. Kinnafick FE, Thogersen-Ntoumani C, Duda JL. Physical activity adoption to adherence, lapse, and dropout: a self-determination theory perspective. Qual Health Res 2014;**24**(5):706-18.
79. Gay C EB, Levyckyj C, Chabaud A, Coudeyre E. Facilitators and Barriers in physical activity engagement for knee osteoarthritis patients. 30th Annual Congress of the French Society of Physical and Rehabilitation Medicine: Annals of Physical and Rehabilitation Medicine, 2015.
80. Jinks C, Moore A, Holden MA, et al. UPTAKE AND MAINTENANCE OF PHYSIOTHERAPY-LED EXERCISE AND GENERAL PHYSICAL ACTIVITY IN OLDER ADULTS WITH KNEE PAIN: THE BEEP LONGITUDINAL QUALITATIVE STUDY. Rheumatology 2015;**54**:36-36.

For peer review only

For peer review only - http://bmjopen.bmj.com/site/about/guidelines.xhtml

Table 1. Study characteristics

Study	Objectives	OA Site	Country	Participants (number, characteristics, sampling)	Methods (Data collection & analysis)	Findings	Relevance to Secondary Objectives (Exercise vs Lifestyle physical activity, Uptake vs Maintenance)
Campbell et al. (2001)	To explore compliance with physiotherapy, i.e. a home based exercise intervention.	Knee	UK	20 participants (8 had a follow up interview one year later); 14 female; all age groups above 45; 8 fully compliant till the end of the program, 12 partially compliant; maximum variation sample.	Interviews; constant comparative method	Initial compliance was due to a sense of moral oblication towards the physiotherapist. Continued compliance was linked to vieweing exercise as beneficial (precondition), ability and willingness to fit exercises in daily life, perceived symptom severity, arthritis and comodbidity attitudes, existing exercise and OA experiences.	Exercise regime (home based exercise).<br><br>Yes-Initial compliance to programme and one year follow up.
Fiskenn et al. (2015)	Reasons for ceasing participation in aqua based exercise.	Various	New Zealand	11 participants, female; over 60; recruitment through advertisements in health clinics and medical centres.	Focus groups; general inductive thematic approach.	Main reported barriers were lack of appropriate classes and knowledgeable instructors, which often led to an increase in pain, cold water and the facilities. Improvements in physical ability and the social component were the key reported benefits.	Exercise regime (aqua-based).<br><br>No distinction (non maintenance)
Hammer et al. (2015)	Exploring self-efficacy in relation to PA maintenance among maintainers and non-maintainers post-intervention (i.e. 4-month, 3-arm RCT: supervised, group strengthening exercise; supervised, group Nordic walking; unsupervised home exercise).	Hip	Denmark	15 participant (at 12 months f.u.); 8 female; mean age 66; community dweling; criterion based purposeful sampling.	Mixed methods. For the qualitative substudy: semi-structured interviews; directed content analysis.	Four pre-defined and one additional theme emerged: mastery experiences, vicarious experiences, verbal persuasion, physiological and emotional states, altruism.	Exercise regimes.<br><br>No distinction. Maintainers(if at 12 months reported changed PA behaviour) and non-maintainers.
Hendry et al. (2006)	Explore primary care patients' views towards exercise and factors that determine acceptability and motivation to exercise; identify barriers that limit its use.	Knee	UK	22 participants; 16 women; 52-86 years old; symptom duration 6months-25 years;mild to severe symptoms; purposive sampling; recruitment from GPs(20) and gyms(2).	Interviews and focus group; Principles of Framework method of qualitative analysis.	Three main categories: perception of physical capacity, beliefs about exercise (experience, advice, arthritis aetiology), motivational factors (enjoyment and social support, taking control of disability, priority setting, context). Exercise behaviour typology: long-term sedentary, long-term active, retired form exercise, converted to exercise.	Exercise (broad definition).<br><br>No distinction.
Kabel et al. (2014)	Role of pain, social pressure and embarrassment in activity related decision making.	Knee	Columbia, USA	10, community dwelling, mean age 60 years (SD=10.4), 7 women, 9 Caucasian	Interviews; Grounded theory or constant comparative method	Embarassment-related experiences regarding PA were linked both to engaging and avoiding activities. Each categorie included two subgoups: individuals avoiding pain and individuals avoiding embarrassment.	PA (living with OA)<br><br>No
Kaptein et	PA perception in the context	(OA &	Ontario,	40 (20 OA, hip/knee prevalent	Focus groups	PA perceptions were overall positive, yet PA,	PA

For peer review only

al. (2014)	of managing arthritis and multiple roles.	IA) various	Canada	group); recruitment from community and arthritis related clinics and groups	Qualitative content analysis	arthritis and life roles relationship is complex. PA as potential cause of arthritis, their reciprocal relationship, PA harms and benefits, perceived choices reg PA engagement and social support were the overarching themes that emerged in the discussions.	No distinction
Petursdottir et al. (2010)	Exercise experience. What determines whether people exercise	various	Iceland	12 (16 interviews), 9 women; purposeful sampling, minimum 5 years of OA and 50 years of age, urban area; outpatient clinics and targeted newsletter	Interviews; Phenomenology (Vancouver School)	Internal (individual attributes and exercise experiences) and external (social and physical environment) factors act as both barriers and facilitators in a delineated model.	Exercise<br><br>No distinction
Stone & Baker (2015)	Facilitators and barriers to regular PA	Hip or knee	Toronto, Canada	15, mean OA diagnosis 5 years; primary care and snowball sampling	Semi-structured interview; Interpretational analysis	Facilitators: pain relief, clear communication from health-care professionals, social support. Barriers: pain, psychological distress, lack of support from health care professionals	PA<br><br>No distinction
Thorstensson et al. (2006)	Underlying processes leading to response or non-response to exercise as treatment	Knee	Sweden	16, middle aged; purposefully chosen from the participants of an exercise intervention	Interviews; Phenomenography	Concerns about exercise as OA treatment. Four descriptive themes: to gain health, to become motivated, to experience the need for support, to experience resistance.	Exercise<br><br>No distinction
Veenhof et al. (2006)	Factors that explain differences between patients who integrated activities in their daily lives and those who didn't	Hip or knee	Netherlands	12, a subsample of participants from a behavioural graded activity intervention-deliberate sampling for heterogeneity (Patient Global assessment scores)	Interviews; Grounded theory	Lack of consistency in factors relating to adherence. Long-term goals at the beginning and active involvement in the intervention related to greater adherence.	Exercise<br><br>No distinction

For peer review only

Table 2. Study appraisal

		Campbell et al. (2001)	Fisken et al. (2015)	Hammer et al. (2015)	Hendry et al. (2006)	Kabel et al. (2014)	Kaptein et al. (2013)	Petursdottir et al. (2010)	Stone & Baker (2015)	Thorstensson et al. (2006)	Veenhof et al. (2006)
**CASP Qualitative Checklist**		6/10	6/10	6/10	9/10	6/10	7/10	9/10	9/10	7/10	6/10
1. Was there a clear statement of the aims of the research?		✓	✓	✓	✓	✓	✓	✓	✓	✓	✓
2. Is a qualitative methodology appropriate?		✓	✓	✓	✓	✓	✓	✓	✓	✓	?
3. Was the research design appropriate to address the aims of the research?		?	✓	✓	✓	?	x	✓	✓	?	?
4. Was the recruitment strategy appropriate to the aims of the research?		✓	?	✓	✓	✓	✓	✓	✓	?	✓
5. Was the data collected in a way that addressed the research issue?		✓	?	x	✓	?	✓	✓	✓	?	✓
6. Has the relationship between researcher and participants been adequately considered?		?	?	x	✓	?	x	✓	?	✓	?
7. Have ethical issues been taken into consideration?		?	✓	✓	?	✓	✓	?	✓	✓	✓
8. Was the data analysis sufficiently rigorous?		?	?	?	✓	?	?	✓	✓	✓	✓
9. Is there a clear statement of findings?		✓	✓	?	✓	✓	✓	✓	✓	✓	✓
10. How valuable is the research?		✓	✓	✓	✓	✓	✓	✓	✓	✓	?
**Trustworthiness**	**Credibility**	✓		✓	✓	✓	✓	✓	✓	✓	✓
	**Transferability**	✓	✓	✓	✓	✓	✓	✓	✓	✓	
	**Dependability**								✓	✓	
	**Confirmability**	✓	✓	✓	✓		✓	✓	✓	✓	✓

✓ *= yes,* x*= no,* ?*= can't tell*

Table 3. Barriers and facilitators: Themes, subthemes and number of supporting references

Domain	Major themes	**Barriers**	No of studies	No of ref.	**Facilitators**	No of studies	No of ref.
Physical health		Physical barriers and limitations (Pain and other symptoms; Perceived functional limitations)	9	94	PA for mobility, symptom relief and health (PA to maintain mobility; PA for symptom relief; PA for health)	9	34
Intrapersonal factors	Experience and beliefs about PA and OA	PA as non-effective, harmful or of doubtful effectiveness	6	36	PA as beneficial	7	60
		OA beliefs	5	17	Knowledge about PA	4	15
	Behavioural regulation & attitude	Resigned to OA	5	10	Keep going despite OA	7	18
		Lack of motivation	6	14	Adjustments, prioritisation and personal effort (Adjusting PAs; Prioritising PA; Personal responsibility and effort in being physically active)	9	41
		Lacking behavioural regulation	4	23			
	Emotions	OA-related distress	6	23	Enjoyment	4	22
Social Environment	Health professionals	Lack of advice and encouragement from health professionals	5	22	Support from health professionals	8	50
	Social support	Social comparison as demotivating	5	15	Social support facilitating PA	7	43
		Lack of social support	4	8			

For peer review only

Supplement 1. PRISMA checklist of items to include when reporting a systematic review or meta-analysis

Section/topic	#	Checklist item	Reported on page #
**TITLE**			
Title	1	Identify the report as a systematic review, meta-analysis, or both.	1
**ABSTRACT**			
Structured summary	2	Provide a structured summary including, as applicable: background; objectives; data sources; study eligibility criteria, participants, and interventions; study appraisal and synthesis methods; results; limitations; conclusions and implications of key findings; systematic review registration number.	1
**INTRODUCTION**			
Rationale	3	Describe the rationale for the review in the context of what is already known.	2
Objectives	4	Provide an explicit statement of questions being addressed with reference to participants, interventions, comparisons, outcomes, and study design (PICOS).	2
**METHODS**			
Protocol and registration	5	Indicate if a review protocol exists, if and where it can be accessed (e.g., Web address), and, if available, provide registration information including registration number.	2
Eligibility criteria	6	Specify study characteristics (e.g., PICOS, length of follow-up) and report characteristics (e.g., years considered, language, publication status) used as criteria for eligibility, giving rationale.	2
Information sources	7	Describe all information sources (e.g., databases with dates of coverage, contact with study authors to identify additional studies) in the search and date last searched.	3
Search	8	Present full electronic search strategy for at least one database, including any limits used, such that it could be repeated.	3
Study selection	9	State the process for selecting studies (i.e., screening, eligibility, included in systematic review, and, if applicable, included in the meta-analysis).	3

For peer review only

Section/topic	#	Checklist item	Reported on page #
Data collection process	10	Describe method of data extraction from reports (e.g., piloted forms, independently, in duplicate) and any processes for obtaining and confirming data from investigators.	3
Data items	11	List and define all variables for which data were sought (e.g., PICOS, funding sources) and any assumptions and simplifications made.	3
Risk of bias in individual studies	12	Describe methods used for assessing risk of bias of individual studies (including specification of whether this was done at the study or outcome level), and how this information is to be used in any data synthesis.	n/a, 2.8 Quality appraisal is reported as relevant to a qualitative SR #4
Summary measures	13	State the principal summary measures (e.g., risk ratio, difference in means).	n/a, 2.9 Phenomenon of interest is stated as relevant to a qualitative SR #4
Synthesis of results	14	Describe the methods of handling data and combining results of studies, if done, including measures of consistency (e.g., I^2) for each meta-analysis.	4
Risk of bias across studies	15	Specify any assessment of risk of bias that may affect the cumulative evidence (e.g., publication bias, selective reporting within studies).	n/a, further details are reported under section Amendments to the SR protocol #11 and supplement 4
Additional analyses	16	Describe methods of additional analyses (e.g., sensitivity or subgroup analyses, meta-regression), if done, indicating which were pre-specified.	4
**RESULTS**			
Study selection	17	Give numbers of studies screened, assessed for eligibility, and included in the	5

For peer review only

Section/topic	#	Checklist item	Reported on page #
		review, with reasons for exclusions at each stage, ideally with a flow diagram.	
Study characteristics	18	For each study, present characteristics for which data were extracted (e.g., study size, PICOS, follow-up period) and provide the citations.	5
Risk of bias within studies	19	Present data on risk of bias of each study and, if available, any outcome-level assessment (see Item 12).	n/a, 3.3 Study appraisal is reported, #5
Results of individual studies	20	For all outcomes considered (benefits or harms), present, for each study: (a) simple summary data for each intervention group and (b) effect estimates and confidence intervals, ideally with a forest plot.	Table 1
Synthesis of results	21	Present results of each meta-analysis done, including confidence intervals and measures of consistency.	5
Risk of bias across studies	22	Present results of any assessment of risk of bias across studies (see Item 15).	n/a
Additional analysis	23	Give results of additional analyses, if done (e.g., sensitivity or subgroup analyses, meta-regression [see Item 16]).	n/a
**DISCUSSION**			
Summary of evidence	24	Summarize the main findings including the strength of evidence for each main outcome; consider their relevance to key groups (e.g., health care providers, users, and policy makers).	8
Limitations	25	Discuss limitations at study and outcome level (e.g., risk of bias), and at review level (e.g., incomplete retrieval of identified research, reporting bias).	10
Conclusions	26	Provide a general interpretation of the results in the context of other evidence, and implications for future research.	11
**FUNDING**			
Funding	27	Describe sources of funding for the systematic review and other support (e.g., supply of data); role of funders for the systematic review.	11

For peer review only

Supplement 2. Search strategy for Medline

Draft MEDLINE search- Ovid interface

1	osteoarthritis.mp. or exp Osteoarthritis, Hip/ or exp Osteoarthritis/ or exp Osteoarthritis, Knee/
2	(osteoarthriti* or osteo-arthriti* or osteoarthrotic or osteoarthros*).ti,ab.
3	(coxarthrosis or gonarthrosis).ti,ab.
4	"knee pain".mp.
5	"hip pain".mp.
6	"lower limb".mp.
7	exp Lower Extremity/ or "lower extremit*".mp.
8	(degenerative adj2 arthritis).ti,ab.
9	1 or 2 or 3 or 4 or 5 or 6 or 7 or 8
10	physical activity.mp. or exp Motor Activity/
11	exp Exercise/ or exp Exercise Therapy/ or exercise.mp.
12	exp Sports/ or sports.mp.
13	exp Life Style/ or exp Sedentary Lifestyle/ or sedentary.mp.
14	"non-exercis*".ti,ab.
15	"activities of daily living".mp. or exp "Activities of Daily Living"/
16	10 or 11 or 12 or 13 or 14 or 15
17	(maintain* or maintenance or support* or ongoing or "on-going" or adherence or reinforc* or comply* or compliance or "long-term" or adoption or engagement or avoidance or boost* or refresh* or remind* or promotion or promot* or "physical activity uptake" or "behavio* change" or "lifestyle change").ti,ab.
18	(barrier* or impediment or limit* or facilitator* or enablers or enabl* or motivators or motivat* or influenc* or factors or determinants).ti,ab.
19	facilitator*.mp.

For peer review only

20 barrier*.mp.

21 adherence.mp.

22 exp Motivation/ or motivators.mp.

23 social support.mp. or exp Social Support/

24 17 or 18 or 19 or 20 or 21 or 22 or 23

25 exp Qualitative Research/ or qualitative.mp.

26 (interview* or theme* or experience).mp. [mp=title, abstract, original title, name of substance word, subject heading word, keyword heading word, protocol supplementary concept word, rare disease supplementary concept word, unique identifier]

27 ("content analysis" or "grounded theory" or "thematic analysis" or "phenomenological analysis" or phenomenolog* or narrative* or discourse or ethnograph*).ti,ab.

28 (("semi-structured" or semistructured or unstructured or informal or "in-depth" or indepth or "face-to-face" or structured or guide) adj3 (interview* or discussion* or questionnaire*)).ti,ab.

29 (focus group* or interview* or fieldwork or "field work" or triangulation or "data saturation" or "key informant").ti,ab.

30 25 or 26 or 27 or 28 or 29

31 9 and 16 and 24 and 30

For peer review only

Supplement 3. Findings: themes and supporting references.

1. Physical health

Barriers (#studies, #references)	References	Facilitators (#studies, #references)	References
Physical barriers and limitations (9, 94)	*While pain was not attributed to their participation in the intervention the pain was described as having a major impact on their perceived opportunity to be physically active at present.* (Hammer, 2015)<br>*All participants discussed experiencing intense physical pain on a daily basis, and how it negatively affected their desire to be active… In addition to these limitations, participants spoke of fatiguing rapidly, which made considering physical activity as more of a challenge* (Stone & Baker, 2015)<br>*Stiffness and fatigue were barriers to exercising. "It was like my body was made of lead"* (Petursdottir, 2010)<br>*Two participants, who were both hikers, reported limiting effects of OA knee pain… A grandmother shared fears and concerns regarding dropping or falling on her grandchildren due to both hand and knee pain.* (Kabel, 2014)<br>*'But as soon as someone says 'let's go for a walk….' It's the last thing I want to do because it hurts too much …'* (Kaptein, 2013)<br>*'…the day after I just couldn't cope, I was in so much pain'* (Fisken, 2015)<br>*'Exercise hurts. The pain was almost unbearable but I still carried on. Yes, it was very strenuous, but that's how it is, the pain becomes increasingly worse, I think…it just becomes more and more painful.'* (Thorstenson, 2006)<br>*Vi, Hilary, Ethel and Eileen all mentioned their being overweight as contributing to their knee symptom.* (Campbell, 2001)<br>*Ability was also limited by a perceived general lack of physical fitness, sometimes attributed to old age.* (Hendry, 2006)	PA for mobility, symptom relief and health (9, 34)	*Some informants even expressed how their PA maintenance was partly motivated by the belief that PA could help them to postpone or maybe avoid surgery* (Hammer et al., 2015).<br>*"The main motivation to do all this is to prevent an operation to get a new hip" (participant with long-term goal)* (Veenhof et al., 2006)<br>*"I realised my mobility would get worse if I didn't do something about it so I started exercising". (2, 3, 20, 25)* (Hendry et al., 2006)<br>*"I feel like the Tin Man- that if I stop moving, I'll rust up and that will be it"* (Kaptein et al., 2013)<br>*As with the pain, however, the experience of less stiffness and more stamina turned out to be facilitating.* (Petursdottir et al., 2010)<br>*"The physiotherapist professionally guided me to feel less pain. It made me want to do exercises on my own."* (Stone & Baker et al., 2015)<br>*The perceived severity of knee symptoms was an important factor in motivation, with those experiencing severe pain and/or loss of mobility being most likely to continue to exercise.* (Campbell et al., 2001)<br>*…hip pain was highlighted as a common symptom, and several informants linked a perceived reduction in pain to their increased PA level, which represented an important incentive to maintain PA post-intervention.* (Hammer et al., 2015)<br>"Well, it is different now because, as I've already said, previously you exercised to maintain your level of fitness whereas now you exercise in order to regain your physical condition…" (Thorstenson et al., 2006)<br>"*Strenghtening your muscles..keeping your weight down…keeps you in shape*" (Fisken et al., 2015)<br>Disconfirming case: *Some participants who scored high on the Patient Global Assessment (eg, because they perceived less pain) did not continue with their activities, while some participants who scored low on the Patient Global Assessment (eg, because their pain remained the same) reported that their level of activities had increased considerably.* (Veenhof et al., 2006)

For peer review only

2. Intrapersonal factors: themes and references.

Major theme	Barriers (#studies, #references)	References	Facilitators (#studies, #references)	References
Experience and beliefs about PA and OA	PA as non-effective, harmful or of doubtful effectiveness (6, 36)	*Experiencing pain while exercising made it difficult to decide whether it was beneficial or counterproductive.* (Thorstenson, 2006)<br>*[Counter-advice or no recommendations] created further confusion about physical activity and the potential benefits for osteoarthritis.* (Stone & Baker, 2015)<br>*Many participants were worried that exercise was wearing out their joints...* (Hendry, 2006)<br>*..many participants* [were] *uncertain whether PA was good or bad for them when they have arthritis.* (Kaptein, 2013).<br>*If, however, the benefits of the physiotherapy were not perceived as sufficient…non-compliance was a rational outcome…* (Campbell, 2001)<br>*The two informants who had not managed to maintain an increased level of PA expressed how they had hoped for an improvement in their hip specific symptoms, which none of them had achieved.* (Hammer, 2015)	PA as beneficial (7, 60)	*They continued to undertake exercises… from which they perceived they would derive the most benefit.* (Campbell, 2001)<br>*"Keeps the body moving, takes your mind off it, it's good to be outside. Yea, keeping active, or else if you've got osteo, it can get you right down…"* (Fisken, 2015)<br>[Among maintainers] *it was generally described how PA, in addition to the physical effect, also significantly contributed to their psychological well-being.* (Hammer, 2015)<br>*They* [maintainers] *were more likely to have noticed beneficial effects on their OA knee, or general health and well-being as a result of exercise.* (Hendry, 2006)<br>*Other participants were motivated by the results of the exercise, not because they liked it or enjoyed it.* (Petursdottir, 2010)<br>*The informants expressed satisfaction and were convinced of the effectiveness of exercise.* (Thorstenson, 2006)<br>*"I really know these exercises have beneficial effects and that motivates me to continue with my exercises"* (Veenhof, 2006)
	OA beliefs (5, 17)	*"There's no cure, only pain relief"* (Hendry, 2006)<br>*"There is nothing that can be done about OA; therefore, I do nothing"* (Petursdottir, 2010)<br>*"...exercise can help, I am convinced about that, although it did not work for me…If one had started to exercise five or six years earlier, it might have helped."* (Thorstenson, 2006)<br>Another said, *"… if you're a very active person, especially professional athletes … they basically tell you you'll have arthritis when you get older."* (Kaptein, 2013)<br>*"I was having trouble with my knees every so often it did hurt you know with one thing and another. Working in the construction industry there is a lot of lifting and a lot kneeling you see and I felt well I wonder if that's got anything to do with it."* (Campbell, 2001)	Knowledge about PA (4, 15)	*It was described how increased knowledge and information about PA had led to an increased awareness of exercising and of doing this at a certain intensity and frequency…* (Hammer, 2015)<br>*Most of the participants had experienced being educated by their physical therapists.* (Petursdottir 2010)<br>*..many* [participants] *were unaware of specific osteoarthritis-related benefits and unsure of what activities would provide optimal self-management.* (Stone & Baker, 2015)<br>*Overall, most informants understood and acknowledged, but many undertook only a limited programme of exercise.* (Campbell, 2002)<br>[To experience coherence] *This conception contained statements about connecting knowledge about osteoarthritis with knowledge and experiences of exercise.* (Thorstenson, 2006)<br>Disconfirming case: *"You are in a vicious circle where you become less and less active, and the bones are grinding more and more due to muscle weakness. I could see that and I could understand it, but the knowledge has not helped me"* (Hammer, 2015)

For peer review only

Behavioural regulation and attitude	Resigned to OA (5, 10)	Those who thought that arthritis was caused by immutable factors such as age, obesity and "wear and tear", tended to have a resigned attitude towards their arthritis. (Campbell, 2001)<br>...others had become resigned to their physical limitations… "I've accepted my limitations and said goodbye to going out." (Hendry, 2006)<br>"There is nothing that can be done about the OA; therefore, I do nothing" (Petursdottir, 2010)<br>...osteoarthritis-related pain can lead to disabling thoughts, which are precursors for adopting passive coping and learned helplessness. (Stone & Baker, 2015)<br>"… If one had started to exercise five or six years earlier, it might have helped." (Thorstenson, 2006)	Keep going despite OA (7, 18)	...those most likely to be continued compliers tended to believe that although there was no cure for arthritis, there were things they could do to minimise its impact, including the physiotherapy (Campbell, 2002)<br>Some participants were determined to take control of their disability and used exercise as a means of actively maintaining or improving their mobility. "I'm determined not to let my knee problem stop me from doing the things I want to do." (Hendry, 2006)<br>[To be prepared to persevere…] "I played 18 holes of golf and that is also quality of life. I refuse to sit a t home and navel gaze, I just won't" (Thorstenson, 2006)<br>"I worked out new ways to cope, to keep my arthritis from getting in the way too much"… They described the importance of not letting the OA control their lives, although its existence should be recognized and respected. (Petursdottir, 2010).<br>It appeared that all adherent participants were initially motivated to reach long-term goals. (Veenhof, 2006)<br>One participant shared that she continued to be physically active in her community, although she was concerned that others perceived her as being far older than her chronological age. (Kabel, 2014).<br>Occasionally participants mentioned adding new activities to their lives: "I learned how to ski about eight years ago. I always wanted to do it and I thought I'm not going to let this get me down" (Kaptein, 2013)
	Lack of motivation (6, 14)	"I suppose if there was a really good reason I would [be strongly disciplined]." (Campbell, 2001)<br>[The two non-maintainers also described obstacles for post-intervention PA…] the other described feeling a lack of motivation towards PA. (Hammer, 2015)<br>[Reasons for not finding time to exercise…] others freely admitted to being lazy or lacking motivation. (Hendry, 2006)<br>One of the participants seemed to lack the motivation to exercise, based on an overwhelming experience of boredom while exercising. She declared that she would never, ever exercise, no matter what. "It is dead boring, so I just don't do it and never will" (Petursdottir, 2010)<br>"You need to have the will to do it… when you are well you don't do it, and when you need to do it, then it hurts and therefore you don't do it (laughter)." (Thorstenson, 2006)<br>…all non-adherent participants reported a short-term initial goal or had no specific goal. (Veenhof, 2006)	Adjustments, prioritization and personal effort (9, 41)	The majority of informants described how they regularly adjusted their exercises and intensity in an attempt to strike a balance between continuously increasing intensity while at the same time considering the experienced pain. (Hammer, 2015)<br>They were eager to find activities and exercise that fitted them and, in many cases, adapted their exercises to their life with OA. (Petursdottir, 2010).<br>'My knees were getting really bad and I, so thought, well the only thing I can do really is to do aqua, which I did and I love it' (Fisken, 2015).<br>Prioritising exercise and making it part of a weekly routine helped some people to maintain their exercise habit. "… I try and say, OK well I'll go there [gym], have a shower and go shopping… I try to fit it in." (Hendry, 2006)<br>More important [in increasing motivation] was the willingness and ability to accommodate the exercises into everyday life. (Campbell, 2001)<br>"I continue with my exercises, they are integrated in my daily living." (Veenhof, 2006)<br>In order to deal with limited time and energy, many participants made tradeoffs. I've had to choose … where I put my energy, and I know that some days I feel that all I've done is work, so that's kind of a bummer" (Kaptein, 2013)<br>He engaged in modified activity, not playing as aggressively as he wanted to, to avoid pain but did not opt out of the activity completely. (Kabel, 2014)<br>"Well I suppose to some extent it is up to yourself how much effort you wish to put into it, … if I don't want to do anything then I don't think I ll benefit from any treatment. I suppose that at the end of the day the outcome of the treatment depends on no one but myself" (Thorstenson, 2006)<br>Disconfirming case: Later in their interviews both went on to admit some personal responsibility for their lack of compliance… "It's just excuses when it comes down to basics. I mean you know you could get up in the morning and do it between 6 or 7 or something like that." (Cambpell, 2001)
	Lacking behavioural regulation (4, 23)	Those who ceased exercising often cited conflict with regular routines to explain why continuing with exercises was not possible. (Campbell, 2001)<br>For others finding time to exercise was a low priority… "when I'm busy I forget." (Hendry 2006)<br>Despite recognising the importance of PA, it was considered optional or discretionary compared to essential roles such as work and family.(Kaptein, 2013)<br>"One is so occupied that it is very easy not to find time for exercise. Everything else takes precedence." (Thorstenson, 2006)		

For peer review only

Emotions	OA-related distress (6, 23)	*6 of the 10 participants self-identified as having some type of embarrassment-related experience, usually general embarrassment and frustration over their physical limitations due to the OA pain.* (Kabel, 2014)<br>*Participants expressed depressing thoughts, referring to osteoarthritis as "mentally agonizing".* (Stone & Baker, 2015)<br>*"I don't know if you can imagine how it is to be confronted with things that you want to do but you are unable to. That is mentally stressful."* (Hammer, 2015)<br>*A few of the women mentioned 'paralyzing fatigue' as a major barrier for getting anything done and felt it might be related more to mental fatigue...* (Petursdottir, 2010)<br>*A few individuals noted a loss of their identity as an athletic or physically active person… they often tried to hide difficulties with activities from others.* (Kaptein, 2013)<br>*"It got worse and worse and I started falling down ... it's so embarrassing."* (Campbell, 2001)	Enjoyment (4, 22)	*Not surprisingly, people who enjoyed exercising were likely to continue; those that disliked it stopped. "I really do enjoy the gym; I look forward to going."* (Hendry, 2006)<br>*Some participants based their motivation on the fact that they liked PA and therefore had been physically active. "I have always enjoyed physical activity"* (Petursdottir, 2010)<br>*"The buoyancy…I like deep water…It takes the impact off your joints…it gives you freedom…if you've been sedentary and not able to move around…the water makes you feel wonderful"'* (Fisken, 2015).<br>*"I feel such a fool standing on one leg and going up and down on my own and I tends to drop it I do."* [non-maintainer] (Campbell, 2001)

3a. Social Environment: themes and references.

Major theme	Barriers (#studies, #references)	References	Facilitators (#studies, #references)	References
Health professionals	Lack of support from health professionals (5, 22)	*Sometimes the advice was vague or absent… Occasionally exercise was discouraged.* (Hendry et al., 2006)<br>*"So I go to the doctor and all he just simply done was put his hand on my knee, he said 'move your leg,…you are getting old, you've got rheumatism.'"* (Campbell et al., 2001)<br>*…physicians often provided them with counter advice or did not offer any recommendations…* (Stone & Baker, 2015)<br>*"They have not done it [encouraged exercising]"* (Petursdottir et al., 2010)<br>*"The instructor was not geared up for my particular disability [OA]… and I found it very stressful"* (Fisken et al., 2015)	Support from health professionals (8, 50)	*Advice from health professionals was mainly in favour of exercise and consisted of encouragement to exercise, advice about specific exercises, and referral to a gym. (Hendry et al., 2006)*<br>*The supervision by physical therapists highly influenced the informants' ability to progress in training intensity as the physical therapists verbally expressed their confidence in the participants and exhibited realistic expectations about their exercise abilities* (Hammer et al., 2015)<br>*All participants spoke about the instrumental role of health care providers in influencing and encouraging physical activity.* (Stone & Baker, 2015)<br>*"Well, I always say that my physical therapist is as good as any psychologist."* (Petursdottir et al., 2010)<br>*Overall, most informants understood and acknowledged, as they were instructed by the physiotherapist, that they should do the exercises often and regularly, but many undertook only a limited programme of exercise.* (Campbell et al., 2001)<br>*It appeared that all adherent participants reported that… the physiotherapists had a coaching role during intervention.* (Veenhof et al., 2006)<br>*"I think that [an instructor] is good because then you learn what to do so that you do not do it in the wrong way."* (Thorstenson et al., 2006)<br>*"…knowing that aqua is for people possibly who have arthritis…they ought to have.. an extra training course or something to fit, to accommodate that"* (Fisken et al., 2015)
Social support	Social comparison as demotivating (5, 15)	*Comparison with others with more limiting disease or a stoic attitude to knee symptoms all seemed to be associated with an attenuation of the motivation to comply"* (Campbell et al., 2001)<br>*"I found it very stressful to be honest because I felt like I had to do the same as the others and keep up…"*(Fisken et al., 2015).<br>"*They don't want to be dragged down by somebody that's not up to their standard I would think."* (Hendry et al., 2006)<br>*"I couldn't keep up with everyone else and felt like I was dragging them*	Social support facilitating PA (7, 43)	*The majority of informants described how they continued to exercise with others because of the mutual support and encouragement they hereby achieved…* (Hammer, 2015)<br>*The support, caring, and encouragement of others were among important external factors influencing how much the participants exercised.* (Petursdottir, 2010)<br>"*I think it's important to be with other people, how other people cope and that you're not alone and there are other people you know, in similar situations."* (Fisken, 2015).<br>*"I like the gym referral scheme because you're in a group of people who all have*

		behind." (Kabel et al., 2014)<br>Disconfirming case: *Participants also gave examples of persisting with a painful activity and risking intensifying the pain because of social pressure or the desire to avoid embarrassment and disapproval...* (Kabel et al., 2014).		*problems."* (Hendry, 2006)<br>*Eileen explained how difficult it was to continue the exercises programme since she stopped seeing the physiotherapist.* (Campbell, 2001)<br>*An important facilitator of PA and a strategy that helped some participants 'stay in the game' was having social support…* (Kaptein, 2013)<br>*"One of my friends who knows about my arthritis asked me if I ever exercise…Then she said she would work out with me if I wanted to. That was the first time I ever seriously thought about exercising.* (Stone & Baker, 2015)
	Lack of social support (4, 8)	[Sedentary informants] *had been given scant encouragement to exercise.* (Hendry et al., 2006) [Regarding family's attitudes] *some of the women expressed having a hard time justifying to themselves and their families their need to spend time exercising.* (Petursdottir et al., 2010).<br>*Not only* [about half of the participants] *did not receive support from others to manage physically demanding activities at work, they often tried to hide difficulties with activities from others.* (Kaptein et al., 2013)<br>*"If perhaps my wife would work with me and you had a bit of competition…"* (Campbell et al., 2001)		

Supplement 4. Amendments to the SR protocol

Confidence in the synthesised findings was not used due to ambiguities in the suggested process (ConQual[84]), i.e. regarding transparency and satisfactory justification of the assessment outcome. Alternatively, the number of sources and a high number of original references supporting each finding are reported in addition to study appraisal.

Kappa statistic was not measured. The two researchers run the searches independently for all databases following the Medline search strategy. Because of differences in operators and options at different search engines, the number of studies differed at the stages preceding study selection. Each reviewer's full text selection stage was updated by the other researcher's findings. At this stage agreement was met for all included studies.

For peer review only

BMJ Open

Barriers and facilitators of physical activity in knee and hip osteoarthritis: a systematic review of qualitative evidence

Journal:	*BMJ Open*
Manuscript ID	bmjopen-2017-017042.R1
Article Type:	Research
Date Submitted by the Author:	12-Jul-2017
Complete List of Authors:	Kanavaki, Archontissa; University of Birmingham, School of Sport, Exercise and Rehabilitation Sciences<br>Rushton, Alison; University of Birmingham<br>Efstathiou, Nikolaos ; University of Birmingham, Nursing, School of Medicine<br>Alrushud, Asma; Unoversity of Birmingham<br>Klocke, Rainer; 2 Dudley Group NHS Foundation Trust, Department of Rheumatology<br>Abhishek, A; University of Nottingham<br>Duda, Joan; University of Birmingham, Sport, Exercise & Rehabilitation Sciences
**Primary Subject Heading**:	Sports and exercise medicine
Secondary Subject Heading:	Rheumatology
Keywords:	osteoarthritis, systematic review, barriers, facilitators, physical activity

SCHOLARONE™
Manuscripts

Barriers and facilitators of physical activity in knee and hip osteoarthritis. A systematic review of qualitative evidence

Archontissa M. Kanavaki[1], Alison Rushton[1,2], Nikolaos Efstathiou[3], Asma Alrushud[1], Rainer Klocke[4], Abhishek Abhishek[5], Joan L. Duda[1]
[1]School of Sport, Exercise and Rehabilitation Sciences, College of Life and Environmental Sciences, University of Birmingham and MRC-Arthritis Research UK Centre for Musculoskeletal Ageing Research, University of Birmingham, Birmingham, UK, B152TT
[2]Centre of Precision Rehabilitation for Spinal Pain (CPR Spine), University of Birmingham, Birmingham, UK
[3]Nursing, Institute of Clinical Sciences, Medical School, University of Birmingham, Birmingham, UK, B152TT
[4]Department of Rheumatology, Dudley Group NHS Foundation Trust, Dudley, UK, DY1 2HQ
[5]Academic Rheumatology Unit, School of Medicine, Faculty of Medicine and Health Sciences, University of Nottingham, Nottingham, UK, NG7 2RD
Corresponding author: Archontissa M. Kanavaki, School of Sport, Exercise and Rehabilitation Sciences, University of Birmingham, Edgbaston, Birmingham, B15 2TT, UK. Email: amk377@bham.ac.uk

ABSTRACT

Physical activity (PA) including engagement in structured exercise, has a key role in the management of hip and knee osteoarthritis (OA), however maintaining a physically active lifestyle is a challenge for people with OA. PA determinants in this population need to be understood better so that they can be optimised by public health or healthcare interventions and social policy changes.

Objectives: To conduct a systematic review of the existing qualitative evidence on barriers and facilitators of PA for patients with hip or knee OA. Secondary objectives, to explore differences in barriers and facilitators between (i)lifestyle PA and exercise; (ii)PA uptake and maintenance.

Methods: MEDLINE, EMBASE, Web of Science, CINAHL, SPORTDiscus, Scopus, Grey literature and qualitative journals were searched. CASP-Qualitative checklist and Lincoln &Guba's criteria were used for quality appraisal. Thematic synthesis was applied.

Findings: Ten studies were included, seven focusing on exercise regimes, three on overall PA. The findings showed a good fit with the biopsychosocial model of health. Aiming at symptom relief and mobility, positive exercise experiences and beliefs, knowledge, a "keep going" attitude, adjusting and prioritising PA, having health-care professionals' and social support emerged as PA facilitators. Pain and physical limitations; non-positive PA experiences, beliefs and information; OA-related distress; a resigned attitude; lack of motivation, behavioural regulation, professional support; and negative social comparison with co-exercisers were PA barriers. All themes were supported by high and medium quality studies. Paucity of data did not allow for the secondary objectives to be explored.

Conclusion: Our findings reveal a complex interplay among physical, personal including psychological, and social-environmental factors corresponding to the facilitation and hindrance of PA, particularly exercise, engagement. Further research on the efficacy of individualized patient education, psychological interventions, or social policy change to promote exercise engagement and lifestyle PA in individuals with lower limb OA is required.

Trial registration number CRD42016030024

Keywords: osteoarthritis, physical activity, systematic review, barriers, facilitators

Word count: 4.078

Strengths and limitations

- This systematic review is the first to identify, appraise and synthesise the existing qualitative research on barriers and facilitators to PA in knee and hip osteoarthritis.
- Rigorous methods have been applied, informed by the Centre for Reviews and Dissemination and Cochrane Qualitative Research Methods Group guidelines and reported according to the Preferred Reporting Items for Systematic Reviews and Meta-Analyses (PRISMA) and Enhancing Transparency in Reporting the Synthesis of Qualitative Research (ENTREQ) statements.
- The majority of the included studies (7/10) focused on exercise barriers and facilitators, therefore barriers and facilitators of more general lifestyle PA might not be fully captured.
- Papers written in English language only were included.

Osteoarthritis (OA) is the commonest joint disease and main cause of disability in older adults[1]. OA management focuses on analgesia and non-pharmacological modalities such as exercise and weight loss[2]. Exercise, i.e. structured and purposeful physical activity (PA)[3], reduces pain and improves function in people with knee or hip OA[4-9]. However, despite the positive effects on symptoms, exercise interventions do not promote sustained behavior change [10 11]. Just like exercise, PA associates with better physical function[12-14], and even modest increase in PA (from sedentary to light intensity PA) improves arthritis pain[15]. At the population level, it is simpler to promote PA in people with painful OA e.g. via radio and television, than promoting exercise as that will require a greater behavior change and may need continued support of trained physiotherapists. However, existing evidence suggests that people with lower limb OA have such low PA levels that they gain no health benefits from it[16-18]. Thus, there is need to understand the determinants of reduced PA in people with symptomatic OA so that these can be optimised to promote PA.

The disease specific determinants of PA in those with lower limb OA e.g. symptom severity, physical function [19-23] are relatively well understood, but the psychological, social and environmental determinants of PA in OA have not been adequately examined[21 22]. Understanding these factors is of great importance as pain makes PA an aversive experience leading to activity avoidance[24-27] and pain is influenced by psychological and environmental factors[25 28-30]. A recent scoping review identified several psychological and environmental barriers and facilitators of exercise in people with hip or knee OA[23]. However, scoping reviews lack the methodological rigor of SRs[31]. A SR of qualitative data holds promise for a thorough and in-depth understanding of the modifiable psychosocial factors predicting PA behaviour.

For peer review only - http://bmjopen.bmj.com/site/about/guidelines.xhtml
For peer review only

The objectives of this study were to identify, appraise and synthesise the existing qualitative evidence on barriers and facilitators to PA in hip or knee OA, and explore differences in barriers and facilitators between lifestyle PA accrued in daily activities, and those reported in regard to structured exercise programs specifically; and between PA uptake and maintenance.

METHODS

This SR was registered with the International Prospective Register of SRs (CRD42016030024) and its protocol reported previously[32]. The reporting follows the Preferred Reporting Items for SRs and Meta-Analyses (PRISMA) and the Enhancing Transparency in Reporting the Synthesis of Qualitative Research (ENTREQ) statements (Supplement 1).

PICOs were adapted to inform eligibility.

Population Study participants were adults with physician diagnosed or radiographic (Kellgren and Lawrence grade ≥2) hip or knee OA, or met classification criteria for OA at these joints [33]. If a study included people with other arthritis, e.g. rheumatoid arthritis, they were included if people with knee or hip OA were the largest proportion. Studies with participants awaiting total joint replacement were excluded.

Outcomes: perceptions of barriers and facilitators that influence uptake or maintenance of PA. Studies were included if they explored the factors/barriers/facilitators/motivation to engagement in PA, or addressed the experience of people with hip or knee OA regarding PA or exercise.

Study designs: Qualitative or mixed methods studies.

Language: Published in English.

Information sources

MEDLINE (Ovid MEDLINE(R) In-Process & Other Non-Indexed Citations and Ovid MEDLINE(R) 1946 to Present, OVID), EMBASE (1974 onwards, OVID interface), PhychINFO (1967 onwards, OVID), Web of Science, CINAHL, SPORTDiscus and Scopus were searched up to 31 of December 2015. Grey literature sources were explored, i.e. OpenGrey, NHS evidence. The search strategy was complemented by hand search of qualitative-research-centred journals screening of references of included articles and contacting researchers active in the field.

Search

For peer review only - http://bmjopen.bmj.com/site/about/guidelines.xhtml

The search strategy contained exhaustive keyword combinations for each of the four concepts of interest, i.e. knee or hip OA; PA/exercise; facilitators, barriers, motivation, uptake, maintenance; qualitative studies (Supplement 2).

Study selection

The search and study selection was conducted by two researchers independently (AMK & AsA). Endnote X7 was used for data management. Citations and abstracts were imported and duplicates removed. After title/abstract screening, full text of potentially relevant studies were assessed and additional information was sought from authors where necessary. If consensus was not reached between the two researchers, a third reviewer was consulted (AR).

Data collection and appraisal

All text under the sections of "results" and "findings" of the selected studies was considered as data items. Where findings and discussion were presented together, the whole section was considered for analysis. Data items were entered into and managed with NVivo 11 qualitative data analysis software (QSR International).

Quality appraisal aimed to assess the reporting, methodological rigor and conceptual consistency of the included studies [34] and to identify and discard low quality studies. Two approaches were used, which complement each other [32]: (a) the Critical Appraisal Skills Programme (CASP) Qualitative Checklist [35]. Studies were rated as high, medium and low quality if they met ≥8, 5-7, and 4 or fewer criteria respectively; (b) the evaluative criteria of credibility, transferability, dependability and confirmability that assess the trustworthiness of the study. Studies were rated high, medium, and low quality if they met ≥3, 2, or 1 and less criteria[36]. Two reviewers independently appraised the selected studies (AK, NE).

The phenomenon of interest was the description and interpretation of OA patients' perceptions and experiences regarding what facilitates, motivates or hinders them from engaging in PA. In addition, observed differences in facilitators and barriers to uptake and maintenance of PA (exercise and lifestyle PA) were also included.

Synthesis of results

Data were analysed by thematic synthesis[37]. First, authors' interpretations and informants' quotes were coded separately, line by line. Codes of original themes, subthemes and codes clearly referring to other types of arthritis where excluded from the synthesis. Next, descriptive themes were formed through code merging and grouping in a highly iterative process, creating a hierarchical tree. To form the analytical themes, a data driven analysis was initially conducted to allow an inductive interpretation. A group (AMK, NE, AR, JLD) review

For peer review only - http://bmjopen.bmj.com/site/about/guidelines.xhtml

meeting was held and the fit of this synthesis within theoretical models of behaviour change, motivation, human development and health was examined. The findings showed good fit with the biopsychosocial model of health[38] which was chosen to facilitate a more comprehensive and meaningful interpretation of the data and reporting of the findings. The descriptive themes were then re-examined and refined. At this point the research question was introduced to help infer the barriers and facilitators under the three domains of the biopsychosocial model. To enhance the credibility of the findings the synthesis was conducted by AMK and checked independently by NE.

Additional analysis. The descriptive study characteristics were examined in relation to the secondary research objectives. Due to insufficient evidence no further analysis was conducted.

RESULTS

Study selection

5,449 studies were identified, and after removing duplicates, 2,657 titles or/and abstracts were screened and 51 full-text papers were assessed. Seven authors were contacted for further information. Information was not provided for two studies, which were excluded. Ten studies were included [39-48](Figure 1).

Study characteristics

There were 173 participants, mainly middle aged to older, and female. Nine of ten studies reported qualitative methodologies (Table 1).

For peer review only

For peer review only - http://bmjopen.bmj.com/site/about/guidelines.xhtml

Study	Objectives	Country	Participants (number; diagnosis/ OA site; characteristics; sampling)	Methods (Data collection & analysis)	Findings	Relevance to Secondary Objectives (Exercise vs lifestyle PA; uptake vs maintenance)
**Campbell et al. (2001)[39]**	Compliance with a physiotherapy intervention.	UK	20 participants; Knee OA 14 female, age >45; Maximum variation sampling.	Interviews; constant comparative method	Factors related to compliance: moral obligation towards the physiotherapist (initial compliance); viewing exercise as beneficial, fitting exercises in daily life, perceived symptom severity, arthritis and comorbidity attitudes, exercise and OA experiences (continued compliance).	Exercise regime. Both initial and continued compliance explored.
**Fisken et al. (2015)[40]**	Reasons for ceasing participation in aqua based exercise.	New Zealand	11 participants; various OA sites, 10 hip or knee; female; age >60; purposeful sampling	Focus groups; general inductive thematic approach.	Main barriers: lack of appropriate classes and knowledgeable instructors, increase in pain, cold water and facilities.	Exercise regime. No uptake-maintenance distinction.
**Hammer et al. (2015)[41]**	Self-efficacy in relation to PA maintenance among maintainers and non-maintainers post-intervention	Denmark	15 participants; Hip OA; 8 female, age 65-74; Criterion based purposeful sampling.	Semi-structured interviews; directed content analysis.	Themes: mastery experiences, vicarious experiences, verbal persuasion, physiological and emotional states, altruism.	Exercise regimes. No uptake-maintenance distinction.
**Hendry et al. (2006)[42]**	Views towards exercise, determinants of acceptability and motivation, barriers.	UK	22 participants; Knee OA; 16 female, age 52-86; Purposeful sampling (inclusion/ exclusion criteria).	Interviews and focus group; Principles of Framework method of qualitative analysis.	Exercise participation determinants: perception of physical capacity, beliefs about exercise, motivational factors.	Exercise (broad definition). No uptake-maintenance distinction.
**Kabel et al. (2014)[43]**	Pain, social pressure and embarrassment in activity related decision making.	USA	10 participants; knee OA; 7 female, mean age 60; sampling method not clearly reported.	Interviews; Grounded theory or constant comparative method	Four PA-related patterns: Risk pain and embarrassment; risk pain, avoid embarrassment; avoid pain, risk embarrassment; avoid pain and embarrassment.	PA (living with OA). No uptake-maintenance distinction.
**Kaptein et al. (2014)[44]**	PA perception in the context of managing arthritis and multiple roles.	Canada	40 participants; 17 hip/knee OA, 16 RA, 4 both OA and RA, 3 other OA sites; 24 female, ages 29-72; purposeful sampling.	Focus groups; Qualitative content analysis	Positive PA perceptions, complex relationship between PA, arthritis and life roles (PA as potential cause of arthritis, reciprocal relationship, harms and benefits, perceived choices).	PA No uptake-maintenance distinction.
**Petursdottir et al. (2010)[45]**	Exercise experience. What determines whether people exercise	Iceland	12 participants; various OA sites, 10 hip or knee; 9 female, mean age 67 (50-81); Purposeful sampling	Interviews; Phenomenology (Vancouver School)	Barriers/ facilitators: internal (individual attributes and exercise experiences) and external (social and physical environment).	Exercise No uptake-maintenance distinction.
**Stone & Baker (2015)[46]**	Facilitators and barriers to regular PA	Canada	15 participants, Hip or/and knee OA; 9 female, age 30-85; Snowball sampling.	Semi-structured interview; Interpretational analysis	Facilitators: pain relief, clear communication from health-care professionals, social support. Barriers: pain, psychological distress, lack of support from health care professionals	PA No uptake-maintenance distinction.
**Thorstensson et al. (2006)[47]**	Underlying processes leading to response or non-response to exercise as treatment	Sweden	16 participants, knee OA; 6 female, age 39-64; purposeful sampling	Interviews; Phenomenography	Themes: to gain health, to become motivated, to experience the need for support, to experience resistance.	Exercise No uptake-maintenance distinction.
**Veenhof et al. (2006)[48]**	Factors that explain differences between patients who integrated activities in their daily lives or not.	Netherlands	12 participants; hip or knee OA; 8 female, ages 51-80; deliberate sampling for heterogeneity	Interviews; Grounded theory	Long-term goals and active involvement in the intervention related to greater adherence.	Exercise No uptake-maintenance distinction.

Table 1. Study characteristics

For peer review only - http://bmjopen.bmj.com/site/about/guidelines.xhtml

Appraisal of studies

All selected studies were of medium or high quality (Table 2). The research design and data analysis were not clear or well described in half of the studies and very few studies had clearly identified the relationship between the researcher and participants. Credibility, transferability and confirmability were met by almost all studies, although dependability only by two.

Table 2. Appraisal of studies

		**Campbell et al. (2001)**	**Fisken et al. (2015)**	**Hammer et al. (2015)**	**Hendry et al. (2006)**	**Kabel et al. (2014)**	**Kaptein et al. (2013)**	**Petursdottir et al. (2010)**	**Stone & Baker (2015)**	**Thorstensson et al. (2006)**	**Veenhof et al. (2006)**
CASP Qualitative Checklist		6/10	6/10	6/10	9/10	6/10	7/10	9/10	9/10	7/10	6/10
**1. Was there a clear statement of the aims of the research?**		✓	✓	✓	✓	✓	✓	✓	✓	✓	✓
**2. Is a qualitative methodology appropriate?**		✓	✓	✓	✓	✓	✓	✓	✓	✓	?
**3. Was the research design appropriate to address the aims of the research?**		?	✓	✓	✓	?	x	✓	✓	?	?
**4. Was the recruitment strategy appropriate to the aims of the research?**		✓	?	✓	✓	✓	✓	✓	✓	?	✓
**5. Was the data collected in a way that addressed the research issue?**		✓	?	x	✓	?	✓	✓	✓	?	✓
**6. Has the relationship between researcher and participants been adequately considered?**		?	?	x	✓	?	x	✓	?	✓	?
**7. Have ethical issues been taken into consideration?**		?	✓	✓	?	✓	✓	?	✓	✓	✓
**8. Was the data analysis sufficiently rigorous?**		?	?	?	✓	?	?	✓	✓	✓	✓
**9. Is there a clear statement of findings?**		✓	✓	?	✓	✓	✓	✓	✓	✓	✓
**10. How valuable is the research?**		✓	✓	✓	✓	✓	✓	✓	✓	✓	?
Trustworthiness	**Credibility**	✓		✓	✓	✓	✓	✓	✓	✓	✓
	**Transferability**	✓	✓	✓	✓	✓	✓	✓	✓	✓	
	**Dependability**								✓	✓	
	**Confirmability**	✓	✓	✓	✓		✓	✓	✓	✓	✓

✓ *= yes,* x= *no,* ?= *uncertain*

Synthesis of results

Barriers and facilitators are presented under the three conceptual domains, i.e. physical health, intrapersonal factors and social-environmental factors. Barriers and facilitators that appeared in at least three studies are

For peer review only

reported, to keep a balance between richness and applicability of the findings (Table 3; Supplement 3 for supporting references). When comparing exercise and PA focused studies, the themes were similar in context and equally represented in most cases. Where there are differences, these are reported.

Table 3. Barriers and facilitators: Themes, subthemes and number of supporting references

Domain	Major themes	Barriers	No of studies	No of ref/ces	Facilitators	No of studies	No of ref/ces
**Physical health**		Physical barriers and limitations (Pain and other symptoms; Perceived functional limitations)	9	94	PA for mobility, symptom relief and health (PA to maintain mobility; PA for symptom relief; PA for health)	9	34
**Intrapersonal /psychological factors**	Experience and beliefs about PA and OA	PA as non-effective, harmful or of doubtful effectiveness	6	36	Exercise as beneficial	7	60
		OA beliefs	5	17	Knowledge about exercise	3	8
	Behavioural regulation & attitude	Resigned to OA	5	10	Keep going despite OA	7	18
		Lack of motivation	6	14	Adjustments, prioritisation and personal effort (Adjusting PAs; Prioritising PA; Personal responsibility and effort in being physically active)	9	41
		Lacking behavioural regulation	4	23			
	Emotions	OA-related distress	6	23	Enjoyment	4	22
**Social Environment**	Health professionals	Lack of advice and encouragement from health professionals	5	22	Support from health professionals	8	50
	Social support	Social comparison as demotivating	5	15	Social support facilitating PA	7	43
		Lack of social support	4	8			

1. Physical health

Barriers. Physical barriers and limitations. Pain is aversive, stressful and inherent to living with OA[39-47]. It was mentioned as part of daily experience[45 46] or in relation to particular types of activities[40-42 44 46 47]. Along with fatigue and stiffness[44-46] these symptoms hindered the ability to engage in PA. There was a vicious cycle between symptoms and lack of exercise[41 42]. At an advanced stage of OA, PA was inhibited[42]. OA symptoms were aggravated by obesity and made PA more difficult[39 42 45]. Participants also discussed their sense of limited physical capacities and that one's body cannot manage PA requirements, resulting in loss of previous activity patterns[42-46]. For example, some talked about the need to choose between activities because of limited energy[44]. Old age and lack of physical fitness were also reported as perceived PA barriers[42 45].

*Facilitators***.** PA for mobility, symptom relief and health. Among those who held a physically active lifestyle maintaining or regaining their mobility was a strong motive for PA[39 41 45 46 48]. In most cases the aim was to keep functioning [39 42 44 47], in some it was so specific as to prevent joint surgery[41 48]. Pain relief is another strong

For peer review only - http://bmjopen.bmj.com/site/about/guidelines.xhtml

motive for being physically active and active individuals were more likely those who had experienced pain reduction [39 41 45 46 48]. A few informants presented a "no pain, no maintenance" pattern, where pain cessation was followed by dropping exercise[39 48]. Improvements in other symptoms, such as stiffness and joint stability, were sufficient reasons for being active, even when pain remained[39 45]. Maintaining good general health and physical condition were also reasons for being physically active[41 42 44 45 47]. This facilitator was closely linked to a positive, beneficial PA experience and subsequent positive attitude towards PA, which is a crucial facilitator discussed below.

2. Intrapersonal/ psychological factors

Experience and beliefs about exercise. *Facilitators.* Exercise as beneficial. Experiencing benefits from exercise participation, which in most of the studies was related to engagement in an exercise intervention, helped shaping positive beliefs and motivated individuals towards continuing exercise[39-42 45 47 48]. A sense of psychosomatic well-being was an important component of this theme[40-42 45 47]. Improvement in coping with OA [47] and sleep [45] were mentioned.

Knowledge about exercise in OA. Accurate knowledge of the importance of exercise in OA, acquired through health care, physiotherapy and exercise interventions, was an important facilitator[41 45-47]. It led to awareness regarding exercise benefits and helped in making correct interpretations of exercise experiences.

Both the above themes emerged from exercise-focused studies only.

*Barriers***.** PA as non-effective, harmful or of doubtful effectiveness. The belief that PA does not help or might further deteriorate their condition, hindered people from being active[39 41 42 44 46 47]. Experiencing activity-related pain in the joint, for example, was often interpreted as PA exacerbating OA, which stemmed from the understanding of OA as a "wear and tear" condition[42 44 47]. Not experiencing the anticipated beneficial effects during exercise interventions was a reason for distrust in PA as an effective means of treatment [39 41 42 47]. Also, early negative experiences with sports resulted in exercise avoidance[45].

OA beliefs. Beliefs that nothing can be done regarding the condition[42 45 47] and that overuse was the cause of OA[39 42 44] were linked to less inclination towards being physically active. In one study the relationship between PA and OA was discussed as bi-directional[44]. These beliefs were mostly reported in exercise-focused studies (four exercise studies with one PA-focused study also revealing such beliefs).

Daily activities as PA. This theme revolved around beliefs about non-leisure PA[42 44 45 47]. However, there were no consistent patterns across studies to be clearly classified as barriers or facilitators. For example, non-leisure activities were viewed as a sufficient amount of PA by some[42 45 47] and as insufficient by others[42].

For peer review only - http://bmjopen.bmj.com/site/about/guidelines.xhtml

Behavioural regulation and attitude. *Facilitators.* Keep going despite OA. Authors' interpretations related to this concept varied, e.g. determination to take control of arthritis[42], perseverance[47], personality traits of adaptability and initiative[45], belief that there are "things patients can do" about their OA[39], motivation towards long-term goals[48]. The importance of keeping a positive attitude was also discussed[44 45]. In two studies the relevant participant quotes were presented under the themes "risking embarrassment"[43] and "bi-directional impact between PA and arthritis"[44].

Adjustments, prioritisation and personal effort. Physically active individuals described how they were making short or long term modifications to their PA[40-45], such as finding a type of exercise that was suitable for their physical abilities[40-42 45], adjusting PA intensity to their current condition[41 43 45], even changing their job[44]. This task of continuously adjusting PAs was quite demanding[45]. Prioritising PA and fitting it into a routine was mentioned by a number of physically active participants and reflected the importance they assigned to PA[39 42 47 48]. Active participants also acknowledged they were the main agents in managing their condition and they were consciously making efforts to stay active[39 42 44 47].

Barriers. Lack of motivation Participants in different studies referred to a lack of motivation or goal, laziness and boredom towards exercise[39 41 42 45 47 48]. These type of barriers were reported in the exercise-focused studies only and were not further explored.

Lacking behavioural regulation. In the face of the demands of other life roles and a busy schedule, especially family related, inactive participants were not prioritising PAs[39 42 44 47]. In two studies informants referred to not finding a PA suitable for their current condition[40 42]. In one study low self-regulation was the reason given for not exercising regularly[42].

Resigned to OA. In half of the studies informants expressed a resigned attitude towards making an effort to be active[39 42 45-47]. Reflecting fatalistic beliefs about OA and feelings of helplessness, this attitude was linked to attenuated motivation for being physically active.

Emotions. *Facilitators.* Enjoyment. Enjoying exercise in general or a particular type of exercise facilitated its continuation[40 42 45]. This facilitator of engagement emerged in the exercise-focused studies only.

Barriers. OA related distress. Living with OA means adjusting to a reality of decreased physical functioning and in several cases participants talked about this experience of giving up activities, being unable to meet life roles and daily demands as distressing or embarrassing[39 41 43-46]. Mental stress[41], extreme unhappiness and paralyzing fatigue[45], feeling broken and mentally depressed[46], weakness[44] were used.

3a. Social Environment

For peer review only - http://bmjopen.bmj.com/site/about/guidelines.xhtml

Health professionals. *Facilitators.* Support from health professionals. Physiotherapists exerted great influence on the patients' PA/ exercise habits[39 41 42 45 46 48]. Providing instructions, education, encouragement and rapport with the patient were means of facilitating exercise. Advice and prescription by doctors was another facilitator[42 45]. Supervision during exercise was valued[39-42 47 48]. Good supervision gave participants the reassurance that what they were doing was appropriate and good for their body[42], which they needed[46], and motivated them to exercise[39 41 47 48]. Support from health professionals was reported in four exercise studies and one PA-focused.

Barriers. Lack of support from health professionals. Ambiguous, no or conflicting information from doctors regarding PA was a barrier[39 42 45 46]. In one study, the instructor not having specialized OA training was the reason that lead participants to discontinue their exercise[40].

Social support. *Facilitators.* Social support facilitating PA. Social support as a facilitator was mainly discussed in the context of exercising in a group, as well as support from family and friends. Feeling comfortable and motivated, even inspired when exercising with people of similar physical abilities and age emerged as an advantage of PA programs[39-42 44 46]. This was of particular importance when someone was first introduced to PA[41]. Opportunities to socialize were also an advantage of group PA[40 42]. In addition, psychological and instrumental support from family and friends emerged as an asset of physically active participants, taking the form of active encouragement, expression of interest and understanding, an exercise buddy or role model[41 44-46]. Community based support was mentioned as PA promoting[46]. This theme stemmed equally from exercise and PA centered studies, although the focus of the former was on group exercise and the latter on important others' support for an active lifestyle.

Barriers. Social comparison as demotivating. Although this concept did not explicitly appear as an authors' interpretation it emerged from informants' quotes. Being unable to keep up with others when participating in PA was a PA barrier as it provoked feelings of embarrassment and distress[39-43]. This barrier was reported in four exercise studies and one PA-focused.

Lack of social support The lack of social support from peers and family as a barrier was discussed in relation to lack of understanding and encouragement from the person's family and social[39 44 45] and work environment[44].

3b. Physical Environment

Barriers. The cost of exercise classes[40 44 45], limited accessibility[45 47] and lack of availability of appropriate modes[45 46], as well as cold weather and issues regarding safety[40] were the reported environmental barriers to PA.

DISCUSSION

For peer review only - http://bmjopen.bmj.com/site/about/guidelines.xhtml

For peer review only

This SR is the first to synthesize the existing qualitative research on barriers and facilitators to PA in knee and hip OA. Pain and physical limitations, absence of positive PA experiences and beliefs, resigned attitude and distress due to OA, lack of behavioral regulation, lack of support from health professionals and negative social comparisons when exercising in a group were important PA barriers. Symptom relief and mobility, positive exercise experiences and beliefs, knowledge, enjoying exercise, a “keep going” attitude, adjusting and prioritising PA, having professional and social support were important PA facilitators. Overall the findings are consistent with known PA correlates in exercise psychology[49], theories of behavioral change[50] and results emanating from existing SRs in general (i.e. non-OA specific) populations that share common characteristics with OA patients[51-53]. Present findings also outline a unique profile of PA barriers and facilitators in lower limb OA.

Factors related to physical health, specifically pain and physical function, were the most consistently reported. This indicates that OA has a central role and impact in people’s lives and experiences, which is in line with previous qualitative findings that pain discussions by people with OA differ in frequency and quality in comparison to healthy individuals[54]. Importantly, physical barriers are reported both by active and inactive people. Therefore, physical barriers alone cannot explain PA behavior- with the exception of patients at very advanced stages of OA[55]. Intrapersonal and social variables are crucial in PA behaviors reported earlier[53].

The identified barriers and facilitators are not stand-alone and independent entities, but manifest a complex interplay. Personal experience, knowledge and beliefs about PA, exercise and OA were interwoven concepts and formed the basis of PA behavior. Experiencing benefits from participation in an exercise program- which was the case in most of the included studies- shapes a positive attitude towards PA[51 52 56-58]. Accurate knowledge regarding PA, exercise and OA bolstered a positive interpretation of and predisposition towards PA experience. Viewing pain as manageable versus inevitable elicited different behaviors[59 60] and, not surprisingly, patient education is a core component of health care and OA management[61 62]. Support from health professionals becomes crucial as they can provide rationale and motivation for PA[56] and shape the patients’ health experience[54]. The above factors and available social support are not independent from, but influence motivation, attitude and behavioral regulation.

Most of the PA barriers and facilitators emerged under the psychological/intrapersonal domain and were mostly OA-related. The data analysis allowed for new insights into the original studies, such as the emerging theme of OA-related distress and two distinct patterns in attitude, beliefs, motivation and behavioral regulation- one facilitating and the other hindering PA. Pain and its multifaceted impact is a source of distress in

For peer review only - http://bmjopen.bmj.com/site/about/guidelines.xhtml

OA[24]. In turn, anxiety and depressive symptoms, which are more prevalent in people with arthritis[63], are predictors of poorer function[64 65] and pain[29 66-68]. Still cognitive processes underlying the distinct patterns are missing, e.g. what distinguishes those who, for a given level of structural disease-severity and OA-related pain, exhibit a positive attitude and behavioral regulation from those who are resigned, cope ineffectively with OA-stress and lack self-regulation? Explanations involving distinguishing processes and participant characteristics might lie in theoretical frameworks of behavior change and health, which are absent in the included studies, with one exception[41]. For example, self-efficacy, self-determination and need satisfaction are precursors of behavior in theories which have been applied to predicting and promoting PA[69 70], whereas sense of control is a common concept in the stress and coping literature[71]. Future research should make use of theoretical knowledge and approaches to enable targeted and more effective research and interventions[72].

All the findings reported were grounded in the three studies that scored "high" at both sets of quality criteria[42 45 46], along with the seven medium quality studies, which confirms their trustworthiness. However, aspects of methodology were poorly reported or explored in the selected studies, particularly those of medium quality. A consideration of the researcher-participant relationship and employing an external auditor for the decision trail (dependability) should be used to increase confidence in the findings.

The SR findings hold implications for clinical practice. All healthcare professionals who manage people with lower-limb OA have a key role in facilitating PA through their advice, attitude towards OA and decision to seek multidisciplinary input e.g. from physiotherapy. Even without directed advice to increase PA, health and condition-related advice and a supportive stance from healthcare professionals can influence decisions related to PA engagement[73]. In the absence of education, people with OA tend to draw from lay and often fatalistic beliefs of PA and exercise in OA. An individual assessment of the experienced impact of pain and disability, personal attitudes and circumstances, educating about the role of PA in OA management, offering feasible yet specific PA prescription and encouragement can have an impact on the persons' PA and exercise behavior. Pain and stress-related coping strategies, guidance through exercise prescription and effective communication are the main components of established arthritis self-management programs[74]. Increasing the time designated to each patient within the health care system could allow for such practices to take place. Counselling referral and online educational tools could also affect PA behaviour.

Based on the available qualitative evidence it was not possible to adequately explore the secondary SR questions, an issue which has been previously reported [53 75]. Only three studies focused on lifestyle PA, which is surprising considering the paradigm shift in the health literature from exercise promotion to a combination of

For peer review only - http://bmjopen.bmj.com/site/about/guidelines.xhtml

PA promotion and sedentary time reduction[76]. Also, only one study made the distinction between PA uptake and maintenance, despite the recognition that these two stages entail different determinants[69 77 78]. In the case of people living with OA, the factors and processes leading to uptake and maintenance of overall PA need to be further explored and understood.

This SR has applied rigorous methods and provides an in-depth and meaningful understanding of the phenomenon of interest based on the accumulated existing qualitative evidence, thus moving one step forward from existing SRs[21 22]. Gaps in the existing literature were also identified. With regards to data synthesis, coding participants' quotes and authors' interpretations separately allowed aspects of the phenomenon not captured by the original studies to come to light. During data synthesis, peer review by a multidisciplinary team took place to enhance credibility. The main reviewer's background is clinical psychology, which might be reflected in the emphasis on the "psychological" component of PA barriers and facilitators.

There are certain limitations to this study. The majority of the included studies were exercise-focused, therefore might not accurately or fully represent barriers and facilitators to lifestyle PA (of which engaging in structured exercise programs is type or form). Due to resource limitations, studies not written in English were excluded. Two relevant studies were also excluded because they were in a conference abstract form and additional data were not available[79 80]. Lastly, due to the nature of the evidence, directions of the relationships and interactions among the identified factors cannot be drawn.

In summary, there is a complex interplay among the physical, intrapersonal, psychological and socio-environmental barriers and facilitators of exercise and PA that bears similarities with other chronic diseases, but also includes characteristics specific to OA. Personal experiences, beliefs, attitudes and emotions, as well as the social environment, i.e. health care and social support, are dynamic factors shaping PA behavior. Considering that OA becomes more prevalent with age, it is important and challenging to make sustained lifestyle changes that will have a positive impact on an individual, as well as at a health-care system level. With the aim of identifying effective practices to help people with OA become more active, future research should involve behavioral intervention studies to address the factors identified above.

Amendments to the protocol Please see supplement 4.

Competing interests No competing interests to declare.

Funding This review comprises part of the research requirements of a PhD to be completed by AMK, funded by the MRC-Arthritis Research UK Centre for Musculoskeletal Ageing Research.

For peer review only

Authors' contributions Study concept and design: JLD, AR, AMK, RK, AbA, NE. Searches: AMK, AsA. Study appraisal: AMK, NE. Data analysis: AMK, checked by NE. Data interpretation: AMK, checked by JLD, AR, NE. Manuscript draft: AMK. Manuscript review and input: JLD, AR, AbA, NE, RK. All authors provided feedback and approved the final draft.

Data sharing: Qualitative synthesis level electronic data (NVivo 11) are available upon request from the corresponding author.

References

1. NICE. Osteoarthritis care and management in adults. *NICE clinical guideline 177* 2014
2. McAlindon TE, Bannuru RR, Sullivan MC, et al. OARSI guidelines for the non-surgical management of knee osteoarthritis. *Osteoarthritis and cartilage / OARS, Osteoarthritis Research Society* 2014;22(3):363-88. doi: 10.1016/j.joca.2014.01.003
3. Caspersen CJ, Powell KE, Christenson GM. Physical activity, exercise, and physical fitness: definitions and distinctions for health-related research. *Public Health Reports* 1985;100(2):126-31.
4. Fransen M, McConnell S, Harmer AR, et al. Exercise for osteoarthritis of the knee: a Cochrane systematic review. *British Journal of Sports Medicine* 2015;49(24):1554-7.
5. Fransen M, McConnell S, Hernandez-Molina G, et al. Exercise for osteoarthritis of the hip. *Cochrane Database of Systematic Reviews 2014* 2014(4) doi: 10.1002/14651858.CD007912.pub2
6. Anwer S, Alghadir A, Brismee JM. Effect of Home Exercise Program in Patients With Knee Osteoarthritis: A Systematic Review and Meta-analysis. *Journal of Geriatric Physical Therapy* 2016;39(1):38-48.
7. Juhl C, Christensen R, Roos EM, et al. Impact of exercise type and dose on pain and disability in knee osteoarthritis: a systematic review and meta-regression analysis of randomized controlled trials. *Arthritis & Rheumatology* 2014;66(3):622-36.
8. Boesen MI, Boesen A, Koenig MJ, et al. Ultrasonographic investigation of the Achilles tendon in elite badminton players using color Doppler. *American Journal of Sports Medicine* 2006;34(12):2013-21.
9. Uthman OA, van der Windt DA, Jordan JL, et al. Exercise for lower limb osteoarthritis: systematic review incorporating trial sequential analysis and network meta-analysis.[Reprint of BMJ. 2013;347:f5555; PMID: 24055922]. *British Journal of Sports Medicine* 2014;48(21):1579.
10. Pisters MF, Veenhof C, van Meeteren NL, et al. Long-term effectiveness of exercise therapy in patients with osteoarthritis of the hip or knee: a systematic review. *Arthritis & Rheumatism* 2007;57(7):1245-53.
11. Pisters MF, Veenhof C, de Bakker DH, et al. Behavioural graded activity results in better exercise adherence and more physical activity than usual care in people with osteoarthritis: a cluster-randomised trial. *Journal of Physiotherapy* 2010;56(1):41-7.
12. Batsis JA, Germain CM, Vasquez E, et al. Physical Activity Predicts Higher Physical Function in Older Adults: The Osteoarthritis Initiative. *J Phys Act Health* 2016;13(1):6-16. doi: 10.1123/jpah.2014-0531
13. Chmelo E, Nicklas B, Davis C, et al. Physical activity and physical function in older adults with knee osteoarthritis. *J Phys Act Health* 2013;10(6):777-83.

For peer review only - http://bmjopen.bmj.com/site/about/guidelines.xhtml

For peer review only

14. Lin W, Alizai H, Joseph GB, et al. Physical activity in relation to knee cartilage T2 progression measured with 3 T MRI over a period of 4 years: data from the Osteoarthritis Initiative. *Osteoarthritis and cartilage / OARS, Osteoarthritis Research Society* 2013;21(10):1558-66. doi: 10.1016/j.joca.2013.06.022
15. Loprinzi PD, Sheffield J, Tyo BM, et al. Accelerometer-determined physical activity, mobility disability, and health. *Disability and health journal* 2014;7(4):419-25. doi: 10.1016/j.dhjo.2014.05.005 [published Online First: 2014/09/17]
16. Austin S, Qu H, Shewchuk RM. Association between adherence to physical activity guidelines and health-related quality of life among individuals with physician-diagnosed arthritis. *Quality of life research : an international journal of quality of life aspects of treatment, care and rehabilitation* 2012;21(8):1347-57. doi: 10.1007/s11136-011-0046-x [published Online First: 2011/11/01]
17. Austin J, Williams WR, Hutchison S. Multidisciplinary management of elderly patients with chronic heart failure: five year outcome measures in death and survivor groups. *European Journal of Cardiovascular Nursing* 2009;8(1):34-39 6p. doi: 10.1016/j.ejcnurse.2008.04.005
18. Herbolsheimer F, Schaap LA, Edwards MH, et al. Physical Activity Patterns Among Older Adults With and Without Knee Osteoarthritis in Six European Countries. *Arthritis Care & Research* 2016;68(2):228-36. doi: 10.1002/acr.22669
19. Marks R. Knee osteoarthritis and exercise adherence: a review. *Current Aging Science* 2012;5(1):72-83.
20. Marks R, Allegrante JP. Chronic osteoarthritis and adherence to exercise: a review of the literature. *Journal of Aging & Physical Activity* 2005;13(4):434-60.
21. Stubbs B, Hurley M, Smith T. What are the factors that influence physical activity participation in adults with knee and hip osteoarthritis? A systematic review of physical activity correlates. *Clinical Rehabilitation* 2015;29(1):80-94.
22. Veenhof C, Huisman PA, Barten JA, et al. Factors associated with physical activity in patients with osteoarthritis of the hip or knee: a systematic review. *Osteoarthritis & Cartilage* 2012;20(1):6-12.
23. Dobson F, Bennell KL, French SD, et al. Barriers and Facilitators to Exercise Participation in People with Hip and/or Knee Osteoarthritis: Synthesis of the Literature Using Behavior Change Theory. *American journal of physical medicine & rehabilitation / Association of Academic Physiatrists* 2016 doi: 10.1097/phm.0000000000000448 [published Online First: 2016/03/06]
24. Hawker GA, Stewart L, French MR, et al. Understanding the pain experience in hip and knee osteoarthritis--an OARSI/OMERACT initiative. *Osteoarthritis and cartilage / OARS, Osteoarthritis Research Society* 2008;16(4):415-22. doi: 10.1016/j.joca.2007.12.017
25. Sanchez-Heran A, Agudo-Carmona D, Ferrer-Pena R, et al. Postural Stability in Osteoarthritis of the Knee and Hip: Analysis of Association With Pain Catastrophizing and Fear-Avoidance Beliefs. *PM R* 2016;8(7):618-28. doi: 10.1016/j.pmrj.2015.11.002
26. Holla JF, van der Leeden M, Knol DL, et al. Predictors and outcome of pain-related avoidance of activities in persons with early symptomatic knee osteoarthritis: a five-year followup study. *Arthritis Care Res (Hoboken)* 2015;67(1):48-57. doi: 10.1002/acr.22381
27. Holla JF, Sanchez-Ramirez DC, van der Leeden M, et al. The avoidance model in knee and hip osteoarthritis: a systematic review of the evidence. *J Behav Med* 2014;37(6):1226-41. doi: 10.1007/s10865-014-9571-8

For peer review only - http://bmjopen.bmj.com/site/about/guidelines.xhtml

28. Herbolsheimer F, Schaap LA, Edwards MH, et al. Physical Activity Patterns Among Older Adults With and Without Knee Osteoarthritis in Six European Countries. *Arthritis Care Res (Hoboken)* 2016;68(2):228-36. doi: 10.1002/acr.22669
29. Somers TJ, Keefe FJ, Godiwala N, et al. Psychosocial factors and the pain experience of osteoarthritis patients: new findings and new directions. *Curr Opin Rheumatol* 2009;21(5):501-6. doi: 10.1097/BOR.0b013e32832ed704
30. Wideman TH, Finan PH, Edwards RR, et al. Increased sensitivity to physical activity among individuals with knee osteoarthritis: relation to pain outcomes, psychological factors, and responses to quantitative sensory testing. *Pain* 2014;155(4):703-11. doi: 10.1016/j.pain.2013.12.028
31. Arksey HOM, L. Scoping studies: towards a methodologica framework. *International Journal of Social Research Methodology* 2005:19-32.
32. Kanavaki AM, Rushton A, Klocke R, et al. Barriers and facilitators to physical activity in people with hip or knee osteoarthritis: protocol for a systematic review of qualitative evidence. *BMJ Open* 2016;6(11):e012049. doi: 10.1136/bmjopen-2016-012049
33. Altman RD. Criteria for classification of clinical osteoarthritis. *The Journal of rheumatology Supplement* 1991;27:10-2. [published Online First: 1991/02/01]
34. K H. Critical appraisal of qualitative research. In: Noyes J BA, Hannes K, Harden A, Harris J, Lewin S, Lockwood C ed. Supplementary Guidance for Inclusion of Qualitative Research in Cochrane Systematic Reviews of Interventions Version 1 (updated August 2011): Cochrane Collaboration Qualitative Methods Group 2011.
35. Critical Appraisal Skills Programme (CASP). CASP Checklists (http://wwwcasp-uknet/ - !checklists/cb36) Oxford: CASP, 2014.
36. Creswell JW. Qualitative Inquiry and Research Design. Thousand Oaks, California: SAGE Publications Inc. 2007.
37. Barnett-Page E, Thomas J. Methods for the synthesis of qualitative research: a critical review. *BMC Med Res Methodol* 2009;9:59. doi: 10.1186/1471-2288-9-59
38. Engel GL. The clinical application of the biopsychosocial model. *Am J Psychiatry* 1980;137(5):535-44. doi: 10.1176/ajp.137.5.535
39. Campbell R, Evans M, Tucker M, et al. Why don't patients do their exercises? Understanding non-compliance with physiotherapy in patients with osteoarthritis of the knee. *Journal of Epidemiology and Community Health* 2001;55(2):132-38. doi: 10.1136/jech.55.2.132
40. Fisken AL, Waters DL, Hing WA, et al. Perceptions towards aqua-based exercise among older adults with osteoarthritis who have discontinued participation in this exercise mode. *Australasian Journal on Ageing* 2015 doi: 10.1111/ajag.12167
41. Hammer NM, Bieler T, Beyer N, et al. The impact of self-efficacy on physical activity maintenance in patients with hip osteoarthritis – a mixed methods study. *Disability and Rehabilitation* 2015:1-14. doi: 10.3109/09638288.2015.1107642
42. Hendry M, Williams NH, Markland D, et al. Why should we exercise when our knees hurt? A qualitative study of primary care patients with osteoarthritis of the knee. *Family Practice* 2006;23(5):558-67. doi: 10.1093/fampra/cml022
43. Kabel A, Dannecker EA, Shaffer VA, et al. Osteoarthritis and social embarrassment: Risk, Pain, And avoidance. *SAGE Open* 2014;4(2) doi: 10.1177/2158244014537649
44. Kaptein SA, Backman CL, Badley EM, et al. Choosing where to put your energy: a qualitative analysis of the role of physical activity in the lives of working adults with arthritis. *Arthritis care & research* 2013;65(7):1070-6.
45. Petursdottir U, Arnadottir SA, Halldorsdottir S. Facilitators and Barriers to Exercising Among People With Osteoarthritis: A Phenomenological Study. *Physical Therapy* 2010;90(7):1014-25. doi: 10.2522/ptj.20090217

For peer review only - http://bmjopen.bmj.com/site/about/guidelines.xhtml

46. Stone RC, Baker J. Painful Choices: A Qualitative Exploration of Facilitators and Barriers to Active Lifestyles Among Adults With Osteoarthritis. *J Appl Gerontol* 2015 doi: 10.1177/0733464815602114
47. Thorstensson CA, Roos EM, Petersson IF, et al. How do middle-aged patients conceive exercise as a form of treatment for knee osteoarthritis? *Disability and Rehabilitation* 2006;28(1):51-59. doi: 10.1080/09638280500163927
48. Veenhof C, van Hasselt TJ, Koke AJA, et al. Active involvement and long-term goals influence long-term adherence to behavioural graded activity in patients with osteoarthritis: a qualitative study. *Australian Journal of Physiotherapy* 2006;52(4):273-78.
49. Biddle SJH, Mutrie, N. Psychology of physical activity. Determinants, well-being & interventions. 2nd ed. New York: Routledge 2008:33-160.
50. Kwasnicka D, Dombrowski SU, White M, et al. Theoretical explanations for maintenance of behaviour change: a systematic review of behaviour theories. *Health Psychol Rev* 2016;10(3):277-96. doi: 10.1080/17437199.2016.1151372
51. Franco MR, Tong A, Howard K, et al. Older people's perspectives on participation in physical activity: a systematic review and thematic synthesis of qualitative literature. *Br J Sports Med* 2015;49(19):1268-76. doi: 10.1136/bjsports-2014-094015
52. Morgan F, Battersby A, Weightman AL, et al. Adherence to exercise referral schemes by participants - what do providers and commissioners need to know? A systematic review of barriers and facilitators. *BMC Public Health* 2016;16:227. doi: 10.1186/s12889-016-2882-7
53. Rhodes RE, Quinlan A. Predictors of physical activity change among adults using observational designs. *Sports Med* 2015;45(3):423-41. doi: 10.1007/s40279-014-0275-6
54. Gignac MA, Davis AM, Hawker G, et al. "What do you expect? You're just getting older": A comparison of perceived osteoarthritis-related and aging-related health experiences in middle- and older-age adults. *Arthritis Rheum* 2006;55(6):905-12. doi: 10.1002/art.22338 [published Online First: 2006/12/02]
55. Liu SH, Driban JB, Eaton CB, et al. Objectively Measured Physical Activity and Symptoms Change in Knee Osteoarthritis. *Am J Med* 2016;129(5):497-505 e1. doi: 10.1016/j.amjmed.2015.12.029
56. Horne M, Tierney S. What are the barriers and facilitators to exercise and physical activity uptake and adherence among South Asian older adults: a systematic review of qualitative studies. *Prev Med* 2012;55(4):276-84. doi: 10.1016/j.ypmed.2012.07.016
57. Wilcox S, Ananian CD, Abbott J, et al. Perceived exercise barriers, enablers, and benefits among exercising and nonexercising adults with arthritis: Results from a qualitative study. *Arthritis & Rheumatism-Arthritis Care & Research* 2006;55(4):616-27. doi: 10.1002/art.22098
58. Peeters GM, Brown WJ, Burton NW. Psychosocial factors associated with increased physical activity in insufficiently active adults with arthritis. *J Sci Med Sport* 2015;18(5):558-64. doi: 10.1016/j.jsams.2014.08.003
59. Main CJ, Williams AC. Musculoskeletal pain. *BMJ* 2002;325(7363):534-7.
60. Ross MM, Carswell A, Hing M, et al. Seniors' decision making about pain management. *J Adv Nurs* 2001;35(3):442-51.
61. NICE. Osteoarthritis care and management in adults. NICE clinical guideline, 2014.
62. Larmer PJ, Reay ND, Aubert ER, et al. Systematic review of guidelines for the physical management of osteoarthritis. *Arch Phys Med Rehabil* 2014;95(2):375-89. doi: 10.1016/j.apmr.2013.10.011

For peer review only - http://bmjopen.bmj.com/site/about/guidelines.xhtml

63. He Y, Zhang M, Lin EH, et al. Mental disorders among persons with arthritis: results from the World Mental Health Surveys. *Psychol Med* 2008;38(11):1639-50. doi: 10.1017/S0033291707002474
64. Dekker J, Boot B, van der Woude LH, et al. Pain and disability in osteoarthritis: a review of biobehavioral mechanisms. *J Behav Med* 1992;15(2):189-214.
65. Sharma L, Cahue S, Song J, et al. Physical functioning over three years in knee osteoarthritis: role of psychosocial, local mechanical, and neuromuscular factors. *Arthritis Rheum* 2003;48(12):3359-70. doi: 10.1002/art.11420
66. Edwards RR, Dworkin RH, Sullivan MD, et al. The Role of Psychosocial Processes in the Development and Maintenance of Chronic Pain. *The journal of pain : official journal of the American Pain Society* 2016;17(9 Suppl):T70-92. doi: 10.1016/j.jpain.2016.01.001
67. de Rooij M, van der Leeden M, Avezaat E, et al. Development of comorbidity-adapted exercise protocols for patients with knee osteoarthritis. *Clin Interv Aging* 2014;9:829-42. doi: 10.2147/CIA.S55705
68. Volders S, Boddez Y, De Peuter S, et al. Avoidance behavior in chronic pain research: a cold case revisited. *Behav Res Ther* 2015;64:31-7. doi: 10.1016/j.brat.2014.11.003
69. Deci EL, Ryan RM. Self-determination theory: a macrotheory of human motivation, development, and health. *Canadian Psychology* 2008;49(3):182-85.
70. Bandura A. Self-efficacy: toward a unifying theory of behavioral change. *Psychol Rev* 1977;84(2):191-215.
71. Jensen MP, Turner JA, Romano JM, et al. Coping with chronic pain: a critical review of the literature. *Pain* 1991;47(3):249-83.
72. Michie S. Designing and implementing behaviour change interventions to improve population health. *Journal of health services research & policy* 2008;13 Suppl 3:64-9. doi: 10.1258/jhsrp.2008.008014
73. Horne M, Skelton D, Speed S, et al. The influence of primary health care professionals in encouraging exercise and physical activity uptake among White and South Asian older adults: experiences of young older adults. *Patient Educ Couns* 2010;78(1):97-103. doi: 10.1016/j.pec.2009.04.004
74. Lorig KR, Mazonson PD, Holman HR. Evidence suggesting that health education for self-management in patients with chronic arthritis has sustained health benefits while reducing health care costs. *Arthritis Rheum* 1993;36(4):439-46.
75. Koeneman M, Verheijden M, Chinapaw M, et al. Determinants of physical activity and exercise in healthy older adults: a systematic review. *International Journal of Behavioral Nutrition & Physical Activity* 2011;8
76. Katzmarzyk PT. Physical Activity, Sedentary Behavior, and Health: Paradigm Paralysis or Paradigm Shift? *Diabetes* 2010;59(11):2717-25. doi: 10.2337/db10-0822
77. Prochaska JO, DiClemente CC. Stages and processes of self-change of smoking: toward an integrative model of change. *J Consult Clin Psychol* 1983;51(3):390-5.
78. Kinnafick FE, Thogersen-Ntoumani C, Duda JL. Physical activity adoption to adherence, lapse, and dropout: a self-determination theory perspective. *Qual Health Res* 2014;24(5):706-18. doi: 10.1177/1049732314528811
79. Gay C EB, Levyckyj C, Chabaud A, Coudeyre E. Facilitators and Barriers in physical activity engagement for knee osteoarthritis patients. 30th Annual Congress of the French Society of Physical and Rehabilitation Medicine: Annals of Physical and Rehabilitation Medicine, 2015.
80. Jinks C, Moore A, Holden MA, et al. UPTAKE AND MAINTENANCE OF PHYSIOTHERAPY-LED EXERCISE AND GENERAL PHYSICAL ACTIVITY IN

For peer review only

For peer review only - http://bmjopen.bmj.com/site/about/guidelines.xhtml

OLDER ADULTS WITH KNEE PAIN: THE BEEP LONGITUDINAL QUALITATIVE STUDY. *Rheumatology* 2015;54:36-36.

Figure 1. Study selection PRISMA flow diagram

For peer review only

Figure 1. Study selection PRISMA flow diagram

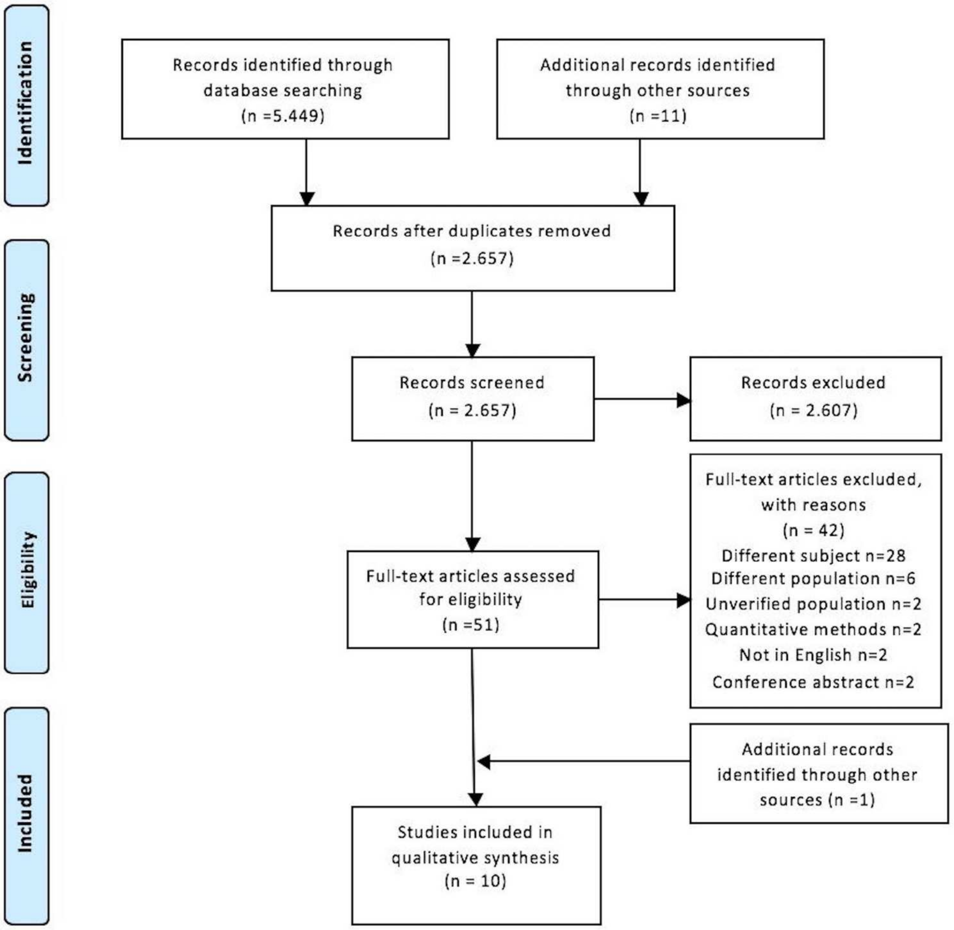

From: Moher D, Liberati A, Tetzlaff J, Altman DG, The PRISMA Group (2009). *Preferred Reporting Items for Systematic Reviews and Meta-Analyses*: The PRISMA Statement. PLoS Med 6(7): e1000097. doi:10.1371/journal.pmed1000097

For more information, visit www.prisma-statement.org.

148x183mm (300 x 300 DPI)

Supplement 1. PRISMA checklist of items to include when reporting a systematic review or meta-analysis

Section/topic	Checklist item	Reported on page #
**TITLE**		
Title	Identify the report as a systematic review, meta-analysis, or both.	1
**ABSTRACT**		
Structured summary	Provide a structured summary including, as applicable: background; objectives; data sources; study eligibility criteria, participants, and interventions; study appraisal and synthesis methods; results; limitations; conclusions and implications of key findings; systematic review registration number.	1
**INTRODUCTION**		
Rationale	Describe the rationale for the review in the context of what is already known.	2
Objectives	Provide an explicit statement of questions being addressed with reference to participants, interventions, comparisons, outcomes, and study design (PICOS).	3
**METHODS**		
Protocol and registration	Indicate if a review protocol exists, if and where it can be accessed (e.g., Web address), and, if available, provide registration information including registration number.	3
Eligibility criteria	Specify study characteristics (e.g., PICOS, length of follow-up) and report characteristics (e.g., years considered, language, publication status) used as criteria for eligibility, giving rationale.	3
Information sources	Describe all information sources (e.g., databases with dates of coverage, contact with study authors to identify additional studies) in the search and date last searched.	3
Search	Present full electronic search strategy for at least one database, including any limits used, such that it could be repeated.	3

For peer review only

Section/topic	Checklist item	Reported on page #
Study selection	State the process for selecting studies (i.e., screening, eligibility, included in systematic review, and, if applicable, included in the meta-analysis).	4
Data collection process	Describe method of data extraction from reports (e.g., piloted forms, independently, in duplicate) and any processes for obtaining and confirming data from investigators.	4
Data items	List and define all variables for which data were sought (e.g., PICOS, funding sources) and any assumptions and simplifications made.	4
Risk of bias in individual studies	Describe methods used for assessing risk of bias of individual studies (including specification of whether this was done at the study or outcome level), and how this information is to be used in any data synthesis.	n/a, 2.8 Quality appraisal is reported as relevant to a qualitative SR #4
Summary measures	State the principal summary measures (e.g., risk ratio, difference in means).	n/a, 2.9 Phenomenon of interest is stated as relevant to a qualitative SR #4
Synthesis of results	Describe the methods of handling data and combining results of studies, if done, including measures of consistency (e.g., I^2) for each meta-analysis.	4-5
Risk of bias across studies	Specify any assessment of risk of bias that may affect the cumulative evidence (e.g., publication bias, selective reporting within studies).	n/a, further details are reported under section Amendments to the SR protocol #14 and supplement 4
Additional analyses	Describe methods of additional analyses (e.g., sensitivity or subgroup analyses, meta-regression), if done, indicating which were pre-specified.	5
**RESULTS**		

For peer review only

Section/topic	Checklist item	Reported on page #
Study selection	Give numbers of studies screened, assessed for eligibility, and included in the review, with reasons for exclusions at each stage, ideally with a flow diagram.	5
Study characteristics	For each study, present characteristics for which data were extracted (e.g., study size, PICOS, follow-up period) and provide the citations.	5
Risk of bias within studies	Present data on risk of bias of each study and, if available, any outcome-level assessment (see Item 12).	n/a, 3.3 Study appraisal is reported, #7
Results of individual studies	For all outcomes considered (benefits or harms), present, for each study: (a) simple summary data for each intervention group and (b) effect estimates and confidence intervals, ideally with a forest plot.	6, Table 1
Synthesis of results	Present results of each meta-analysis done, including confidence intervals and measures of consistency.	7-11
Risk of bias across studies	Present results of any assessment of risk of bias across studies (see Item 15).	n/a
Additional analysis	Give results of additional analyses, if done (e.g., sensitivity or subgroup analyses, meta-regression [see Item 16]).	n/a
**DISCUSSION**		
Summary of evidence	Summarize the main findings including the strength of evidence for each main outcome; consider their relevance to key groups (e.g., health care providers, users, and policy makers).	12-14
Limitations	Discuss limitations at study and outcome level (e.g., risk of bias), and at review level (e.g., incomplete retrieval of identified research, reporting bias).	14
Conclusions	Provide a general interpretation of the results in the context of other evidence, and implications for future research.	14
**FUNDING**		
Funding	Describe sources of funding for the systematic review and other support (e.g., supply of data); role of funders for the systematic review.	14

For peer review only

Supplement 2. Search strategy for Medline

Draft MEDLINE search- Ovid interface

1	osteoarthritis.mp. or exp Osteoarthritis, Hip/ or exp Osteoarthritis/ or exp Osteoarthritis, Knee/
2	(osteoarthriti* or osteo-arthriti* or osteoarthrotic or osteoarthros*).ti,ab.
3	(coxarthrosis or gonarthrosis).ti,ab.
4	"knee pain".mp.
5	"hip pain".mp.
6	"lower limb".mp.
7	exp Lower Extremity/ or "lower extremit*".mp.
8	(degenerative adj2 arthritis).ti,ab.
9	1 or 2 or 3 or 4 or 5 or 6 or 7 or 8
10	physical activity.mp. or exp Motor Activity/
11	exp Exercise/ or exp Exercise Therapy/ or exercise.mp.
12	exp Sports/ or sports.mp.
13	exp Life Style/ or exp Sedentary Lifestyle/ or sedentary.mp.
14	"non-exercis*".ti,ab.
15	"activities of daily living".mp. or exp "Activities of Daily Living"/
16	10 or 11 or 12 or 13 or 14 or 15
17	(maintain* or maintenance or support* or ongoing or "on-going" or adherence or reinforc* or comply* or compliance or "long-term" or adoption or engagement or avoidance or boost* or refresh* or remind* or promotion or promot* or "physical activity uptake" or "behavio* change" or "lifestyle change").ti,ab.
18	(barrier* or impediment or limit* or facilitator* or enablers or enabl* or motivators or motivat* or influenc* or factors or determinants).ti,ab.
19	facilitator*.mp.
20	barrier*.mp.
21	adherence.mp.
22	exp Motivation/ or motivators.mp.
23	social support.mp. or exp Social Support/
24	17 or 18 or 19 or 20 or 21 or 22 or 23
25	exp Qualitative Research/ or qualitative.mp.
26	(interview* or theme* or experience).mp. [mp=title, abstract, original title, name of substance word, subject heading word, keyword heading word, protocol supplementary concept word, rare disease supplementary concept word, unique identifier]
27	("content analysis" or "grounded theory" or "thematic analysis" or "phenomenological analysis" or phenomenolog* or narrative* or discourse or ethnograph*).ti,ab.
28	(("semi-structured" or semistructured or unstructured or informal or "in-depth" or indepth or "face-to-face" or structured or guide) adj3 (interview* or discussion* or

	questionnaire*)).ti,ab.
29	(focus group* or interview* or fieldwork or "field work" or triangulation or "data saturation" or "key informant").ti,ab.
30	25 or 26 or 27 or 28 or 29
31	9 and 16 and 24 and 30

Supplement 3. Findings: themes and supporting references.

1. Physical health

Barriers (#studies, #references)	References	Facilitators (#studies, #references)	References
Physical barriers and limitations<br>(9, 94)	*While pain was not attributed to their participation in the intervention the pain was described as having a major impact on their perceived opportunity to be physically active at present.* (Hammer, 2015)<br>*All participants discussed experiencing intense physical pain on a daily basis, and how it negatively affected their desire to be active... In addition to these limitations, participants spoke of fatiguing rapidly, which made considering physical activity as more of a challenge* (Stone & Baker, 2015)<br>*Stiffness and fatigue were barriers to exercising. "It was like my body was made of lead"* (Petursdottir, 2010)<br>*Two participants, who were both hikers, reported limiting effects of OA knee pain... A grandmother shared fears and concerns regarding dropping or falling on her grandchildren due to both hand and knee pain.* (Kabel, 2014)<br>*'But as soon as someone says 'let's go for a walk....' It's the last thing I want to do because it hurts too much ...'* (Kaptein, 2013)<br>*'...the day after I just couldn't cope, I was in so much pain'* (Fisken, 2015)<br>*'Exercise hurts. The pain was almost unbearable but I still carried on. Yes, it was very strenuous, but that's how it is, the pain becomes increasingly worse, I think...it just becomes more and more painful.'* (Thorstenson, 2006)<br>*Vi, Hilary, Ethel and Eileen all mentioned their being overweight as contributing to their knee symptom.* (Campbell, 2001)<br>*Ability was also limited by a perceived general lack of physical fitness, sometimes attributed to old age.* (Hendry, 2006)	PA for mobility, symptom relief and health<br>(9, 34)	*Some informants even expressed how their PA maintenance was partly motivated by the belief that PA could help them to postpone or maybe avoid surgery* (Hammer et al., 2015).<br>*"The main motivation to do all this is to prevent an operation to get a new hip" (participant with long-term goal)* (Veenhof et al., 2006)<br>*"I realised my mobility would get worse if I didn't do something about it so I started exercising". (2, 3, 20, 25)* (Hendry et al., 2006)<br>*"I feel like the Tin Man- that if I stop moving, I'll rust up and that will be it"* (Kaptein et al., 2013)<br>*As with the pain, however, the experience of less stiffness and more stamina turned out to be facilitating.* (Petursdottir et al., 2010)<br>*"The physiotherapist professionally guided me to feel less pain. It made me want to do exercises on my own."* (Stone & Baker et al., 2015)<br>*The perceived severity of knee symptoms was an important factor in motivation, with those experiencing severe pain and/or loss of mobility being most likely to continue to exercise.* (Campbell et al., 2001)<br>*...hip pain was highlighted as a common symptom, and several informants linked a perceived reduction in pain to their increased PA level, which represented an important incentive to maintain PA post-intervention.* (Hammer et al., 2015)<br>"Well, it is different now because, as I've already said, previously you exercised to maintain your level of fitness whereas now you exercise in order to regain your physical condition..." (Thorstenson et al., 2006)<br>*"Strenghtening your muscles..keeping your weight down...keeps you in shape"* (Fisken et al., 2015)<br>Disconfirming case: *Some participants who scored high on the Patient Global Assessment (eg, because they perceived less pain) did not continue with their activities, while some participants who scored low on the Patient Global Assessment (eg, because their pain remained the same) reported that their level of activities had increased considerably.* (Veenhof et al., 2006)

For peer review only - http://bmjopen.bmj.com/site/about/guidelines.xhtml

2. Intrapersonal factors: themes and references.

Major theme	Barriers (#studies, #references)	References	Facilitators (#studies, #references)	References
Experience and beliefs about PA and OA	PA as non-effective, harmful or of doubtful effectiveness (6, 36)	*Experiencing pain while exercising made it difficult to decide whether it was beneficial or counterproductive.* (Thorstenson, 2006)<br>*[Counter-advice or no recommendations] created further confusion about physical activity and the potential benefits for osteoarthritis.* (Stone & Baker, 2015)<br>*Many participants were worried that exercise was wearing out their joints...* (Hendry, 2006)<br>*..many participants* [were] *uncertain whether PA was good or bad for them when they have arthritis.* (Kaptein, 2013).<br>*If, however, the benefits of the physiotherapy were not perceived as sufficient…non-compliance was a rational outcome…* (Campbell, 2001)<br>*The two informants who had not managed to maintain an increased level of PA expressed how they had hoped for an improvement in their hip specific symptoms, which none of them had achieved.* (Hammer, 2015)	PA as beneficial (7, 60)	*They continued to undertake exercises… from which they perceived they would derive the most benefit.* (Campbell, 2001)<br>*"Keeps the body moving, takes your mind off it, it's good to be outside. Yea, keeping active, or else if you've got osteo, it can get you right down…"* (Fisken, 2015)<br>[Among maintainers] *it was generally described how PA, in addition to the physical effect, also significantly contributed to their psychological well-being.* (Hammer, 2015)<br>*They* [maintainers] *were more likely to have noticed beneficial effects on their OA knee, or general health and well-being as a result of exercise.* (Hendry, 2006)<br>*Other participants were motivated by the results of the exercise, not because they liked it or enjoyed it.* (Petursdottir, 2010)<br>*The informants expressed satisfaction and were convinced of the effectiveness of exercise.* (Thorstenson, 2006)<br>*"I really know these exercises have beneficial effects and that motivates me to continue with my exercises"* (Veenhof, 2006)
	OA beliefs (5, 17)	*"There's no cure, only pain relief"* (Hendry, 2006)<br>*"There is nothing that can be done about OA; therefore, I do nothing"* (Petursdottir, 2010)<br>*"...exercise can help, I am convinced about that, although it did not work for me…If one had started to exercise five or six years earlier, it might have helped."* (Thorstenson, 2006)<br>Another said, *"… if you're a very active person, especially professional athletes … they basically tell you you'll have arthritis when you get older."* (Kaptein, 2013)<br>*"I was having trouble with my knees every so often it did hurt you know with one thing and another. Working in the construction industry there is a lot of lifting and a lot kneeling you see and I felt well I wonder if that's got anything to do with it."* (Campbell, 2001)	Knowledge about PA (4, 15)	*It was described how increased knowledge and information about PA had led to an increased awareness of exercising and of doing this at a certain intensity and frequency…* (Hammer, 2015)<br>*Most of the participants had experienced being educated by their physical therapists.* (Petursdottir 2010)<br>*..many* [participants] *were unaware of specific osteoarthritis-related benefits and unsure of what activities would provide optimal self-management.* (Stone & Baker, 2015)<br>*Overall, most informants understood and acknowledged, but many undertook only a limited programme of exercise.* (Campbell, 2002)<br>[To experience coherence] *This conception contained statements about connecting knowledge about osteoarthritis with knowledge and experiences of exercise.* (Thorstenson, 2006)<br>Disconfirming case: *"You are in a vicious circle where you become less and less active, and the bones are grinding more and more due to muscle weakness. I could see that and I could understand it, but the knowledge has not helped me"* (Hammer, 2015)

Behavioural regulation and attitude	Resigned to OA (5, 10)	*Those who thought that arthritis was caused by immutable factors such as age, obesity and "wear and tear", tended to have a resigned attitude towards their arthritis.* (Campbell, 2001)<br>*...others had become resigned to their physical limitations... "I've accepted my limitations and said goodbye to going out."* (Hendry, 2006)<br>*"There is nothing that can be done about the OA; therefore, I do nothing"* (Petursdottir, 2010)<br>*...osteoarthritis-related pain can lead to disabling thoughts, which are precursors for adopting passive coping and learned helplessness.* (Stone & Baker, 2015)<br>*"... If one had started to exercise five or six years earlier, it might have helped."* (Thorstenson, 2006)	Keep going despite OA (7, 18)	*...those most likely to be continued compliers tended to believe that although there was no cure for arthritis, there were things they could do to minimise its impact, including the physiotherapy* (Campbell, 2002)<br>*Some participants were determined to take control of their disability and used exercise as a means of actively maintaining or improving their mobility. "I'm determined not to let my knee problem stop me from doing the things I want to do."* (Hendry, 2006)<br>*[To be prepared to persevere...] "I played 18 holes of golf and that is also quality of life. I refuse to sit a t home and navel gaze, I just won't"* (Thorstenson, 2006)<br>*"I worked out new ways to cope, to keep my arthritis from getting in the way too much"... They described the importance of not letting the OA control their lives, although its existence should be recognized and respected.* (Petursdottir, 2010).<br>*It appeared that all adherent participants were initially motivated to reach long-term goals.* (Veenhof, 2006)<br>*One participant shared that she continued to be physically active in her community, although she was concerned that others perceived her as being far older than her chronological age.* (Kabel, 2014).<br>*Occasionally participants mentioned adding new activities to their lives: "I learned how to ski about eight years ago. I always wanted to do it and I thought I'm not going to let this get me down"* (Kaptein, 2013)
	Lack of motivation (6, 14)	*"I suppose if there was a really good reason I would* [be strongly disciplined]*."* (Campbell, 2001)<br>*[The two non-maintainers also described obstacles for post-intervention PA...] the other described feeling a lack of motivation towards PA.* (Hammer, 2015)<br>[Reasons for not finding time to exercise...] *others freely admitted to being lazy or lacking motivation.* (Hendry, 2006)<br>*One of the participants seemed to lack the motivation to exercise, based on an overwhelming experience of boredom while exercising. She declared that she would never, ever exercise, no matter what. "It is dead boring, so I just don't do it and never will"* (Petursdottir, 2010)<br>*"You need to have the will to do it... when you are well you don't do it, and when you need to do it, then it hurts and therefore you don't do it (laughter)."* (Thorstenson, 2006)<br>*...all non-adherent participants reported a short-term initial goal or had no specific goal.* (Veenhof, 2006)	Adjustments, prioritization and personal effort (9, 41)	*The majority of informants described how they regularly adjusted their exercises and intensity in an attempt to strike a balance between continuously increasing intensity while at the same time considering the experienced pain.* (Hammer, 2015)<br>*They were eager to find activities and exercise that fitted them and, in many cases, adapted their exercises to their life with OA.* (Petursdottir, 2010).<br>*'My knees were getting really bad and I, so thought, well the only thing I can do really is to do aqua, which I did and I love it'* (Fisken, 2015).<br>*Prioritising exercise and making it part of a weekly routine helped some people to maintain their exercise habit. "... I try and say, OK well I'll go there [gym], have a shower and go shopping... I try to fit it in."* (Hendry, 2006)<br>*More important* [in increasing motivation] *was the willingness and ability to accommodate the exercises into everyday life.* (Campbell, 2001)<br>*"I continue with my exercises, they are integrated in my daily living."* (Veenhof, 2006)<br>*In order to deal with limited time and energy, many participants made tradeoffs. I've had to choose ... where I put my energy, and I know that some days I feel that all I've done is work, so that's kind of a bummer"* (Kaptein, 2013)<br>*He engaged in modified activity, not playing as aggressively as he wanted to, to avoid pain but did not opt out of the activity completely.* (Kabel, 2014)<br>*"Well I suppose to some extent it is up to yourself how much effort you wish to put into it, ... if I don't want to do anything then I don't think I ll benefit from any treatment. I suppose that at the end of the day the outcome of the treatment depends on no one but myself"* (Thorstenson, 2006)<br>Disconfirming case: *Later in their interviews both went on to admit some personal responsibility for their lack of compliance... "It's just excuses when it comes down to basics. I mean you know you could get up in the morning and do it between 6 or 7 or something like that."* (Cambpell, 2001)
	Lacking behavioural regulation (4, 23)	*Those who ceased exercising often cited conflict with regular routines to explain why continuing with exercises was not possible.* (Campbell, 2001)<br>*For others finding time to exercise was a low priority... "when I'm busy I forget."* (Hendry 2006)<br>*Despite recognising the importance of PA, it was considered optional or discretionary compared to essential roles such as work and family.*(Kaptein, 2013)<br>*"One is so occupied that it is very easy not to find time for exercise. Everything else takes precedence."* (Thorstenson, 2006)		

Emotions	OA-related distress (6, 23)	*6 of the 10 participants self-identified as having some type of embarrassment-related experience, usually general embarrassment and frustration over their physical limitations due to the OA pain.* (Kabel, 2014)<br>*Participants expressed depressing thoughts, referring to osteoarthritis as "mentally agonizing".* (Stone & Baker, 2015)<br>*"I don't know if you can imagine how it is to be confronted with things that you want to do but you are unable to. That is mentally stressful."* (Hammer, 2015)<br>*A few of the women mentioned 'paralyzing fatigue' as a major barrier for getting anything done and felt it might be related more to mental fatigue...* (Petursdottir, 2010)<br>*A few individuals noted a loss of their identity as an athletic or physically active person... they often tried to hide difficulties with activities from others.* (Kaptein, 2013)<br>*"It got worse and worse and I started falling down ... it's so embarrassing."* (Campbell, 2001)	Enjoyment (4, 22)	*Not surprisingly, people who enjoyed exercising were likely to continue; those that disliked it stopped. "I really do enjoy the gym; I look forward to going."* (Hendry, 2006)<br>*Some participants based their motivation on the fact that they liked PA and therefore had been physically active. "I have always enjoyed physical activity"* (Petursdottir, 2010)<br>*"The buoyancy... I like deep water... It takes the impact off your joints... it gives you freedom... if you've been sedentary and not able to move around... the water makes you feel wonderful"'* (Fisken, 2015).<br>*"I feel such a fool standing on one leg and going up and down on my own and I tends to drop it I do."* [non-maintainer] (Campbell, 2001)

3a. Social Environment: themes and references.

Major theme	Barriers (#studies, #references)	References	Facilitators (#studies, #references)	References
Health professionals	Lack of support from health professionals (5, 22)	*Sometimes the advice was vague or absent... Occasionally exercise was discouraged.* (Hendry et al., 2006)<br>*"So I go to the doctor and all he just simply done was put his hand on my knee, he said 'move your leg,... you are getting old, you've got rheumatism.'"* (Campbell et al., 2001)<br>*...physicians often provided them with counter advice or did not offer any recommendations...* (Stone & Baker, 2015)<br>*"They have not done it [encouraged exercising]"* (Petursdottir et al., 2010)<br>*"The instructor was not geared up for my particular disability [OA]... and I found it very stressful"* (Fisken et al., 2015)	Support from health professionals (8, 50)	*Advice from health professionals was mainly in favour of exercise and consisted of encouragement to exercise, advice about specific exercises, and referral to a gym. (Hendry et al., 2006)*<br>*The supervision by physical therapists highly influenced the informants' ability to progress in training intensity as the physical therapists verbally expressed their confidence in the participants and exhibited realistic expectations about their exercise abilities* (Hammer et al., 2015)<br>*All participants spoke about the instrumental role of health care providers in influencing and encouraging physical activity.* (Stone & Baker, 2015)<br>*"Well, I always say that my physical therapist is as good as any psychologist."* (Petursdottir et al., 2010)<br>*Overall, most informants understood and acknowledged, as they were instructed by the physiotherapist, that they should do the exercises often and regularly, but many undertook only a limited programme of exercise.* (Campbell et al., 2001)<br>*It appeared that all adherent participants reported that... the physiotherapists had a coaching role during intervention.* (Veenhof et al., 2006)<br>*"I think that [an instructor] is good because then you learn what to do so that you do not do it in the wrong way."* (Thorstenson et al., 2006)<br>*"...knowing that aqua is for people possibly who have arthritis... they ought to have.. an extra training course or something to fit, to accommodate that"* (Fisken et al., 2015)
Social support	Social comparison as demotivating (5, 15)	*Comparison with others with more limiting disease or a stoic attitude to knee symptoms all seemed to be associated with an attenuation of the motivation to comply"* (Campbell et al., 2001)<br>*"I found it very stressful to be honest because I felt like I had to do the same as the others and keep up..."*(Fisken et al., 2015).<br>*"They don't want to be dragged down by somebody that's not up to their standard I would think."* (Hendry et al., 2006)<br>*"I couldn't keep up with everyone else and felt like I was dragging them*	Social support facilitating PA (7, 43)	*The majority of informants described how they continued to exercise with others because of the mutual support and encouragement they hereby achieved...* (Hammer, 2015)<br>*The support, caring, and encouragement of others were among important external factors influencing how much the participants exercised.* (Petursdottir, 2010)<br>*"I think it's important to be with other people, how other people cope and that you're not alone and there are other people you know, in similar situations."* (Fisken, 2015).<br>*"I like the gym referral scheme because you're in a group of people who all have*

	Lack of social support (4, 8)	*behind."* (Kabel et al., 2014)<br>Disconfirming case: *Participants also gave examples of persisting with a painful activity and risking intensifying the pain because of social pressure or the desire to avoid embarrassment and disapproval...* (Kabel et al., 2014).<br><br>[Sedentary informants] *had been given scant encouragement to exercise.* (Hendry et al., 2006) [Regarding family's attitudes] *some of the women expressed having a hard time justifying to themselves and their families their need to spend time exercising.* (Petursdottir et al., 2010).<br>*Not only* [about half of the participants] *did not receive support from others to manage physically demanding activities at work, they often tried to hide difficulties with activities from others.* (Kaptein et al., 2013)<br>*"If perhaps my wife would work with me and you had a bit of competition..."* (Campbell et al., 2001)		*problems."* (Hendry, 2006)<br>*Eileen explained how difficult it was to continue the exercises programme since she stopped seeing the physiotherapist.* (Campbell, 2001)<br>*An important facilitator of PA and a strategy that helped some participants 'stay in the game' was having social support...* (Kaptein, 2013)<br>*"One of my friends who knows about my arthritis asked me if I ever exercise...Then she said she would work out with me if I wanted to. That was the first time I ever seriously thought about exercising.* (Stone & Baker, 2015)

Supplement 4. Amendments to the SR protocol

Confidence in the synthesised findings was not used due to ambiguities in the suggested process (ConQual[1]), i.e. regarding transparency and satisfactory justification of the assessment outcome. However, the studies-sources of each finding were checked. The three studies scoring "high" quality at both sets of criteria informed *all* themes, along with the medium quality studies.

Kappa statistic was not measured. The two researchers run the searches independently for all databases following the Medline search strategy. Because of differences in operators and options at different search engines, the number of studies differed at the stages preceding study selection. Each reviewer's full text selection stage was updated by the other researcher's findings. At this stage agreement was met for all included studies.

1. Munn Z, Porritt K, Lockwood C, et al. Establishing confidence in the output of qualitative research synthesis: the ConQual approach. *BMC Medical Research Methodology* 2014;14(1):1-7. doi: 10.1186/1471-2288-14-108

Supplement 1. PRISMA checklist of items to include when reporting a systematic review or meta-analysis

Section/topic	#	Checklist item	Reported on page #
**TITLE**			
Title	1	Identify the report as a systematic review, meta-analysis, or both.	1
**ABSTRACT**			
Structured summary	2	Provide a structured summary including, as applicable: background; objectives; data sources; study eligibility criteria, participants, and interventions; study appraisal and synthesis methods; results; limitations; conclusions and implications of key findings; systematic review registration number.	1
**INTRODUCTION**			
Rationale	3	Describe the rationale for the review in the context of what is already known.	2
Objectives	4	Provide an explicit statement of questions being addressed with reference to participants, interventions, comparisons, outcomes, and study design (PICOS).	3
**METHODS**			
Protocol and registration	5	Indicate if a review protocol exists, if and where it can be accessed (e.g., Web address), and, if available, provide registration information including registration number.	3
Eligibility criteria	6	Specify study characteristics (e.g., PICOS, length of follow-up) and report characteristics (e.g., years considered, language, publication status) used as criteria for eligibility, giving rationale.	3
Information sources	7	Describe all information sources (e.g., databases with dates of coverage, contact with study authors to identify additional studies) in the search and date last searched.	3
Search	8	Present full electronic search strategy for at least one database, including any limits used, such that it could be repeated.	3
Study selection	9	State the process for selecting studies (i.e., screening, eligibility, included in systematic review, and, if applicable, included in the meta-analysis).	4

For peer review only - http://bmjopen.bmj.com/site/about/guidelines.xhtml

Section/topic	#	Checklist item	Reported on page #
Data collection process	10	Describe method of data extraction from reports (e.g., piloted forms, independently, in duplicate) and any processes for obtaining and confirming data from investigators.	4
Data items	11	List and define all variables for which data were sought (e.g., PICOS, funding sources) and any assumptions and simplifications made.	4
Risk of bias in individual studies	12	Describe methods used for assessing risk of bias of individual studies (including specification of whether this was done at the study or outcome level), and how this information is to be used in any data synthesis.	n/a, 2.8 Quality appraisal is reported as relevant to a qualitative SR #4
Summary measures	13	State the principal summary measures (e.g., risk ratio, difference in means).	n/a, 2.9 Phenomenon of interest is stated as relevant to a qualitative SR #4
Synthesis of results	14	Describe the methods of handling data and combining results of studies, if done, including measures of consistency (e.g., I^2) for each meta-analysis.	4-5
Risk of bias across studies	15	Specify any assessment of risk of bias that may affect the cumulative evidence (e.g., publication bias, selective reporting within studies).	n/a, further details are reported under section Amendments to the SR protocol #14 and supplement 4
Additional analyses	16	Describe methods of additional analyses (e.g., sensitivity or subgroup analyses, meta-regression), if done, indicating which were pre-specified.	5
**RESULTS**			
Study selection	17	Give numbers of studies screened, assessed for eligibility, and included in the	5

For peer review only - http://bmjopen.bmj.com/site/about/guidelines.xhtml

Section/topic	#	Checklist item	Reported on page #
		review, with reasons for exclusions at each stage, ideally with a flow diagram.	
Study characteristics	18	For each study, present characteristics for which data were extracted (e.g., study size, PICOS, follow-up period) and provide the citations.	5
Risk of bias within studies	19	Present data on risk of bias of each study and, if available, any outcome-level assessment (see Item 12).	n/a, 3.3 Study appraisal is reported, #7
Results of individual studies	20	For all outcomes considered (benefits or harms), present, for each study: (a) simple summary data for each intervention group and (b) effect estimates and confidence intervals, ideally with a forest plot.	6, Table 1
Synthesis of results	21	Present results of each meta-analysis done, including confidence intervals and measures of consistency.	7-11
Risk of bias across studies	22	Present results of any assessment of risk of bias across studies (see Item 15).	n/a
Additional analysis	23	Give results of additional analyses, if done (e.g., sensitivity or subgroup analyses, meta-regression [see Item 16]).	n/a
**DISCUSSION**			
Summary of evidence	24	Summarize the main findings including the strength of evidence for each main outcome; consider their relevance to key groups (e.g., health care providers, users, and policy makers).	12-14
Limitations	25	Discuss limitations at study and outcome level (e.g., risk of bias), and at review level (e.g., incomplete retrieval of identified research, reporting bias).	14
Conclusions	26	Provide a general interpretation of the results in the context of other evidence, and implications for future research.	14
**FUNDING**			
Funding	27	Describe sources of funding for the systematic review and other support (e.g., supply of data); role of funders for the systematic review.	14

For peer review only - http://bmjopen.bmj.com/site/about/guidelines.xhtml

Supplement 2. Search strategy for Medline

Draft MEDLINE search- Ovid interface

1	osteoarthritis.mp. or exp Osteoarthritis, Hip/ or exp Osteoarthritis/ or exp Osteoarthritis, Knee/
2	(osteoarthriti* or osteo-arthriti* or osteoarthrotic or osteoarthros*).ti,ab.
3	(coxarthrosis or gonarthrosis).ti,ab.
4	"knee pain".mp.
5	"hip pain".mp.
6	"lower limb".mp.
7	exp Lower Extremity/ or "lower extremit*".mp.
8	(degenerative adj2 arthritis).ti,ab.
9	1 or 2 or 3 or 4 or 5 or 6 or 7 or 8
10	physical activity.mp. or exp Motor Activity/
11	exp Exercise/ or exp Exercise Therapy/ or exercise.mp.
12	exp Sports/ or sports.mp.
13	exp Life Style/ or exp Sedentary Lifestyle/ or sedentary.mp.
14	"non-exercis*".ti,ab.
15	"activities of daily living".mp. or exp "Activities of Daily Living"/
16	10 or 11 or 12 or 13 or 14 or 15
17	(maintain* or maintenance or support* or ongoing or "on-going" or adherence or reinforc* or comply* or compliance or "long-term" or adoption or engagement or avoidance or boost* or refresh* or remind* or promotion or promot* or "physical activity uptake" or "behavio* change" or "lifestyle change").ti,ab.
18	(barrier* or impediment or limit* or facilitator* or enablers or enabl* or motivators or motivat* or influenc* or factors or determinants).ti,ab.
19	facilitator*.mp.

20 barrier*.mp.
21 adherence.mp.
22 exp Motivation/ or motivators.mp.
23 social support.mp. or exp Social Support/
24 17 or 18 or 19 or 20 or 21 or 22 or 23
25 exp Qualitative Research/ or qualitative.mp.
26 (interview* or theme* or experience).mp. [mp=title, abstract, original title, name of substance word, subject heading word, keyword heading word, protocol supplementary concept word, rare disease supplementary concept word, unique identifier]
27 ("content analysis" or "grounded theory" or "thematic analysis" or "phenomenological analysis" or phenomenolog* or narrative* or discourse or ethnograph*).ti,ab.
28 (("semi-structured" or semistructured or unstructured or informal or "in-depth" or indepth or "face-to-face" or structured or guide) adj3 (interview* or discussion* or questionnaire*)).ti,ab.
29 (focus group* or interview* or fieldwork or "field work" or triangulation or "data saturation" or "key informant").ti,ab.
30 25 or 26 or 27 or 28 or 29
31 9 and 16 and 24 and 30

Supplement 3. Findings: themes and supporting references.

1. Physical health

Barriers (#studies, #references)	References	Facilitators (#studies, #references)	References
Physical barriers and limitations (9, [illegible])	*While pain was not attributed to their participation in the intervention the pain was described as having a major impact on their perceived opportunity to be physically active at present.* (Hammer, 2015)<br>*All participants discussed experiencing intense physical pain on a daily basis, and how it negatively affected their desire to be active… In addition to these limitations, participants spoke of fatiguing rapidly, which made considering physical activity as more of a challenge* (Stone & Baker, 2015)<br>*Stiffness and fatigue were barriers to exercising. "It was like my body was made of lead"* (Petursdottir, 2010)<br>*Two participants, who were both hikers, reported limiting effects of OA knee pain… A grandmother shared fears and concerns regarding dropping or falling on her grandchildren due to both hand and knee pain.* (Kabel, 2014)<br>*'But as soon as someone says 'let's go for a walk….' It's the last thing I want to do because it hurts too much …'* (Kaptein, 2013)<br>*'…the day after I just couldn't cope, I was in so much pain'* (Fisken, 2015)<br>*'Exercise hurts. The pain was almost unbearable but I still carried on. Yes, it was very strenuous, but that's how it is, the pain becomes increasingly worse, I think…it just becomes more and more painful.'* (Thorstenson, 2006)<br>*Vi, Hilary, Ethel and Eileen all mentioned their being overweight as contributing to their knee symptom.* (Campbell, 2001)<br>*Ability was also limited by a perceived general lack of physical fitness, sometimes attributed to old age.* (Hendry, 2006)	PA for mobility, symptom relief and health (9, 34)	*Some informants even expressed how their PA maintenance was partly motivated by the belief that PA could help them to postpone or maybe avoid surgery* (Hammer et al., 2015).<br>*"The main motivation to do all this is to prevent an operation to get a new hip" (participant with long-term goal)* (Veenhof et al., 2006)<br>*"I realised my mobility would get worse if I didn't do something about it so I started exercising". (2, 3, 20, 25)* (Hendry et al., 2006)<br>*"I feel like the Tin Man- that if I stop moving, I'll rust up and that will be it"* (Kaptein et al., 2013)<br>*As with the pain, however, the experience of less stiffness and more stamina turned out to be facilitating.* (Petursdottir et al., 2010)<br>*"The physiotherapist professionally guided me to feel less pain. It made me want to do exercises on my own."* (Stone & Baker et al., 2015)<br>*The perceived severity of knee symptoms was an important factor in motivation, with those experiencing severe pain and/or loss of mobility being most likely to continue to exercise.* (Campbell et al., 2001)<br>*…hip pain was highlighted as a common symptom, and several informants linked a perceived reduction in pain to their increased PA level, which represented an important incentive to maintain PA post-intervention.* (Hammer et al., 2015)<br>"Well, it is different now because, as I've already said, previously you exercised to maintain your level of fitness whereas now you exercise in order to regain your physical condition…" (Thorstenson et al., 2006)<br>*"Strenghtening your muscles..keeping your weight down…keeps you in shape"* (Fisken et al., 2015)<br>Disconfirming case: *Some participants who scored high on the Patient Global Assessment (eg, because they perceived less pain) did not continue with their activities, while some participants who scored low on the Patient Global Assessment (eg, because their pain remained the same) reported that their level of activities had increased considerably.* (Veenhof et al., 2006)

For peer review only - http://bmjopen.bmj.com/site/about/guidelines.xhtml

2. Intrapersonal factors: themes and references.

Major theme	Barriers (#studies, #references)	References	Facilitators (#studies, #references)	References
Experience and beliefs about PA and OA	PA as non-effective, harmful or of doubtful effectiveness (6, 36)	*Experiencing pain while exercising made it difficult to decide whether it was beneficial or counterproductive.* (Thorstenson, 2006)<br>*[Counter-advice or no recommendations] created further confusion about physical activity and the potential benefits for osteoarthritis.* (Stone & Baker, 2015)<br>*Many participants were worried that exercise was wearing out their joints...* (Hendry, 2006)<br>*..many participants* [were] *uncertain whether PA was good or bad for them when they have arthritis.* (Kaptein, 2013).<br>*If, however, the benefits of the physiotherapy were not perceived as sufficient…non-compliance was a rational outcome…* (Campbell, 2001)<br>*The two informants who had not managed to maintain an increased level of PA expressed how they had hoped for an improvement in their hip specific symptoms, which none of them had achieved.* (Hammer, 2015)	PA as beneficial (7, 60)	*They continued to undertake exercises… from which they perceived they would derive the most benefit.* (Campbell, 2001)<br>*"Keeps the body moving, takes your mind off it, it's good to be outside. Yea, keeping active, or else if you've got osteo, it can get you right down…"* (Fisken, 2015)<br>[Among maintainers] *it was generally described how PA, in addition to the physical effect, also significantly contributed to their psychological well-being.* (Hammer, 2015)<br>*They* [maintainers] *were more likely to have noticed beneficial effects on their OA knee, or general health and well-being as a result of exercise.* (Hendry, 2006)<br>*Other participants were motivated by the results of the exercise, not because they liked it or enjoyed it.* (Petursdottir, 2010)<br>*The informants expressed satisfaction and were convinced of the effectiveness of exercise.* (Thorstenson, 2006)<br>*"I really know these exercises have beneficial effects and that motivates me to continue with my exercises"* (Veenhof, 2006)
	OA beliefs (5, 17)	*"There's no cure, only pain relief"* (Hendry, 2006)<br>*"There is nothing that can be done about OA; therefore, I do nothing"* (Petursdottir, 2010)<br>*"...exercise can help, I am convinced about that, although it did not work for me…If one had started to exercise five or six years earlier, it might have helped."* (Thorstenson, 2006)<br>Another said, *"… if you're a very active person, especially professional athletes … they basically tell you you'll have arthritis when you get older."* (Kaptein, 2013)<br>*"I was having trouble with my knees every so often it did hurt you know with one thing and another. Working in the construction industry there is a lot of lifting and a lot kneeling you see and I felt well I wonder if that's got anything to do with it."* (Campbell, 2001)	Knowledge about PA (4, 15)	*It was described how increased knowledge and information about PA had led to an increased awareness of exercising and of doing this at a certain intensity and frequency…* (Hammer, 2015)<br>*Most of the participants had experienced being educated by their physical therapists.* (Petursdottir 2010)<br>*..many* [participants] *were unaware of specific osteoarthritis-related benefits and unsure of what activities would provide optimal self-management.* (Stone & Baker, 2015)<br>*Overall, most informants understood and acknowledged, but many undertook only a limited programme of exercise.* (Campbell, 2002)<br>[To experience coherence] *This conception contained statements about connecting knowledge about osteoarthritis with knowledge and experiences of exercise.* (Thorstenson, 2006)<br>Disconfirming case: *"You are in a vicious circle where you become less and less active, and the bones are grinding more and more due to muscle weakness. I could see that and I could understand it, but the knowledge has not helped me"* (Hammer, 2015)

For peer review only - http://bmjopen.bmj.com/site/about/guidelines.xhtml

Behavioural regulation and attitude	Resigned to OA (5, 10)	*Those who thought that arthritis was caused by immutable factors such as age, obesity and "wear and tear", tended to have a resigned attitude towards their arthritis.* (Campbell, 2001)<br>*...others had become resigned to their physical limitations... "I've accepted my limitations and said goodbye to going out."* (Hendry, 2006)<br>*"There is nothing that can be done about the OA; therefore, I do nothing"* (Petursdottir, 2010)<br>*...osteoarthritis-related pain can lead to disabling thoughts, which are precursors for adopting passive coping and learned helplessness.* (Stone & Baker, 2015)<br>*"... If one had started to exercise five or six years earlier, it might have helped."* (Thorstenson, 2006)	Keep going despite OA (7, 18)	*...those most likely to be continued compliers tended to believe that although there was no cure for arthritis, there were things they could do to minimise its impact, including the physiotherapy* (Campbell, 2002)<br>*Some participants were determined to take control of their disability and used exercise as a means of actively maintaining or improving their mobility. "I'm determined not to let my knee problem stop me from doing the things I want to do."* (Hendry, 2006)<br>*[To be prepared to persevere...] "I played 18 holes of golf and that is also quality of life. I refuse to sit a t home and navel gaze, I just won't"* (Thorstenson, 2006)<br>*"I worked out new ways to cope, to keep my arthritis from getting in the way too much"... They described the importance of not letting the OA control their lives, although its existence should be recognized and respected.* (Petursdottir, 2010).<br>*It appeared that all adherent participants were initially motivated to reach long-term goals.* (Veenhof, 2006)<br>*One participant shared that she continued to be physically active in her community, although she was concerned that others perceived her as being far older than her chronological age.* (Kabel, 2014).<br>*Occasionally participants mentioned adding new activities to their lives: "I learned how to ski about eight years ago. I always wanted to do it and I thought I'm not going to let this get me down"* (Kaptein, 2013)
	Lack of motivation (6, 14)	*"I suppose if there was a really good reason I would* [be strongly disciplined]*."* (Campbell, 2001)<br>*[The two non-maintainers also described obstacles for post-intervention PA...] the other described feeling a lack of motivation towards PA.* (Hammer, 2015)<br>[Reasons for not finding time to exercise...] *others freely admitted to being lazy or lacking motivation.* (Hendry, 2006)<br>*One of the participants seemed to lack the motivation to exercise, based on an overwhelming experience of boredom while exercising. She declared that she would never, ever exercise, no matter what. "It is dead boring, so I just don't do it and never will"* (Petursdottir, 2010)<br>*"You need to have the will to do it... when you are well you don't do it, and when you need to do it, then it hurts and therefore you don't do it (laughter)."* (Thorstenson, 2006)<br>*...all non-adherent participants reported a short-term initial goal or had no specific goal.* (Veenhof, 2006)	Adjustments, prioritization and personal effort (9, 41)	*The majority of informants described how they regularly adjusted their exercises and intensity in an attempt to strike a balance between continuously increasing intensity while at the same time considering the experienced pain.* (Hammer, 2015)<br>*They were eager to find activities and exercise that fitted them and, in many cases, adapted their exercises to their life with OA.* (Petursdottir, 2010).<br>*'My knees were getting really bad and I, so thought, well the only thing I can do really is to do aqua, which I did and I love it'* (Fisken, 2015).<br>*Prioritising exercise and making it part of a weekly routine helped some people to maintain their exercise habit. "... I try and say, OK well I'll go there [gym], have a shower and go shopping... I try to fit it in."* (Hendry, 2006)<br>*More important* [in increasing motivation] *was the willingness and ability to accommodate the exercises into everyday life.* (Campbell, 2001)<br>*"I continue with my exercises, they are integrated in my daily living."* (Veenhof, 2006)<br>*In order to deal with limited time and energy, many participants made tradeoffs. I've had to choose ... where I put my energy, and I know that some days I feel that all I've done is work, so that's kind of a bummer"* (Kaptein, 2013)<br>*He engaged in modified activity, not playing as aggressively as he wanted to, to avoid pain but did not opt out of the activity completely.* (Kabel, 2014)<br>*"Well I suppose to some extent it is up to yourself how much effort you wish to put into it, ... if I don't want to do anything then I don't think I ll benefit from any treatment. I suppose that at the end of the day the outcome of the treatment depends on no one but myself"* (Thorstenson, 2006)<br>Disconfirming case: *Later in their interviews both went on to admit some personal responsibility for their lack of compliance... "It's just excuses when it comes down to basics. I mean you know you could get up in the morning and do it between 6 or 7 or something like that."* (Cambpell, 2001)
	Lacking behavioural regulation (4, 23)	*Those who ceased exercising often cited conflict with regular routines to explain why continuing with exercises was not possible.* (Campbell, 2001)<br>*For others finding time to exercise was a low priority... "when I'm busy I forget."* (Hendry 2006)<br>*Despite recognising the importance of PA, it was considered optional or discretionary compared to essential roles such as work and family.*(Kaptein, 2013)<br>*"One is so occupied that it is very easy not to find time for exercise. Everything else takes precedence."* (Thorstenson, 2006)		

Emotions	OA-related distress (6, 23)	*6 of the 10 participants self-identified as having some type of embarrassment-related experience, usually general embarrassment and frustration over their physical limitations due to the OA pain.* (Kabel, 2014)<br>*Participants expressed depressing thoughts, referring to osteoarthritis as "mentally agonizing".* (Stone & Baker, 2015)<br>*"I don't know if you can imagine how it is to be confronted with things that you want to do but you are unable to. That is mentally stressful."* (Hammer, 2015)<br>*A few of the women mentioned 'paralyzing fatigue' as a major barrier for getting anything done and felt it might be related more to mental fatigue...* (Petursdottir, 2010)<br>*A few individuals noted a loss of their identity as an athletic or physically active person... they often tried to hide difficulties with activities from others.* (Kaptein, 2013)<br>*"It got worse and worse and I started falling down ... it's so embarrassing."* (Campbell, 2001)	Enjoyment (4, 22)	*Not surprisingly, people who enjoyed exercising were likely to continue; those that disliked it stopped. "I really do enjoy the gym; I look forward to going."* (Hendry, 2006)<br>*Some participants based their motivation on the fact that they liked PA and therefore had been physically active. "I have always enjoyed physical activity"* (Petursdottir, 2010)<br>*"The buoyancy...I like deep water...It takes the impact off your joints...it gives you freedom...if you've been sedentary and not able to move around...the water makes you feel wonderful"'* (Fisken, 2015).<br>*"I feel such a fool standing on one leg and going up and down on my own and I tends to drop it I do."* [non-maintainer] (Campbell, 2001)

3a. Social Environment: themes and references.

Major theme	Barriers (#studies, #references)	References	Facilitators (#studies, #references)	References
Health professionals	Lack of support from health professionals (5, 22)	*Sometimes the advice was vague or absent... Occasionally exercise was discouraged.* (Hendry et al., 2006)<br>*"So I go to the doctor and all he just simply done was put his hand on my knee, he said 'move your leg,...you are getting old, you've got rheumatism.'"* (Campbell et al., 2001)<br>*...physicians often provided them with counter advice or did not offer any recommendations...* (Stone & Baker, 2015)<br>*"They have not done it [encouraged exercising]"* (Petursdottir et al., 2010)<br>*"The instructor was not geared up for my particular disability [OA]... and I found it very stressful"* (Fisken et al., 2015)	Support from health professionals (8, 50)	*Advice from health professionals was mainly in favour of exercise and consisted of encouragement to exercise, advice about specific exercises, and referral to a gym. (Hendry et al., 2006)*<br>*The supervision by physical therapists highly influenced the informants' ability to progress in training intensity as the physical therapists verbally expressed their confidence in the participants and exhibited realistic expectations about their exercise abilities* (Hammer et al., 2015)<br>*All participants spoke about the instrumental role of health care providers in influencing and encouraging physical activity.* (Stone & Baker, 2015)<br>*"Well, I always say that my physical therapist is as good as any psychologist."* (Petursdottir et al., 2010)<br>*Overall, most informants understood and acknowledged, as they were instructed by the physiotherapist, that they should do the exercises often and regularly, but many undertook only a limited programme of exercise.* (Campbell et al., 2001)<br>*It appeared that all adherent participants reported that... the physiotherapists had a coaching role during intervention.* (Veenhof et al., 2006)<br>*"I think that [an instructor] is good because then you learn what to do so that you do not do it in the wrong way."* (Thorstenson et al., 2006)<br>*"...knowing that aqua is for people possibly who have arthritis...they ought to have.. an extra training course or something to fit, to accommodate that"* (Fisken et al., 2015)
Social support	Social comparison as demotivating (5, 15)	*Comparison with others with more limiting disease or a stoic attitude to knee symptoms all seemed to be associated with an attenuation of the motivation to comply"* (Campbell et al., 2001)<br>*"I found it very stressful to be honest because I felt like I had to do the same as the others and keep up... "*(Fisken et al., 2015).<br>*"They don't want to be dragged down by somebody that's not up to their standard I would think."* (Hendry et al., 2006)<br>*"I couldn't keep up with everyone else and felt like I was dragging them*	Social support facilitating PA (7, 43)	*The majority of informants described how they continued to exercise with others because of the mutual support and encouragement they hereby achieved...* (Hammer, 2015)<br>*The support, caring, and encouragement of others were among important external factors influencing how much the participants exercised.* (Petursdottir, 2010)<br>*"I think it's important to be with other people, how other people cope and that you're not alone and there are other people you know, in similar situations."* (Fisken, 2015).<br>*"I like the gym referral scheme because you're in a group of people who all have*

For peer review only - http://bmjopen.bmj.com/site/about/guidelines.xhtml

		behind." (Kabel et al., 2014)<br>Disconfirming case: *Participants also gave examples of persisting with a painful activity and risking intensifying the pain because of social pressure or the desire to avoid embarrassment and disapproval...* (Kabel et al., 2014).		*problems."* (Hendry, 2006)<br>*Eileen explained how difficult it was to continue the exercises programme since she stopped seeing the physiotherapist.* (Campbell, 2001)<br>*An important facilitator of PA and a strategy that helped some participants 'stay in the game' was having social support...* (Kaptein, 2013)<br>*"One of my friends who knows about my arthritis asked me if I ever exercise...Then she said she would work out with me if I wanted to. That was the first time I ever seriously thought about exercising.* (Stone & Baker, 2015)
	Lack of social support (4, 8)	[Sedentary informants] *had been given scant encouragement to exercise.* (Hendry et al., 2006) [Regarding family's attitudes] *some of the women expressed having a hard time justifying to themselves and their families their need to spend time exercising.* (Petursdottir et al., 2010).<br>*Not only* [about half of the participants] *did not receive support from others to manage physically demanding activities at work, they often tried to hide difficulties with activities from others.* (Kaptein et al., 2013)<br>*"If perhaps my wife would work with me and you had a bit of competition..."* (Campbell et al., 2001)		

Supplement 4. Amendments to the SR protocol

Confidence in the synthesised findings was not used due to ambiguities in the suggested process (ConQual[1]), i.e. regarding transparency and satisfactory justification of the assessment outcome. However, the studies-sources of each finding were checked. The three studies scoring "high" quality at both sets of criteria informed *all* themes, along with the medium quality studies.

Kappa statistic was not measured. The two researchers run the searches independently for all databases following the Medline search strategy. Because of differences in operators and options at different search engines, the number of studies differed at the stages preceding study selection. Each reviewer's full text selection stage was updated by the other researcher's findings. At this stage agreement was met for all included studies.

1. Munn Z, Porritt K, Lockwood C, et al. Establishing confidence in the output of qualitative research synthesis: the ConQual approach. *BMC Medical Research Methodology* 2014;14(1):1-7. doi: 10.1186/1471-2288-14-108
